# Targeting the CoREST complex with dual histone deacetylase and demethylase inhibitors

**DOI:** 10.1038/s41467-017-02242-4

**Published:** 2018-01-04

**Authors:** Jay H. Kalin, Muzhou Wu, Andrea V. Gomez, Yun Song, Jayanta Das, Dawn Hayward, Nkosi Adejola, Mingxuan Wu, Izabela Panova, Hye Jin Chung, Edward Kim, Holly J. Roberts, Justin M. Roberts, Polina Prusevich, Jeliazko R. Jeliazkov, Shourya S. Roy Burman, Louise Fairall, Charles Milano, Abdulkerim Eroglu, Charlotte M. Proby, Albena T. Dinkova-Kostova, Wayne W. Hancock, Jeffrey J. Gray, James E. Bradner, Sergio Valente, Antonello Mai, Nicole M. Anders, Michelle A. Rudek, Yong Hu, Byungwoo Ryu, John W. R. Schwabe, Andrea Mattevi, Rhoda M. Alani, Philip A. Cole

**Affiliations:** 1 Division of Genetics, Departments of Medicine and Biological Chemistry and Molecular Pharmacology, Harvard Medical School and Brigham and Women’s Hospital, Boston, MA 02115 USA; 2 Department of Pharmacology and Molecular Sciences, Johns Hopkins University School of Medicine, Baltimore, MD 21205 USA; 3 Department of Dermatology, Boston University School of Medicine, Boston, MA 02118 USA; 4 Department of Biology and Biotechnology, University of Pavia, 27100 Pavia, Italy; 5 Department of Molecular and Cell Biology, University of Leicester, Leicester, LE1 9HN UK; 6 Department of Medical Oncology, Dana-Farber Cancer Institute, Boston, MA 02215 USA; 7 Program in Molecular Biophysics, Johns Hopkins University, Baltimore, MD 21218 USA; 8 Department of Chemical and Biomolecular Engineering, Johns Hopkins University, Baltimore, MD 21218 USA; 9 Division of Cancer Research, Jacqui Wood Cancer Centre, University of Dundee, Dundee, DD1 9SY UK; 10 Department of Pathology and Laboratory Medicine, University of Pennsylvania School of Medicine, Philadelphia, PA 19104 USA; 11 Sidney Kimmel Comprehensive Cancer Center, Johns Hopkins University School of Medicine, Baltimore, MD 21205 USA; 12 Pasteur Institute, Cenci-Bolognetti Foundation, Department of Drug Chemistry and Technologies, Sapienza University of Rome, 00185 Rome, Italy; 13 Department of Oncology, BioDuro LLC, Shanghai, 200131 China

**Keywords:** Multienzyme complexes, Chemical tools, Histone post-translational modifications

## Abstract

Here we report corin, a synthetic hybrid agent derived from the class I HDAC inhibitor (entinostat) and an LSD1 inhibitor (tranylcypromine analog). Enzymologic analysis reveals that corin potently targets the CoREST complex and shows more sustained inhibition of CoREST complex HDAC activity compared with entinostat. Cell-based experiments demonstrate that corin exhibits a superior anti-proliferative profile against several melanoma lines and cutaneous squamous cell carcinoma lines compared to its parent monofunctional inhibitors but is less toxic to melanocytes and keratinocytes. CoREST knockdown, gene expression, and ChIP studies suggest that corin’s favorable pharmacologic effects may rely on an intact CoREST complex. Corin was also effective in slowing tumor growth in a melanoma mouse xenograft model. These studies highlight the promise of a new class of two-pronged hybrid agents that may show preferential targeting of particular epigenetic regulatory complexes and offer unique therapeutic opportunities.

## Abstract (web summary)

Alteration of the epigenetic landscape has been implicated in several disease processes, where targeting histone modifiers may have therapeutic applications. Here the authors report a bifunctional small molecule inhibitor that simultaneously targets the deacetylase (HDAC1) and demethylase (LSD1) activities of the CoREST complex.

## Introduction

**Fig. 1:**
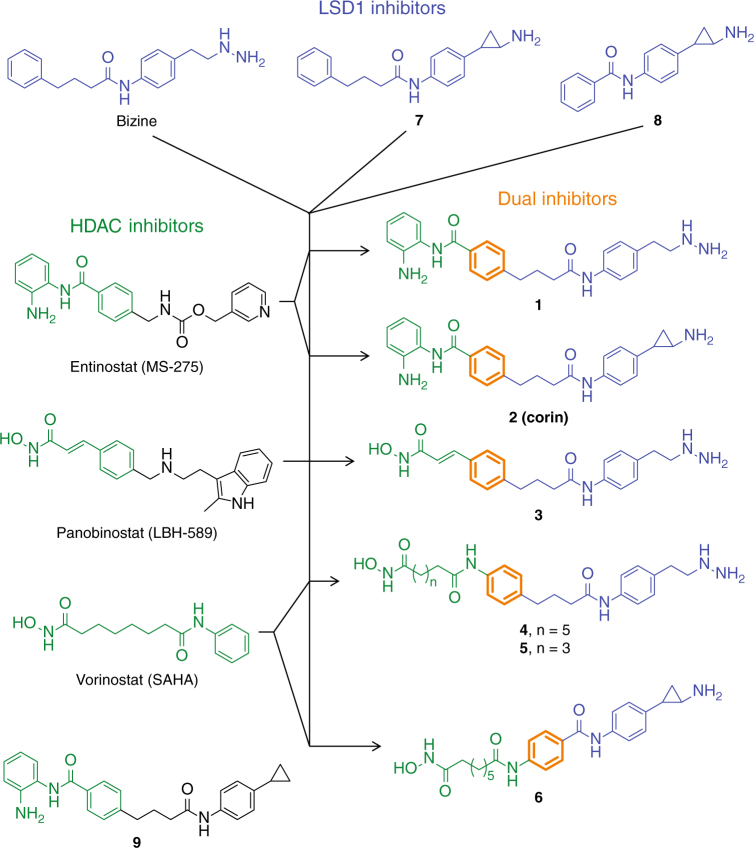
Strategy for combining the pharmacophores of clinically used HDAC inhibitors and a preclinical LSD1 inhibitor to generate dual action HDAC-LSD1 inhibitors. Blue—incorporated LSD1 inhibitor features, Green—incorporated HDAC inhibitor features, Orange—shared structural features, Black—features not incorporated into dual inhibitors

Epigenetic regulation of gene expression by histone modification has emerged as a major facet of physiologic and disease processes. As a result, there has been intense interest in developing epigenetic therapies leading to the discovery of small molecule agents that target proteins involved in histone modification^1, 2^. Several histone deacetylase (HDAC) inhibitors like vorinastat and panobinostat (Fig. 1) are now approved drugs for a specialized group of hematologic malignancies but not yet for a wider range of cancer types including solid tumors^3^. One of the conceptual challenges in targeting HDACs is that even selective class I HDAC inhibitors such as entinostat (MS-275) (Fig. 1) likely impact these deacetylase activities indiscriminately across a range of distinct HDAC-containing multiprotein complexes^4, 5^. Such broad cellular effects may result in a narrow therapeutic window between disease efficacy and toxicity. Among HDAC complexes, the CoREST complex, which includes HDAC1 or its close paralog HDAC2, the scaffolding protein CoREST, and lysine specific demethylase 1 (LSD1) has attracted special interest^5, 6^. The HDAC1/2 and LSD1 enzymatic activities within the CoREST complex are commonly associated with silencing gene expression and contribute to cancer and other diseases^2, 3, 5^. Several classes of LSD1 demethylase inhibitors have been reported, and the best characterized are analogs of tranylcypromine and phenelzine, established monoamine oxidase (MAO) mechanism-based inactivators^6–11^ (Supplementary Fig. 1). These MAO inhibitor analogs can be oxidized by LSD1’s active site flavin cofactor (FAD) and converted to reactive electrophiles that undergo covalent bond formation with FAD resulting in an irreversible blockade of LSD1^8, 10, 12^. These tranylcypromine and phenelzine analogs have been shown to have anti-tumor potential alone and in combination with HDAC inhibitors in preclinical settings^6–9^.

We considered the possibility that dual action LSD1/HDAC inhibitory compounds might be pharmaceutically advantageous. By comprehensively blocking the CoREST complex, dual LSD1/HDAC inhibitors could show a uniquely favorable profile of pharmacologic action with an improved therapeutic window relative to pure HDAC inhibitors. However, the challenges of developing pharmacologically attractive dual action CoREST inhibitors include: retaining high potency and specificity within one compound for the two enzyme targets, achieving an approximate balance in targeting LSD1 and HDAC1/2 in the CoREST complex, and managing the size and polarity of such hybrid compounds to have a favorable pharmacologic profile.

## Results

### Design and enzymatic analysis of dual LSD1/HDAC inhibitors

Interestingly, compound 4SC-202 is proposed to act as a dual HDAC/LSD1 inhibitor^13^. In our assays, we did not observe significant inhibition of LSD1 with 4SC-202 (Supplementary Fig. 2) so it would appear to have a different mechanism of action from the inhibitors we report here (see below).

As a three-dimensional structure of the core CoREST complex was not available to aid our design, we based our strategy on current knowledge of pharmacophore characteristics of existing unifunctional HDAC and LSD1 active site targeting compounds. Inspection of several known HDAC and LSD1 inhibitor scaffolds suggests that a common phenyl ring could serve as the central element of an effective dual LSD1/HDAC inhibitor as shown (Fig. 1). Thus, we designed and synthesized hybrid compounds **1–6** that contained standard HDAC zinc binding groups, a benzamide (as in MS-275/entinostat) or a hydroxamic acid (as in SAHA/vorinostat and LBH589/panobinostat), tethered to an amine oxidase warhead, either phenelzine or tranylcypromine (Fig. 1). Details of the synthetic routes, related to prior efforts on related molecules, are shown in the Supplementary Methods. Compound **7** was new to this study, but is the cyclopropylamine analog of the established LSD1 inhibitor bizine^7^. We also synthesized compounds **8** and **9** as controls that were closely related to **6** and **2** respectively, but included subtle warhead disruptions. Note that each of the designed bifunctional compounds **1–6** have molecular weights (< 500 g mol^−1^) and hydrophobicity (0.5 < cLogP < 3) that are in the drug-like range (Supplementary Table 1)^14^.

We proceeded to determine the potencies of each of the designed hybrid compounds **1–6** against LSD1, as well as the enzymatically related off-targets LSD2, and MAOs A and B. These hybrid compounds **1–6** were quite potent LSD1 histone demethylase inhibitors (Table 1), comparable to the parent phenelzine and tranylcypromine analogs from which they were derived. Each displayed time-dependent LSD1 inhibition suggesting mechanism-based inactivation with *k*_inact_/*K*_i(inact)_ values that were within 3-fold of the parent mono-targeted LSD1 blockers. These compounds also showed moderate to strong selectivity against MAO A, MAO B, and LSD2 (Table 1, Supplementary Table 2, Supplementary Figs. 3–13). Hybrid compounds **1–6** were also tested against HDAC1 and the hybrid analogs **1–4** and **6** showed submicromolar potency whereas analog **5** was weaker, consistent with known structure-activity relationships related to linker length (Table 1, Supplementary Fig. 14)^15^. Pan-HDAC assays (HDACs 1–9) revealed that the benzamide compounds **1** and **2**, analogs of class I HDAC inhibitor MS-275, were > 100-fold selective for HDACs 1–3 as desired (Supplementary Fig. 15)^16, 17^.

**Table 1:** Summary of inhibitor properties toward isolated LSD1, isolated HDAC1, and MAO A

	isol. LSD1			MAO A	isol. HDAC1
Cmpd ID	*k*_inact_ (min^−1^)	*K*_i(inact)_ (µM)	*k*_inact_/*K*_i(inact)_ (min^−1^ µM^-1^)	*k*_inact_/*K*_i(inact)_ (min^−1^ µM^−1^)	IC_50_ (µM)
**1**	0.28 ± 0.05	0.51 ± 0.16	0.55	0.055 ± 0.001	0.158 ± 0.003
**2 (corin)**	0.17 ± 0.03	0.10 ± 0.06	1.70	0.074	0.147 ± 0.007
**3**	0.20 ± 0.04	0.19 ± 0.06	1.05	~0.06	0.156 ± 0.013
**4**	0.25 ± 0.06	0.39 ± 0.19	0.64	0.063 ± 0.003	0.090 ± 0.002
**5**	0.25 ± 0.07	0.27 ± 0.18	0.93	0.062 ± 0.004	1.34 ± 0.36
**6**	ND	ND	~5	0.22	0.099 ± 0.003
**bizine**^a^	0.20 ± 0.05	0.15 ± 0.09	1.33	0.11 ± 0.11	> 30
**GSK2879552**^b^	0.28 ± 0.05	0.37 ± 0.19	0.76	ND	–
**7**	0.17 ± 0.01	0.10 ± 0.02	1.70	0.37	> 30
**8**	ND	ND	~5	0.24	> 30
**9**	ND	> 20	ND	ND	0.254 ± 0.014
**MS-275**	ND	> 20	ND	ND	0.233 ± 0.026
**LBH589**	ND	> 20	ND	ND	~0.003
**SAHA**	ND	> 20	ND	ND	0.140 ± 0.013

LSD1 = 110 nM, substrate = 300 µM dimethyl histone H3K4_1–21_ peptide; HDAC1 = 2.86 nM, substrate = 20 µM acetylated P53_379–382_ tetrapeptide RHKK(Ac); MAO A = 200 nM, substrate = 200 µM tyramine

^a^MAO A inhibition data for bizine was reproduced from ref. 7

^b^Dash marks indicate that compound was not evaluated in the corresponding assay. Data (mean ± SEM) are representative of at least two independent experiments

### Corin shows sustained inhibition of the core CoREST complex

**Fig. 2:**
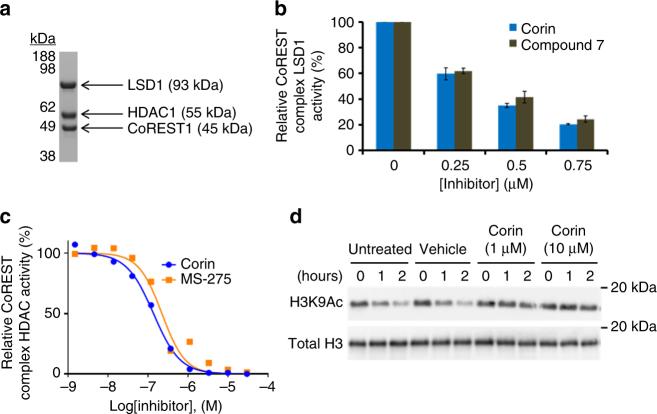
Dual inhibitors exhibit unique activity against the CoREST complex. **a** Coomassie stained gel depicting the three components of the CoREST ternary complex after purification by size exclusion chromatography. **b** Dose response produced by inhibition of LSD1 as part of the CoREST complex by corin and structurally matched compound **7**. **c** Inhibition curves generated for corin and MS-275 against HDAC1 as part of the CoREST complex. **d** Corin inhibited the deacetylation of reconstituted nucleosomes by the CoREST complex as determined by Western blot (CoREST complex = 100 nM, nucleosome = 100 nM). Data (mean ± SEM) are representative of at least two independent experiments

As CoREST complexes contain HDAC1 or 2, and compound **2** lacks the metabolic liability of the hydrazine substituent^18^ found in compound **1**, we further examined the inhibitory properties of the selective dual inhibitor **2**, hereafter called corin, with the purified core CoREST complex, prepared recombinantly from a mammalian cell expression system (Table 2)^19, 20^. Size exclusion chromatography revealed stable association of the ternary protein complex and demonstrated 1:1:1 HDAC1:LSD1:CoREST1 stoichiometry (Fig. 2a, Supplementary Fig. 16). Kinetic analysis of the reconstituted CoREST complex HDAC1 component showed that activity was linear with time and has *k*_cat_/*K*_m_ values about 4-fold greater than commercial isolated HDAC1 using the well-established fluorescent peptide HDAC assay (Supplementary Fig. 17a–c). For reasons that are not yet clear, the LSD1 activity was observed to be bi-phasic, with an initial linear phase of 3–5 min (Supplementary Fig. 17d), and we consequently focused on this first phase for inhibitor analysis. Under our experimental conditions, corin and MS-275 were similary potent in blocking CoREST complex HDAC1 activity, and corin matched the potency of the tranylcypromine analog **7** and the clinical candidate GSK2879552 toward CoREST LSD1 demethylase activity(Fig. 2b, c). Furthermore, corin was able to inhibit the deacetylation of semisynthetic, reconstituted nucleosomes by the CoREST ternary complex (Fig. 2d).

**Fig. 3:**
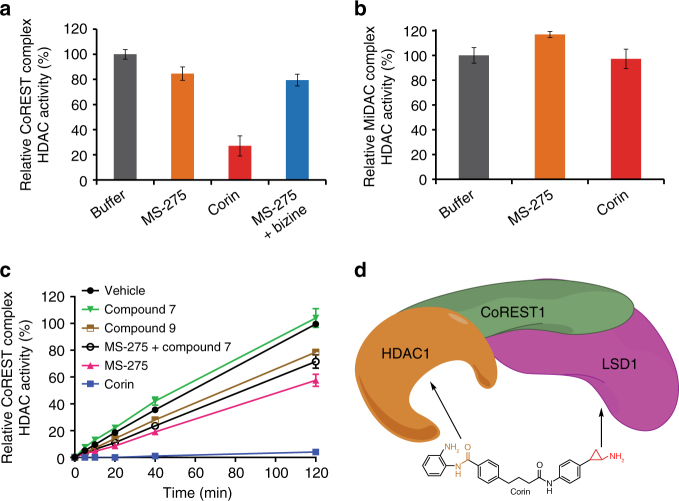
Corin exhibits sustained inhibition of CoREST complex HDAC activity. **a** Relative CoREST complex HDAC activity. **b** Relative MiDAC complex HDAC activity. Protein complexes at 500 nM and all compounds at 5 µM preincubated for 30 min prior to dialysis and dilution to measure HDAC activity at 2 nM (CoREST complex) or 8 nM (MiDAC complex). 50 µM acetylated P53_379–382_ tetrapeptide (RHKK(Ac)) was used as substrate. **c** Corin exhibits near irreversible inhibition of CoREST complex HDAC activity relative to unifunctional LSD1 and HDAC inhibitors after jump dilution (CoREST complex = 0.5 µM, inhibitor = 5 µM, substrate = 200 µM acetylated P53_379–382_ tetrapeptide RHKK(Ac)). **d** Model depicting dual engagement mechanism of corin leading to sustained inhibition of CoREST complex HDAC activity. Data (mean ± SEM) are representative of at least two independent experiments

To address the possibility that corin, because of its dual warheads, might show sustained HDAC inhibition of the CoREST complex compared to MS-275, we compared the HDAC inhibitory properties of corin to that of MS-275 after prolonged dialysis (Fig. 3a). We observed that 6 h after a 30 min exposure to corin, the HDAC1 activity of the CoREST complex was markedly reduced. In contrast, treatment of the CoREST complex with MS-275 or MS-275 plus bizine led to little loss in HDAC1 activity. We also carried out a parallel dialysis experiment after treatment with corin and MS-275 of the HDAC1-containing MiDAC complex (MIDEAS/DNTTIP1/HDAC1)^4^ that lacks an LSD1 subunit (Fig. 3b). Both MS-275 and corin lacked inhibitory effects after dialysis. These results suggest that corin’s sustained inhibition of CoREST complex HDAC activity may be related to its LSD1 interaction. To further investigate the mechanism of inhibition of the CoREST complex by corin, we performed a jump dilution experiment (Fig. 3c). In this assay, after a 30 min exposure of 0.5 µM CoREST complex to 5 µM compound (corin, MS-275, LSD1 inhibitor **7**, or desamino-corin **9**), the mixtures were diluted 200-fold and aliquots were removed at time points from 0 to 120 min for measurement of HDAC1 activity. The jump dilution assays confirmed that corin showed near irreversible inhibition of HDAC1 activity whereas the HDAC1 activity with MS-275, compound **7**, compound **9**, or a mixture of **7** and MS-275 was restored within 40% of the vehicle rate by the end of the 2 h period. The different inhibitory kinetics between compound **9** and corin are particularly notable since their chemical structures are identical except for compound **9**’s deletion of a nitrogen atom which disables its LSD1 warhead.

These results are consistent with a mechanism of dual engagement of the CoREST complex HDAC1 and LSD1 active sites by corin’s warheads that can confer long-lasting enzyme inhibition (Fig. 3d). The initial phase of inhibition in these kinetic studies (Table 2) do not support enhanced potency of corin over the mono-functional inhibitors, so it is unlikely that the active sites of LSD1 and HDAC1 in the CoREST complex support dual occupancy by corin in the baseline state. Interactions of one enzyme active site with one warhead of corin may lead to a high effective concentration of the second warhead for the other enzyme subunit which over time induces conformational changes in the protein complex facilitating concurrent engagement. Computational modeling of the subunit proteins suggests the plausibility of energetically stable conformations with surface loop changes that allow for dual occupancy of LSD1 and HDAC1 by corin, but future studies will be needed to understand the precise structural mechanisms behind corin’s inhibition of the CoREST complex.

**Table 2:** Biochemical characterization of dual inhibitors against LSD1 and HDAC1 as part of the CoREST ternary complex

Cmpd ID	CoREST complex (LSD1) IC_50_ (µM)	CoREST complex (HDAC1) IC_50_ (µM)
**1**	1.8 ± 0.3	0.230 ± 0.014
**2 (corin)**	0.33 ± 0.05	0.206 ± 0.035
**bizine**	1.4 ± 0.6	ND
**GSK2879552**^a^	0.38 ± 0.01	–
**7**	0.36 ± 0.01	ND
**9**	> 20	0.328 ± 0.026

LSD1 assay: CoREST complex = 100 nM, substrate = 60 µM dimethyl histone H3K4_1–21_ peptide; HDAC1 assay: CoREST complex = 2 nM, substrate = 50 µM acetylated P53_379–382_ tetrapeptide RHKK(Ac)

^a^Dash marks indicate that compound was not evaluated in the corresponding assay. Data (mean ± SEM) are representative of at least two independent experiments

### Anti-tumor properties of corin

**Fig. 4:**
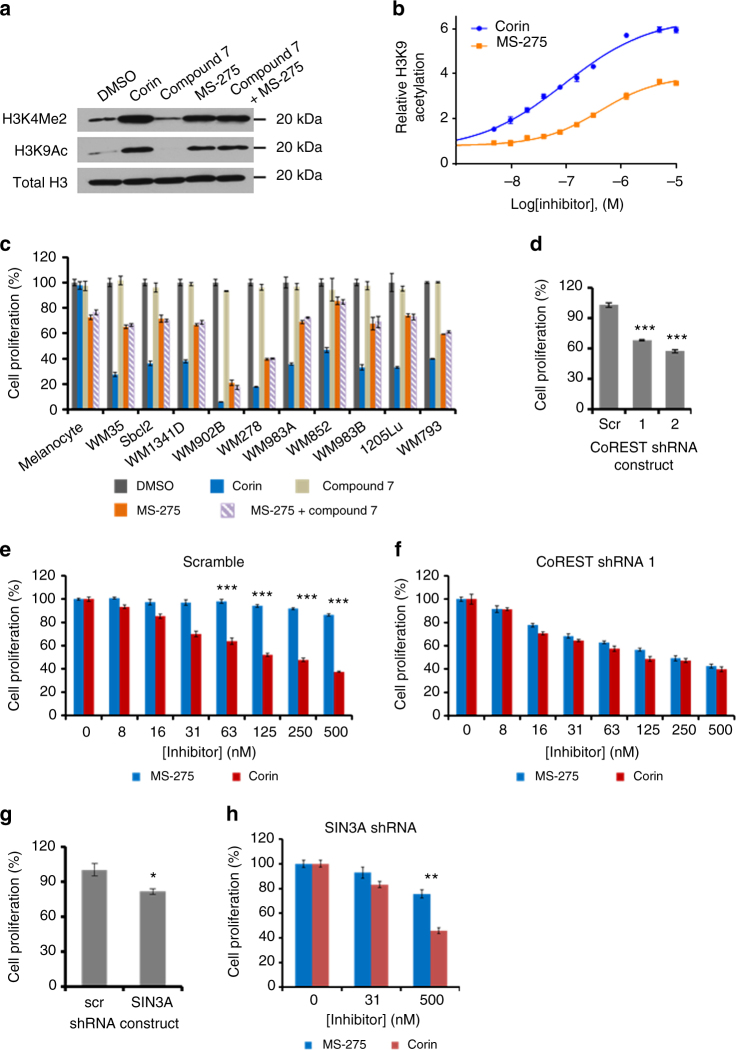
Pharmacologic effects of corin and related compounds on melanoma cells and the role of CoREST. **a** Western blot depicting increases in histone H3K4 methylation and H3K9 acetylation in WM983B melanoma cells induced by 10 µM inhibitor treatment. **b** Corin (EC_50_ = 0.095 ± 0.017 µM) was more potent than MS-275 (EC_50_ = 0.42 ± 0.07 µM) and more efficacious at inducing histone H3K9 acetylation in WM983B melanoma cells as determined by ELISA after 24 h treatment (*n* = 3). **c** Treatment with corin (1 µM) for 72 h potently inhibited cell growth across a panel of ten melanoma cell lines without significantly affecting primary melanocytes whereas MS-275 (1 µM) was similarly potent toward transformed and non-transformed cells (*n* = 3). **d** Knockdown of CoREST1 inhibits WM983B melanoma cell proliferation (*n* = 3). **e**, **f** Knockdown of CoREST1 enhances the potency of MS-275 but does not affect the potency of corin as determined after 72 h treatment (*n* = 3). **g**, **h** Knockdown of SIN3A inhibits WM983B melanoma cell proliferation but does not sensitize cells to the antiproliferative effects of MS-275 after 72 h treatment (*n* = 3). Note that cell proliferation was determined using the PicoGreen^®^ cell proliferation assay and data were normalized to zero inhibitor concentration for individual cell lines (scrambled shRNA and CoREST shRNA1). Data (mean ± SEM) are representative of at least two independent experiments (unpaired *t* test, *p < 0.05, **p < 0.01, ***p < 0.001)

Corin was subjected to an NCI60 screen^21, 22^ which demonstrated preferential growth inhibition of melanoma cells vs. other cancer cell lines, consistent with the known epigenetic influences in the development and progression of melanoma (Supplementary Fig. 18)^23, 24^. We subsequently tested corin’s effects on malignant melanoma WM983B histone modifications. Western blot and ELISA revealed that corin stimulated global H3K9 acetylation (H3K9Ac), as well as H3K4 mono- (H3K4Me1), di- (H3K4Me2), and tri-methylation (H3K4Me3) (Fig. 4a, b, Supplementary Fig. 19). In comparison to MS-275, corin appeared to more potently (corin EC_50_ 95 nM vs. MS-275 EC_50_ 420 nM) and efficaciously induce cellular H3K9 acetylation. Although MS-275 lacks direct LSD1 inhibitory properties, its ability and that of other HDAC inhibitors to influence global H3K4 methylation has been noted previously, presumably resulting from indirect impact on cellular methyltransferase or demethylase enzymes^25, 26^.

Notably, corin showed more powerful anti-proliferative action against WM983B cells relative to MS-275 with an IC_50_ of ∼200 nM, about 12-fold lower than that of MS-275 (Fig. 4e and Supplementary Fig. 20a, e). In fact, corin (1 µM) consistently showed a greater anti-proliferative effect than MS-275 (1 µM) across a panel of 10 melanoma cell lines (Fig. 4c). We also observed that combining the monofunctional LSD1 inhibitor **7** (1 µM) along with MS-275 (1 µM) across these cell lines did not match the anti-proliferative effect of corin (1 µM) suggesting that the integration of the two inhibitory warheads in corin is critical for its pharmacologic action. Consistent with this idea, desamino-corin compound **9** with its LSD1 warhead disrupted has a less potent IC_50_ of 5 µM, similar to that of MS-275 (Supplementary Fig. 20b). Interestingly, corin (1 µM) was non-toxic to primary human melanocytes in contrast to MS-275 (1 µM) (Fig. 4c).

To explore the possible role of the CoREST complex in mediating the more powerful anti-proliferative action of corin vs. MS-275 against WM983B cells, we used shRNA to stably knockdown CoREST1, confirmed by qRT-PCR and Western blot (Supplementary Fig. 20c, d). It was also observed that these CoREST1 knockdown WM983B cells grew about 40% more slowly relative to control WM983B cells, indicating the functional importance of CoREST1 in WM983B cell proliferation (Fig. 4d). We next performed a dose–response analysis of MS-275 and corin in the parental vs. the CoREST1 knockdown WM983B cells. Strikingly, knockdown of CoREST1 conferred a heightened sensitivity to the anti-proliferative action of MS-275 in WM983B cells, rendering a similar dose–response profile to that of corin, whose antiproliferative effects were not altered by CoREST1 knockdown (Fig. 4e, f, Supplementary Fig. 20e, f). In contrast, knockdown in WM983B cells of the distinct corepressor protein SIN3A which participates in an HDAC1 complex that does not include LSD1^5^ does not sensitize cells to MS-275 and the selectivity margin between corin and MS-275 is maintained under these conditions (Fig. 4g, h, Supplementary Fig. 21a).

**Fig. 5:**
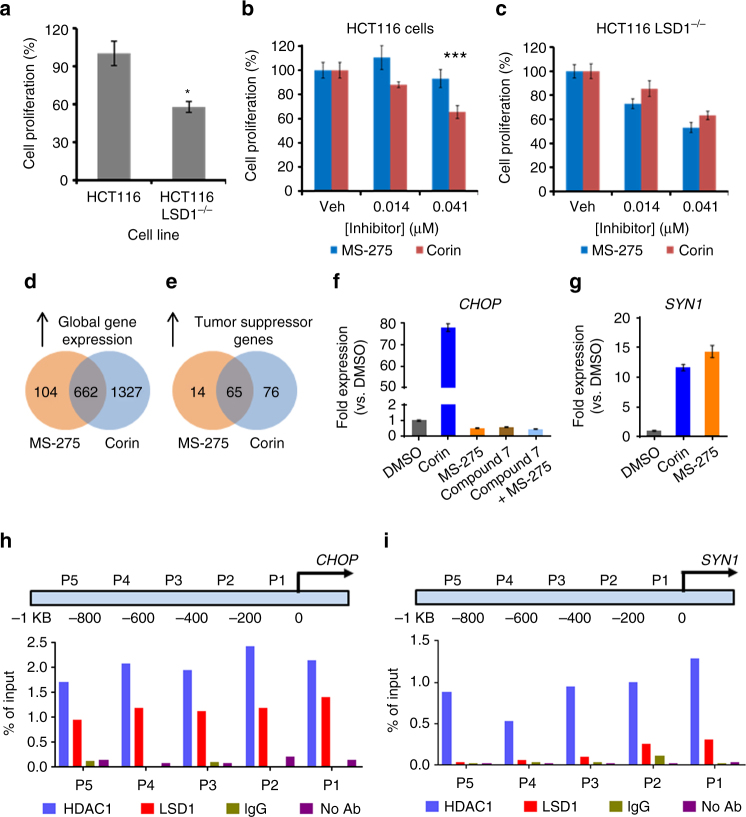
Analysis of gene knockout effects in HCT116 cells and gene expression changes in melanoma associated with corin treatment. **a** Knockout of LSD1 in HCT116 cells decreased the rate of cancer cell proliferation by ~40% (*n* = 4). **b**, **c** Knockout of LSD1 enhanced the potency of MS-275 but did not affect the potency of corin in HCT116 cells as determined by [^3^H]thymidine incorporation after 48 h treatment (*n* = 4). **d**, **e** Venn diagrams depicting increases in global and tumor suppressor gene transcription (fold change ≥ 2σ, *p* < 0.05), respectively, upon 2.5 µM MS-275 or corin treatment (*n* = 3). **f**, **g** qRT-PCR validation of gene expression changes identified by microarray. WM983B melanoma cells were treated with inhibitor (2.5 µM) for 24 h prior to RNA isolation and analysis (*n* = 3). **h** ChIP-PCR localized CoREST complex components HDAC1 and LSD1 to the promoter region of *CHOP* (*n* = 3). **i** ChIP-PCR localized HDAC1, but not LSD1, to the promoter region of *SYN1* (*n* = 3). Data (mean ± SEM) are representative of at least three independent experiments (unpaired *t* test, *p < 0.05, ***p < 0.001)

We also investigated the comparative pharmacology of MS-275 and corin in HCT116 colon cancer cells and the isogenic LSD1^−/−^ HCT116 cell line^27^. As observed previously, LSD1^−/−^ HCT116 cells show a diminished proliferation rate relative to the parental HCT116 cells (Fig. 5a, Supplementary Fig. 21b). Loss of LSD1 enhanced the sensitivity of HCT116 cells to MS-275, but not corin (Fig. 5b, c). Taken together with the CoREST1 and SIN3A knockdown experiments, these results suggest that the more powerful anti-proliferative action of corin vs. MS-275 in the melanoma and colon cancer cells analyzed may involve its more comprehensive targeting and sustained inhibition of the LSD1-containing CoREST complex relative to other complexes. Nevertheless, even with CoREST1 knockdown, corin retains potency suggesting it can also block class I HDACs in other contexts.

To further assess the mechanistic basis of corin vs. MS-275 in WM983B cells, we analyzed cellular gene expression profiles after 24 h treatment with 2.5 µM of each compound. Approximately 1300 genes were selectively up-regulated at least 2-fold by corin compared with MS-275 under these conditions (Fig. 5d, Supplementary Fig. 22a). Gene ontology analysis suggested that genes important for differentiation, as well as modulators of cell motility were significantly up-regulated by corin compared to MS-275 (Supplementary Table 3). In addition, a large number of tumor suppressor genes were preferentially induced by corin, many of which have been previously observed to be epigenetically silenced in cancer (Fig. 5e, Supplementary Tables 4 and 5). We examined several of these tumor suppressors by qRT-PCR including *p21*, *CHOP*, *SIK1*, *MXD1* and several others, each of which showed dramatic increases after corin treatment vs. MS-275 treatment, confirming the microarray data (Fig. 5f, g, Supplementary Fig. 22b–m). As in the anti-proliferative findings, none of these genes showed enhancement by combining MS-275 with compound **7**, highlighting the pharmacologic advantage of the hybrid molecule over the separate unifunctional compounds. We also found that the preferential induction by corin vs. MS-275 of *CHOP* and *MXD1* was abolished in the CoREST knockdown melanoma cells (Supplementary Fig. 23a, b). Using ChIP-PCR analysis, we observed that the promoter regions of the *CHOP* and *MXD1* genes showed high occupancy by both LSD1 and HDAC1 compared with control genes *SYN1* and *VAMP7* which were not selectively induced by corin (Fig. 5h, i, Supplementary Figs. 22n, 23c, d, 24a, b). These data suggest that corin’s dual targeting of LSD1 and HDAC1 in the CoREST complex may contribute to its enhanced cellular pharmacology in melanoma.

The effects of LSD1 and HDAC inhibitors as well as corin were also investigated with the primary human cutaneous squamous cell carcinoma cell lines IC1 and MET1 (Table 3 and Supplementary Figs. 25–27). In contrast to the melanoma lines examined, we found that both of these cutaneous cancer lines were quite sensitive to the potent monofunctional LSD1 inhibitors compound **7**, GSK2879552, and to a lesser extent tranylcypromine (Table 3, Supplementary Figs. 25 and 26) but not to the MAO inhibitor pargyline which is devoid of LSD1 inhibitor activity^28^. We also found that the proliferation of IC1 and MET1 were more powerfully inhibited by corin than either the LSD1 inhibitors or the mono-functional HDAC inhibitors MS-275 and compound **9** (Table 3, Supplementary Figs. 25 and 26). In the case of IC1 and MET1, the anti-proliferative effects of the mono-functional LSD1 inhibitors compound **7** and GSK2879552 were partially additive with MS-275 when used in combination. But the IC_50_ of corin was still 5-fold and 2-fold lower in IC1 and MET1, respectively, than the combination of compound **7** and MS-275. Moreover, corin proved far less toxic to primary human keratinocytes than MS-275, which were rather resistant to LSD1 inhibitors (Supplementary Fig. 28). These data highlight the potential therapeutic advantage of the hybrid agent in cancer cells that are particularly LSD1 inhibitor sensitive.

**Table 3:** Anti-proliferative effects of corin and related compounds in cutaneous squamous cell carcinoma cells after 72 h treatment

	IC_50_ ± SE (µM)	
Compound	IC1 cells	MET1 cells
**Corin**	0.041 ± 0.011	0.006 ± 0.001
**MS-275**	1.03 ± 0.07	0.144 ± 0.014
**Compound 9**	0.397 ± 0.042	0.094 ± 0.009
**Compound 7**	0.448 ± 0.187	0.171 ± 0.036
**GSK2879552**	2.91 ± 0.71	0.211 ± 0.042
**MS-275 + Compound 7**	0.249 ± 0.075	0.011 ± 0.001
**MS-275 + GSK2879552**	0.275 ± 0.027	0.042 ± 0.007
**Tranylcypromine**	3.94 ± 0.98	2.03 ± 0.54
**Pargyline**	> 270	> 270

### Pharmacologic analysis of corin using a mouse xenograft

**Fig. 6:**
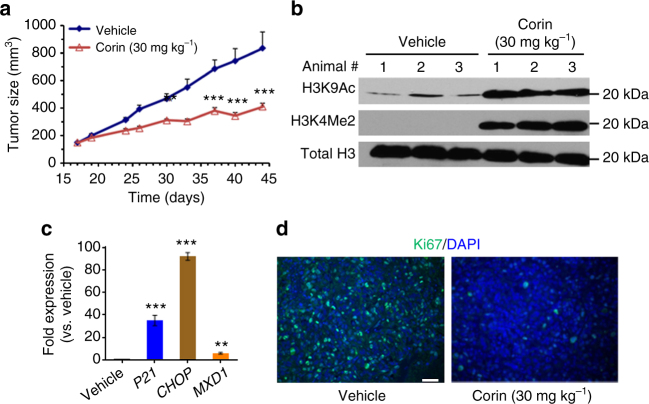
In vivo analysis of corin in a melanoma xenograft. **a** Daily IP administration of corin (30 mg kg^−1^) potently inhibited tumor growth in an SK-MEL-5 melanoma cell mouse xenograft model over the course of a 28-day treatment regimen (*n* = 10 mice per condition). **b** Western blot depicting increases in histone H3K4 methylation and H3K9 acetylation in tumor tissue obtained from the SK-MEL-5 melanoma xenograft. **c** Gene expression changes induced by corin in mouse xenograft tumor tissue as determined by qRT-PCR. **d** Corin treatment decreased the expression of Ki67, a biomarker for cell proliferation, in mouse xenograft tumor tissue (scale = 100 µm). Data (mean ± SEM) are representative of at least two independent experiments (unpaired *t* test, **p < 0.01, ***p < 0.001)

We next demonstrated that corin showed high metabolic stability in human plasma, human liver microsomes, and mouse liver microsomes (Supplementary Table 6), and was well-tolerated in mice for 10 days of once daily intraperitoneal (IP) administration of up to 30 mg kg^−1^ based on stable similar weight relative to vehicle (Supplementary Fig. 29). We went on to examine the ability of corin to impact tumor growth in a melanoma mouse xenograft model using SK-MEL-5 cells. Melanoma xenografts of SK-MEL-5 cells are well established^29^ and, importantly, corin showed comparable efficacy (IC_50_ ~130 nM) in this cell line as compared to WM983B cells (Supplementary Fig. 30). These studies showed that corin treatment (30 mg kg^−1^, IP, every 24 h) led to a 61% reduction in tumor volume after 28 days relative to vehicle (Fig. 6a), with body weights and blood cell counts being similar between the treated and control animals at the end of the study (Supplementary Fig. 31). We attempted treatment of these xenograft mice with the same dose and schedule of MS-275 but this proved too toxic as 6 of 10 animals died with 1 week so this arm was discontinued. Examination of the tumor tissue recovered from mice treated with corin vs. vehicle showed that tumor cells from the corin-treated animals displayed elevated H3K9 acetylation, H3K4 dimethylation (Fig. 6b), and increased expression of *p21*, *CHOP*, and *MXD1* (Fig. 6c), consistent with the cell culture experiments. Moreover, tumor cells from corin-treated animals showed a reduction in the proliferation biomarker Ki67 suggesting that the corin-treatment was blocking proliferation of these cells (Fig. 6d). These results substantiate corin’s promise for in vivo anti-cancer applications.

## Discussion

It is generally accepted that in the context of cancer, single agent/single target therapeutics are poorly effective because of cancer pathway redundancies and the emergence of drug resistance^30^. Thus, many anti-neoplastic treatment protocols involve two or more drugs to achieve synergistic efficacy and prevent clonal resistance^31, 32^. An alternative strategy could involve the development of one drug that has engineered multifaceted pharmacodynamics. There have been several reports both in the context of small molecules, as well as protein biologics to exploit and/or rationally incorporate multi-target pharmacology in anti-tumor agents^33–35^. Targeting two enzymes in the same complex that are thought to work concertedly to epigenetically inactivate gene expression offers an opportunity for synergistic pharmacology. While bisubstrate analog inhibitors that bind bivalently to a single enzyme that follows a ternary complex mechanism are commonplace^36^, dual action inhibitors aimed at two different enzymes in a protein complex are not well-established; however, these studies lend credence to the feasibility of this strategy. A somewhat related bivalent approach targeting LSD1 and HDAC was recently reported although the hybrid compound is designed to split into LSD1 and HDAC inhibitor components after LSD1 inactivation and the CoREST complex targeting capability has not yet been assessed^37^.

Our findings suggest that comprehensively targeting the CoREST complex can enhance residence time and this is facilitated by the hybrid functionality in corin vs. monofunctional HDAC inhibitors. However, further genomic studies will be needed to provide a full understanding of the relative targeting of the CoREST complex by corin chromatin wide in cells. Regardless, our results substantiate that such targeting can offer possible anti-tumor efficacy in malignancies that are especially sensitive to LSD1 inhibitors such as cutaneous squamous cell cancer, as well as those that are resistant to such agents, as seen in melanoma. Beyond the potential value of comprehensive epigenetic complex targeting and enhanced residence time^38^, dual action inhibitors promote a balance in concomitant enzyme blockade at the single cell level and overcome the substantial regulatory challenge of advancing two separate compounds concurrently into clinical trials.

## Methods

### LSD1 expression and inhibition assay

LSD1 inhibition was determined using a previously developed horseradish peroxidase (HRP) coupled assay. Briefly, LSD1 (residues 171–852) containing an N-terminal GST-tag was overexpressed in *E. coli* and purified via affinity chromatography^39^. GST-LSD1 concentration was determined by gel electrophoresis (~0.9 mg ml^−1^, ~8.8 µM) and aliquots were stored at −80 °C (storage buffer: 280 mM NaCl, 5.4 mM KCl, 20 mM Na_2_HPO_4_, 3.6 mM KH_2_PO_4_, 1 mM BME, 1 mM EDTA, 10% glycerol, pH 7.4). For each experiment, a fresh aliquot was thawed and diluted with storage buffer (not containing BME) to 0.45 mg ml^−1^ (4.4 µM) immediately before use. 60 µl reactions containing 50 mM HEPES (pH 7.5), 0.1 mM 4-aminoantipyrine (4-AP), 1 mM 3,5-dichloro-6-hydroxybenzensulfonate (DHBS), 300 µM diMeK4H3_1–21_ substrate (ARTK(Me)_2_QTARKSTGGKAPRKQLA, *K*_m_ = 25 µM, *k*_cat_ = 3 min^−1^)^39^, 0.04 mg ml^−1^ (906 nM) HRP (Worthington Biochemical Corporation, LS002559), and varying concentrations of inhibitor were initiated by the addition of LSD1 (final [LSD1] = 110 nM). Changes in absorbance at 515 nm were monitored over 20 min at 25 °C using a Beckman Instruments DU series 600 spectrophotometer. Product formation was calculated using the Beer–Lambert law and an extinction coefficient of 26,000 M^−1^ cm^−1^ for the generated chromophore. Progress curves representing the concentration of product formed over time were plotted and subjected to a series of mathematical transformations to determine the kinetic parameters (*k*_inact_, *K*_i(inact)_) for each compound^7, 39^. Inhibitor stocks were prepared in 8% DMSO/water by 2-fold serial dilution (final concentration of DMSO in each reaction was 2%). Reported kinetic parameters represent at least two independent experiments.

### CoREST complex production and purification

Constructs of LSD1, HDAC1, and FLAG-tagged CoREST1 were each cloned into a pcDNA 3.0-based vector and transiently transfected into HEK293F suspension cells (Invitrogen) using branched polyethylenimine (Sigma-Aldrich) and incubated for 48 h at 37 °C^19, 20,]^. Next, cells were pelleted by centrifugation at 2860*xg* for 10 min and resuspended in cold lysis buffer (50 mM Tris–HCl, pH 7.5, 50 mM KCl, 5% glycerol, 0.4% Triton X-100, 1X Roche EDTA-free Complete Protease Inhibitor cocktail). The cells were lysed via sonication (15 s on, 30 s off, 42% amplitude, 3 cycles) and insoluble fractions were pelleted by centrifugation at 26,300x*g* for 20 min. The complex was initially purified via FLAG affinity chromatography by incubating the lysate with 1 ml of Anti-FLAG M2 affinity gel (Sigma-Aldrich) per liter of cell culture at 4 °C for 30 min. The resin was collected and washed with wash buffer (50 mM Tris–HCl, pH 7.5, 50 mM KCl, 5% glycerol) three times and then washed with cleavage buffer containing reducing agent (50 mM Tris–HCl, pH 7.5, 50 mM KCl, 5% glycerol, 0.5 mM tris(2-carboxyethyl)phosphine (TCEP)) three times to prepare for TEV protease cleavage. The affinity gel was resuspended in cleavage buffer and incubated with TEV protease (Invitrogen) overnight at 4 °C followed by concentration of the cleaved complex. The complex was subsequently purified using Size Exclusion Chromatography by loading onto a 25 ml Superose 6 10/300 GL column (GE Healthcare) pre-equilibrated with buffer (50 mM Tris–HCl, pH 7.5, 50 mM KCl, 0.5 mM TCEP) and run at 0.25 ml min^−1^. Fractions were checked for purity via SDS–PAGE and concentrated. Complex concentration was determined via MicroBCA assay (ThermoScientific) with BSA as the standard. The final complex was then stored at 4 °C. The complex was expressed in 1.2 L of culture and produced ~0.25–0.5 mg of > 90% pure protein^19^. Complex is stable for ~2 weeks at 4 °C, do not freeze.

### MiDAC ternary complex production and purification

The MiDAC complex (MIDEAS, DNTTIP1, HDAC1) was expressed in HEK293F cells which were lysed in 50 mM Tris–HCl, 100 mM potassium acetate, 10% (v/v) glycerol, 0.5% (v/v) Triton X-100, pH 7.5 in the presence of 1× Roche Complete EDTA-free protease inhibitor^4^. The complex was purified by FLAG-affinity chromatography and, after washing, was eluted by TEV-protease cleavage and finally purified by gel filtration on a Superdex-200 column (GE Healthcare)^4^.

### CoREST complex (LSD1) inhibition assay

Inhibition of CoREST complex LSD1 activity was determined using the same coupled assay described for isolated LSD1 with the following modifications. The CoREST ternary complex (120 nM) was incubated with varying inhibitor concentrations in the presence of 60 mM HEPES (pH 7.4), 0.12 mg ml^−1^ BSA, and 0.12 mM inositol hexakisphosphate for 5 min at 25 °C as a 50 µl reaction. After a 5 min preincubation, the coupling reagents 4-AP, DHBS, and HRP were added and the reaction was initiated by the addition of substrate. The final reaction volume was 60 µl and comprises 50 mM HEPES (pH 7.4), 0.1 mg ml^−1^ BSA, 0.1 mM inositol hexakisphosphate, 0.1 mM 4-AP, 1 mM DHBS, 60 µM diMeK4H3_1–21_ substrate, 0.04 mg ml^−1^ (906 nM) HRP, 100 nM CoREST ternary complex, and varying concentrations of inhibitor. Inhibitory parameters were determined by examining changes in absorbance over 5 min. Reported kinetic parameters represent at least two independent experiments.

### HDAC isoform inhibition assay

The inhibitory effect of compounds on HDAC1–HDAC9 function was determined in vitro using an optimized homogenous assay performed in 384-well plate format^16^. In this assay, recombinant HDAC protein (HDAC1 100 pg µl^−1^, HDAC2 200 pg µl^−1^, HDAC3 100 pg µl^−1^, HDAC4 0.5 pg µl^−1^, HDAC5 10 pg µl^−1^, HDAC6 350 pg µl^−1^, HDAC7 2 pg µl^−1^, HDAC8 16 pg µl^−1^, HDAC9 20 pg µl^−1^; BPS Bioscience) was incubated with inhibitory compound for 3 h, and then fluorophore-conjugated substrates MAZ1600 and MAZ1675 were added at a concentration equivalent to the substrate *K*_m_ (MAZ1600: 8.9 µM for HDAC1, 10.5 μM for HDAC2, 7.9 μM for HDAC3, and 9.4 μM for HDAC6. MAZ1675: 11.5 μM for HDAC4, 64.7 μM for HDAC5, 29.6 μM for HDAC7, 202.2 μM for HDAC8 and 44.3 μM for HDAC9). Reactions were performed in assay buffer (50 mM HEPES, 100 mM KCl, 0.001% (v/v) Tween 20, 0.05% (w/v) bovine serum albumin, 200 μM TCEP, pH 7.4) and followed for fluorogenic release of 7-amino-4-methylcoumarin from substrate upon deacetylase and trypsin enzymatic activity. Trypsin was present at a final concentration of 50 nM (Worthington Biochemical Corporation). Fluorescence measurements were obtained approximately every 5 min using a multilabel plate reader and plate stacker (Envision, Perkin-Elmer). Data were analyzed on a plate-by-plate basis for the linear range of fluorescence over time. The first derivative of data obtained from the plate capture corresponding to the mid-linear range was imported into analytical software (Spotfire DecisionSite and GraphPad Prism). Replicate experimental data from incubations with inhibitor were normalized to DMSO controls.

### HDAC1 inhibition assay

HDAC1 inhibitory activity was determined for each compound using the Fluorogenic HDAC1 assay kit available from BPS Bioscience (50061). Minor deviations from the manufacturer’s instructions were employed and are described below. HDAC substrate 1 (50032) was used instead of HDAC substrate 3 (50037). HDAC substrate 1 consists of a tetrapeptide based on residues 379–382 (RHKK(Ac)) of the tumor suppressor protein P53, a known HDAC substrate. The peptide contains a fluorescent reporter that is activated after deacetylation by the addition of Developer (50030). Reactions were carried out in 96-well plates (Nunc low binding, black microtiter plates) with compounds being screened in 10 dose IC_50_ format using 3-fold serial dilutions. 50 µl reactions containing HDAC assay buffer (50031), 0.1 mg ml^−1^ BSA, 20 µM HDAC substrate 1, and varying concentrations of inhibitor were initiated by the addition of HDAC1 (final [HDAC1] = 2.86 nM) and incubated at 37 °C for 30 min. After incubation, the reactions were quenched by the addition of Developer and incubated at room temperature for 15 min. Fluorescence was determined using a BioTek Instruments Synergy HT plate reader with excitation at 360 nm and emission at 460 nm. Inhibitor stocks were prepared in 20% DMSO/water by 3-fold serial dilution (final concentration of DMSO in each reaction was 2%). Control experiments were run to account for intrinsic compound fluorescence/fluorescence suppression. Fluorescence was normalized to the vehicle control, converted to percent activity, and plotted against inhibitor concentration to generate 10-dose IC_50_ curves. To account for slow, tight-binding inhibitors, all components of the reaction except for the substrate were mixed in the 96-well plate and incubated at room temperature for 10 min. Then, the reaction was initiated by the addition of substrate and fluorescence was monitored as previously described. Data are representative of at least three independent experiments.

### Protein complex HDAC inhibition assay

Inhibition of CoREST complex HDAC activity was determined using the same assay kit described in the HDAC1 inhibition assay with minor differences. Reactions were carried out for 10 min using 50 µM HDAC substrate 1 and a final concentration of 2 nM CoREST ternary complex or 8 nM MiDAC ternary complex. Slow, tight-binding was also accounted for as described for the HDAC1 inhibition assay. Data are representative of at least three independent experiments.

### CoREST complex (HDAC) jump dilution assay

The CoREST ternary complex (0.5 µM, buffer: 25 mM Tris-HCl, 50 mM KCl, 0.5 mM TCEP, pH 7.5) was incubated with the indicated inhibitor (5 µM, 2% DMSO, 100 µM inositol hexakisphosphate) at 25 °C for 30 min prior to jump dilution^40^. The complex was then diluted to a final concentration of 2 nM in HDAC assay buffer (BPS Bioscience, 50031) containing 200 µM HDAC substrate 1 (BPS Bioscience, 50032) and 0.1 mg ml^−1^ BSA. 50 µl aliquots were quenched at 0, 5, 10, 20, 40, 120 min with an equal volume of Developer (BPS Bioscience, 50030) and fluorescence was measured as described in the HDAC1 inhibition assay.

### Prolonged HDAC inhibition assay

To determine if dual inhibitors could maintain inhibition of protein complex HDAC activity after extensive dialysis, 0.5 µM CoREST or MiDAC ternary complex was treated with 5 µM inhibitor for 30 min at 4 °C in 50 µl of 25 mM Tris–HCl, 50 mM KCl, 100 µM inositol hexakisphosphate, and 0.5 mM TCEP, pH 7.5. After 30 min, the reactions were transferred to dialysis cassettes (Slide-A-Lyzer^®^ 10 K MWCO MINI Dialysis Device, Thermo Fisher Scientific, Halethorpe, MD) and dialyzed against 1 L of buffer (25 mM Tris–HCl, 50 mM KCl, 0.5 mM TCEP, pH 7.4) three times for 2 h each time at 4 °C. Of note, it is important to keep the sample volume level at the same level of the dialysate to avoid reaction dilution. After 6 h dialysis, the reactions were transferred to 0.5 ml Eppendorf tubes and the protein complex HDAC inhibition assay was carried out with the dialyzed complex. Inhibitor stocks were prepared, as described for the HDAC1 inhibition assay. Data are representative of at least two independent experiments.

### Nucleosome deacetylation assay

N-terminal histone H3 tail peptides containing H3K9Ac modifications were prepared using SPPS (0.1 mmol) and ligated to truncated, recombinant histone H3 via sortase ligation^41^. Semisynthetic histone H3 was refolded with other core histones to form histone octamers followed by nucleosome reconstitution via gradient dialysis in the presence of nucleosomal DNA^42, 43^. The CoREST ternary complex (final [CoREST] = 100 nM) was pretreated with inhibitor at 25 °C for 30 min in 50 mM HEPES, pH 7.5, 100 mM KCl and 0.2 mg ml^−1^ BSA. Reactions were initiated by the addition of nucleosome substrate (final [nucleosome] = 100 nM) with a final reaction volume of 50 µl. 10 µl aliquots taken at 0 h, 1 h, and 2 h were quenched with 10 mM EDTA and 4x sample loading buffer, resolved using 15% SDS–PAGE gels, and analyzed via Western blot. Nitrocellulose membranes were blocked with 5% BSA in TBST and incubated overnight with primary antibodies, either α-H3K9Ac (Abcam, ab4441, 1:5000) or α-Total H3 (Abcam, ab1791, 1:10,000), at 4 °C. Membranes were then treated with HRP-conjugated secondary antibody which was detected using ECL reagent available from GE Healthcare. Data are representative of two independent experiments.

### LSD2 counterscreen

C-terminally His tagged lysine specific demethylase 2 (LSD2) was overexpressed in *E. coli* after which the cells were lysed using a French press with cold buffer (280 mM NaCl, 5.4 mM KCl, 20 mM Na_2_HPO_4_, 3.6 mM KH_2_PO_4_, 1.3 mM PMSF, 6.8 µg µl^−1^ DNase I, 10% (v/v) glycerol, pH 7.4, 1× Roche Complete EDTA-free protease inhibitor). Lysate was clarified by centrifugation and the target protein purified by nickel affinity chromatography^7^. Inhibitor potency toward the LSD1 homolog, LSD2, was determined using a procedure similar to that described for LSD1 above^7, 28, 39^. Briefly, 60 µl reactions containing 50 mM HEPES, pH 7.5, 0.1 mM 4-AP, 1 mM DHBS, 100 µM diMeK4H3_1−21_ substrate, 0.04 mg ml^−1^ (906 nM) HRP, and 20 µM inhibitor were initiated by the addition of LSD2 (final [LSD2] = 430 nM). Absorbance measurements and data processing were carried out as described for LSD1. Data are representative of at least two separate experiments.

### MAO A/B counterscreen

MAO A (M7316), MAO B (M7441), and the tyramine substrate (T90344) were purchased from Sigma-Aldrich. MAO A (83.2 µM) and MAO B (83.7 µM) were aliquoted and stored at −80 °C until use according to the manufacturer’s instructions. MAO A was diluted 8-fold to 10.4 µM with 50 mM HEPES, pH 7.4 immediately before use. The tyramine substrate was stored at −80 °C as a 100 mM stock in DMSO and diluted to 0.8 mM (MAO A) and 0.5 mM (MAO B) with water immediately before use. In total 100 µl reactions containing 50 mM HEPES, pH 7.5, 0.1 mM 4-AP, 1 mM DHBS, tyramine substrate ([tyramine] = 200 µM for MAO A (*K*_m_ = 29 µM, *k*_cat_ = 93 min^−1^) and 125 µM for MAO B (*K*_m_ = 93 µM, *k*_cat_ = 0.20 min^−1^) assays), 0.04 mg ml^−1^ (906 nM) HRP, and varying concentrations of inhibitor were initiated by the addition of either MAO A or B (final [MAO A] = 0.2 µM, final [MAO B] = 1.674 µM)^7, 28^. Inhibitor stocks were prepared as before and absorbance measurements and data processing were carried out as described for LSD1. Data reported represent at least two separate experiments.

### Cell Culture

Ten melanoma cell lines were obtained from Dr. Meenard Herlyn (The Wistar Institute, Philadelphia, PA). SK-MEL-5 cells were obtained from Dr. Levi A. Garraway (Dana-Farber Cancer Institute, Boston, MA). HPMs were purchased from Life Technologies (Grand Island, NY). HCT116 and LSD1^−/−^ HCT116 cells were obtained as a gift from Dr. Robert Casero Jr^27^ and LSD1 knockout was confirmed by western blot (α-LSD1, Abcam, ab17721, 1:500). The human cutaneous squamous cell carcinoma (cSCC) cell lines were derived from moderately differentiated primary tumors: cSCC-IC1 cells were isolated from a tumor on the right temple of a 77-year-old male immunocompetent patient and cSCC-MET1 cells were obtained from a tumor on the dorsum of the left hand of a 55-year-old male renal transplant recipient. Primary keratinocytes were purchased from ATCC (Manassas, VA). All cell lines used were routinely checked for and found to be free of mycoplasma contamination. Melanoma cell lines were cultured in Dulbecco’s modified eagle medium (Invitrogen) supplemented with 10% fetal bovine serum, l-glutamine (2 mM), and 1% penicillin/streptomycin. HPMs were cultured in Medium 254 with human melanocyte growth supplements. HCT116 cells were cultured in McCoy’s 5 A medium with l-glutamine purchased from Corning (Iwakata & Grace modification, Corning, N.Y.) and supplemented with 10% fetal bovine serum. cSCC cells were cultured in a nutrient mixture of Dulbecco’s minimal essential medium with Ham’s F12 medium (3:1) supplemented with 10% FBS, hydrocortisone, human insulin, mouse EGF, cholera toxin, apo-transferrin, and lyothyronine (L4). Keratinocytes were cultured in Dermal Cell Basal Medium and supplemented with bovine pituitary extract (0.4%), TGF-α (0.5 ng ml^−1^), l-glutamine (6 mM), hydrocortisone (100 ng ml^−1^), insulin (5 mg ml^−1^), epinephrine (1 mM), and apo-transferrin (5 mg ml^−1^) following guidelines from ATCC. All cell lines were maintained in a 37 °C incubator at 5% CO_2_.

### Cell treatment with compounds

Inhibitor stocks were prepared in DMSO and diluted as necessary. Appropriate stock solutions were added to culture medium to achieve the desired final concentrations. An equal amount of DMSO was used as a vehicle control.

### Western blot

Whole-cell lysate was prepared in 3D-RIPA buffer. Protein (20 µg) was separated by 12% SDS–PAGE and transferred to a polyvinylidene difluoride membrane. Membranes were blocked using 5% nonfat dry milk in PBS containing 0.05% Tween 20, and then incubated with primary antibody overnight at 4 °C. HRP-conjugated secondary antibody was used and detected using the Pierce ECL Western Blot Substrate. Antibodies were obtained from the following sources: H3K9Ac (Abcam, ab32129, 1:1000), H3K4Me2 (Abcam, ab32356, 1:5000), H3K4Me1 (Abcam, ab8895, 1:5000), H3K4Me3 (Abcam, ab8580, 1:5000), H3K14Ac (Millipore, 07–353, 1:5000), H3K18Ac (Millipore, 07–354, 1:5000), LSD1 (Abcam, ab17721, 1:500), HDAC1 (Abcam, ab19845, 1:1000), total H3 (Cell Signaling Technology, 4499 S, 1:1000 or Abcam, ab1791, 1:10,000), β-actin (Santa Cruz Biotechnology, sc47778, 1:2000), CoREST1 (BD Transduction Laboratories, 612146, 1:500), SIN3A (Abcam, ab129087, 1:1000), HRP-conjugated secondary antibodies (Santa Cruz Biotechnology, 1:5000 for β-actin blots, 1:2000 for all others). Blots shown are representative of at least two independent experiments. Uncropped versions of Western blots are provided in the Supporting Information (Supplementary Figs. 32and 33).

### Solid phase sandwich ELISA

PathScan Acetyl-Histone H3 (Lys9) and Di-Methyl-Histone H3 (Lys4) Sandwich ELISA Kits (Cell signaling 7121 C and 7124 C) were used to detect levels of acetyl-H3 Lys9 and Di-Methyl-H3 Lys4 in cells treated with compounds. Briefly, after 24 h treatment, cells were lysed with 1× Cell Lysis Buffer plus 1 mM PMSF. 5 µg of cell lysate prepared in 100 µl Assay Diluent was added to each well which was pre-coated with total H3 antibody. After overnight incubation at 4 °C, the wells were washed four times with 1× Washing Buffer. 100 µl reconstituted biotinylated acetyl-Histone H3 (Lys9) or di-methyl Histone H3 (Lys4) antibodies were added to each well and incubated for 1 h at 37 °C. After four washes, 100 µl reconstituted HRP-linked streptavidin was added and incubated for 30 min at 37 °C. Following four washes, 100 µl TMB Substrate was added. After a 10 min incubation at 37 °C, 100 µl Stop Solution was added and absorbance at 450 nm was read within 30 min on a SpectraMax microplate reader using the SoftMax Pro software (Molecular Devices, Sunnyvale, CA). Data are representative of two independent experiments with two technical replicates per experiment.

### PicoGreen^®^ cell proliferation assay

Cells were seeded in a 96-well plate and treated with inhibitor at the indicated concentrations. Media was removed and replaced with fresh media containing compound every 24 h. After 72 h incubation, 20 µl lysis buffer (10 mM Tris–HCl, 1 mM EDTA, 0.2% (v/v) Triton X-100) was added to each well and the plate was shaken vigorously on an orbital shaker for 10 min. 70 µl TE buffer was subsequently added to each well and the plate was agitated for 5 min. Then, 95 µl of sample solution from each well was transferred to a new 96-well, non-clear bottom plate (NUNC 236105 96 F) and to each well was added 95 µl 1× PicoGreen^®^ solution (Life Technologies, P11496). The plate was then agitated for 5 min in the dark after which fluorescence was measured using excitation and emission wavelengths of 480 nm and 520 nm, respectively, using a SpectraMax microplate reader. Data represent at least two independent experiments with three technical replicates per experiment. Where *p*-values are reported, the unpaired *t* test was used to determine significance.

### [^3^H]Thymidine incorporation assays

Cells were seeded in 96-well plates (Corning, Corning, NY) for 24 h prior to treatment. HCT116 cells were treated at ~70% confluency and then cultured for an additional 48 h. IC1 cells (5000 cells/well), MET1 cells (2500 cells/well), and primary keratinocytes (3750 cells/well) were treated as indicated and cultured for an additional 72 h. Six hours prior to harvesting cells, 10 µl of a 0.1 mCi ml^−1^ [^3^H]thymidine (American Radiolabeled Chemicals, St. Louis, MO) solution in media was added to each well. Cells were harvested using a Filtermate Harvester (PerkinElmer, Waltham, MA) and radioactivity was measured using a MicroBeta liquid scintillation counter (PerkinElmer, Waltham, MA). Data represent three independent experiments with four technical replicates per experiment. Where *p*-values are reported, the unpaired *t* test was used to determine significance.

### CoREST1 and SIN3A knockdown

shRNA clones targeting CoREST (TRCN0000128570 and TRCN0000129660) or SIN3A (TRCN0000021774) were obtained from the High Throughput Biology Center at Johns Hopkins University.

Lentiviral particles were produced in HEK293T cells using Lipofectamine^®^ 2000 (Invitrogen) according to the manufacturer’s instructions and stored at −80 °C after 0.22 μm filtration. For lentiviral infection, WM983B cells were incubated with CoREST shRNA, SIN3A shRNA, or scramble containing lentiviral particles overnight. Cells were selected with puromycin 48 h after transduction to create stable cell lines. CoREST knockdown was determined by quantitative RT–PCR and confirmed by Western blot. shRNA sequences are provided as Supplementary Table 7.

### Whole genome microarray

WM983B melanoma cells were treated for 24 h with 2.5 µM corin, MS-275, or DMSO as a control. RNA was isolated from cells following the manufacturer’s instructions and cleaned using RNeasy Plus Mini kit (Qiagen Inc.). The isolated RNA was hybridized to the Affymetrix Human Gene 2.0 ST Array. Gene expression levels were analyzed using Affymetrix Human Gene 2.0 ST arrays at the Johns Hopkins Microarray Core Laboratory, including quality control for extracted total RNA samples. Experiments were performed according to the manufacturer’s recommended protocols. In brief, the total extracted RNA was converted into cDNA, amplified, purified, and biotin labeled. Labeled cDNA was hybridized to HuGene2.0_ST array and was scanned using Affymetrix GeneChip scanner 3000 with G7 upgrade (Affymetrix GeneChip Expression Analysis Technical Manual, Affymetrix, Santa Clara, CA). Data are representative of three independent experiments.

### Microarray data analysis

We extracted raw gene expression data, Affymetrix exon CEL files, and RMA (Robust Multi-array Average)^44, 45^ and normalized them using Partek Genomics Suite v6.6 analytic platform (Partek Inc., St. Louis, MO). We converted the replicate cell samples’ gene expression values to log2 notation and then compared the corresponding corin and MS-275 samples using a one-way ANOVA model. We visualized the ANOVA results of this comparison as volcano plots by using the Spotfire DecisionSite platform (TIBCO Software Inc., Palo Alto, CA). We then performed further functional and pathway analyses to illuminate underlying biologic mechanisms. Since the log2 fold changes (log ratios) of the arrays’ 38,598 gene-annotated transcripts presented a normal distribution, their SD (standard deviation) from the mean of no change was used to set the threshold for highly up- and down-regulated genes that were used in pathway analysis. The set of all ANOVA results was exported from Partek to the QIAGEN Ingenuity Pathway Analysis platform (Qiagen Inc., Valencia, CA), where those genes showing greater than 2 SD change up or down were compared to the universe of all the array’s genes. This 2 SD value corresponds to a linear expression fold change of ~1.952 and comprised 1843 unique genes. Ingenuity software used the Fisher exact test to identify biological functions that were statistically enriched for 2 SD differentially expressed genes above what would be expected at random.

### Quantitative real time-PCR

RNA was isolated from primary human melanocytes and melanoma cells following the manufacturer’s instructions and cleaned using the RNeasy Mini kit (Qiagen Inc.). In total 1 µg of RNA was reverse transcribed using SuperScript^®^ III First-Strand Synthesis System kit (Invitrogen). Real-time quantitative PCR was performed for 40 cycles of 15 s at 95 °C and 30 s at 60 °C, using the Step One Plus Real-time PCR system (Applied Biosystems). Data represent two independent experiments with three technical replicates per experiment. The data was calculated using the delta (delta Ct) method. Where *p*-values are reported, the unpaired *t* test was used to determine significance. Transcripts were amplified using the primers listed in Supplementary Table 8.

### Chromatin immunoprecipitation assay (ChIP-PCR)

WM983B cells (10 × 10^6^) were crosslinked in 1% formaldehyde for 7 min and lysed in cell lysis buffer (5 mM HEPES, 85 mM KCl, 0.5% NP-40, 1× protease inhibitor cocktail (Sigma), 1× phosphatase inhibitor cocktail (Sigma)) on ice for 20 min^46^. The nuclei were lysed in buffer containing 50 mM Tris–HCl, pH 8, 10 mM EDTA, 1% SDS and then chromatin was fragmented using the Bioruptor (Diagenode) sonicator to obtain an average size of 250–300 base pairs. Chromatin concentration was measured by its absorbance at 260 nm in the Nanodrop (1 unit ml^−1^ of chromatin corresponds to OD_260_ = 1) and diluted 1:10 with dilution buffer (165 mM NaCl, 0.01% SDS, 1.1% Triton X-100, 1.2 mM EDTA, 16.7 mM Tris-HCl, pH 8.0). 1 unit of chromatin was immunoprecipitated by protein A magnetic beads (Dynabeads, Invitrogen) after incubation with antibodies specific for HDAC1 (Abcam, ab19845, 1:1000) and LSD1 (Abcam, ab17721, 1:500). Normal goat IgG and non-antibody treated samples were used as negative controls. Following overnight immunoprecipitation, beads were washed twice consecutively with each of the following buffers: Lio-B (50 mM HEPES, pH 8.0, 140 mM NaCl, 1% Triton X-100, 0.1% sodium deoxycholate, 1 mM EDTA), Hio-B (50 mM HEPES, pH 8.0, 500 mM NaCl, 1% Triton X-100, 0.1% sodium deoxycholate, 1 mM EDTA), LiCl (10 mM Tris–HCl, 250 mM LiCl, 0.5% NP−40, 0.5% sodium deoxycholate, 1 mM EDTA) and TE (10 mM Tris–HCl, pH 8.0, 1 mM EDTA). Immunoprecipitated chromatin and input DNA were reverse crosslinked in elution buffer (50 mM Tris–HCl, 10 mM EDTA, 1% SDS) in the presence of proteinase K (50 μg ml^−1^) by shaking (1300 RPM) at 68 °C for 5 h. DNA was purified using phenol-chloroform and precipitated in ethanol at −20 °C. DNA pellets were dissolved in 200 μL of ddH_2_O. The relevant primers are listed in Supplementary Table 9.

### Plasma stability studies

Drug-free (blank) human plasma originated from Biological Specialty Corp. (Colmar, PA, USA). Plasma studies were conducted in human plasma and 500 ng ml^−1^ of corin in a final volume of 500 µl. Incubations were performed in duplicate in glass tubes maintained at 37 °C in a shaker bath. Stability studies were terminated immediately or after 0.5 or 1 h by taking 100 µl of the plasma and adding 1 ml of acetonitrile, followed by vortex-mixing then centrifugation for 10 min at 1430x*g*. A 100 µl aliquot of the supernatant was vortex-mixed with 500 µl water prior to injection onto the UItra Performance LC (UPLC) instrument for analysis using a temperature-controlled autosampling device operating at 5 °C.

### Microsomal stability studies

Human and mouse liver microsomes and the NADPH regenerating systems were used to characterize the stability and metabolism of corin that were purchased from Corning Life Sciences (Tewsbury, MA) and BD Gentest Product and Services (Woburn, MA). Metabolism studies were conducted in a 0.5 M sodium-potassium phosphate buffer (pH 7.4) containing 20 mg ml^−1^ of either human or mouse liver microsomes, an NADPH-generating system, and 10 mM of corin in a final volume of 500 µl. Negative controls were performed without an NADPH-generating system to control for native enzyme activities. Incubations were performed in duplicate in glass tubes maintained at 37 °C in a shaker bath. The microsomal metabolism was terminated immediately or after 0.5 or 1 h by taking 20 µl of the microsome mixture and adding 1 ml of acetonitrile, followed by vortex-mixing then centrifugation for 10 min at 1430×*g*. A 10 µl aliquot of the supernatant was injected onto the UPLC instrument for qualitative analysis using a temperature-controlled autosampling device operating at 5 °C.

### LCMS analysis

Chromatographic analysis was performed using a Waters Acquity^TM^ UItra Performance UPLC. Separation of the analyte from potentially interfering material and metabolites was achieved at ambient temperature using Waters Cortecs column (50 × 2.1 mm, L x I.D.) with a 2.7 µm particle size. (Milford, MA). The mobile phase used for the chromatographic separation was composed of 0.1% (v/v) formic acid in water (mobile phase A) and 0.1% (v/v) formic acid in methanol (mobile phase B) with a flow rate of 0.3 ml min^−1^. The initial mobile phase composition was 90% mobile phase A and 10% mobile phase B. From 0.5 to 6.0 min, mobile phase B was increased linearly from 10 to 100% and maintained until 7.0 min. From 7.0 to 7.1 min, the gradient decreased to 10% mobile phase B and the conditions were maintained until 8 min to re-equilibrate the column for the next injection. The column effluent was monitored using an AB Sciex 5500 triple-quadruple mass-spectrometric detector. The instrument was equipped with an electrospray interface, operated in positive mode and controlled by the Analyst v1.6 software. For the stability study, the mass spectrometer was programmed to monitor an MRM transition 429.1 → 394.0 for corin. Results were assessed qualitatively comparing the average area ratio of corin at 0 h to the area ratio at 0.5 h and 1 h for both mouse and human liver microsomes or plasma.

### SK-MEL-5 melanoma xenograft

All animal studies were conducted in the Animal Facility at Shanghai Bioduro Co. in accordance with Bioduro Institutional Animal Care and Use Committee Guidance.

For the toxicity study, 6 Balb/c nude mice were randomly divided into two groups: vehicle and corin (*n* = 3 animals per group). Animals were given vehicle (5% DMSO/H_2_O) or corin (30 mg kg^−1^ at 10 ml kg^−1^) by intraperitoneal injection (IP) once a day. Animal body weights were measured daily and clinical observations were recorded daily for 10 days. Clinical observations include appearance observation (hair coat, discharges, injury, lesion and eye) and behavior/condition observation (gait, activity, nervous system, respiration and feces).

For the SK-MEL-5 xenograft studies, six to eight-week old female Balb/C nude mice were purchased from Lingchang (Shanghai, China) and allowed to acclimate for 1 week prior to beginning the experiment. For each animal, SK-MEL-5 (6 × 10^6^) cells in 150 µl growth media mixed with 50% matri-gel (BD, USA) were injected into the subcutaneous tissue of the flank^29, 47^. When xenograft size reached an approximate volume of 150 mm^3^, the 20 mice were randomized into 10 mice per group with the average tumor volume distributed equally between groups (vehicle: avg. initial mouse body weight was 18.0 ± 0.3 g, avg. tumor volume was 150 ± 9 mm^3^; corin: avg. initial mouse body weight was 18.3 ± 0.2 g, avg. initial tumor volume was 151 ± 7 mm^3^). Vehicle control (5% DMSO/H_2_O) and corin (30 mg kg^−1^) were administered at 10 ml kg^−1^ by IP once a day. Of note, a third arm also containing 10 animals (avg. initial body weight was 18.4 ± 0.3 g, avg. initial tumor volume was 151 ± 7 mm^3^) was included to evaluate MS-275 (30 mg kg^−1^) head to head with corin. However, six of the ten animals died within one week of initiating the study leading to early termination of this arm for ethical reasons. The mice were maintained in a pathogen-free environment with free access to food and water. Body weight and tumor volume were measured twice a week. Tumor size was measured with linear calipers and calculated using the formula: ((length in millimeters × (width in millimeters)^2^)/2). The mice were sacrificed after 4 weeks and tumor weights were measured. Three animals from each treatment group were chosen at random for follow up blood work. Where *p*-values are reported, the unpaired *t* test was used to determine significance.

### Xenograft tumor sample processing and immunohistochemistry

After sacrificing the mice, tumors were removed and post-fixed in 3.7% formaldehyde. Serial sections 5 μm thick were cut from the formalin fixed, paraffin embedded tissue blocks, floated onto charged glass slides (Super-Frost Plus, Fisher Scientific), and dried overnight at 60 °C. Sections were deparaffinized and hydrated using graded concentrations of ethanol to deionized water prior to immunohistochemistry. Tumor sections were blocked in serum (5% serum in PBS-T (0.5% TritonX-100 in PBS)), and then incubated overnight at 4 °C in rabbit anti-Ki67 (Abcam, ab15580, 1:500). Sections were then incubated with fluorescence-conjugated (FITC) goat anti-rabbit secondary antibody for 1 h at room temperature, washed with PBS, and mounted using VECTASHIELD mounting medium (Vector Laboratories).

### Data availability

Microarray data have been deposited in the NCBI GEO database with accession number GSE87289. The data that support this study are available as Supplementary Data 1. The additional data that support the findings of this study are available from the corresponding authors upon request.

## Electronic supplementary material

Supplementary Information

Description of Additional Supplementary Files

Supplementary Data 1
