# Supplementary material for: Targeting the CoREST complex with dual histone deacetylase and demethylase inhibitors
Source: Nat Commun. 2018 Jan 4;9:53. doi: 10.1038/s41467-017-02242-4 (PMC5754352; doi:10.1038/s41467-017-02242-4)
Supplement: Supplementary file 1 — Supplementary Information [file 41467_2017_2242_MOESM1_ESM.pdf]

Targeting the CoREST Complex with Dual Histone Deacetylase and Demethylase Inhibitors

Supplementary Information

Table of Contents:

I. Supplementary Figures (S2-S44)
II. Supplementary Tables (S45-S55)
III. Supplemtary Methods (S56-S93)
IV. Supplementary References (S94-S95)

a **b** **c**

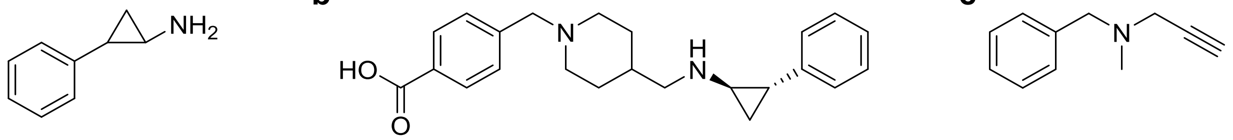

Tranylcypromine **GSK2879552** **Pargyline**

Supplementary Fig. 1 Structures of several amine oxidase inhibitor compounds screened in this study. **a** Tranylcypromine, MAO A/B and LSD1 inhibitor. **b** GSK2879552, selective LSD1 inhibitor, **c** Pargyline, selective MAO A/B inhibitor**.**

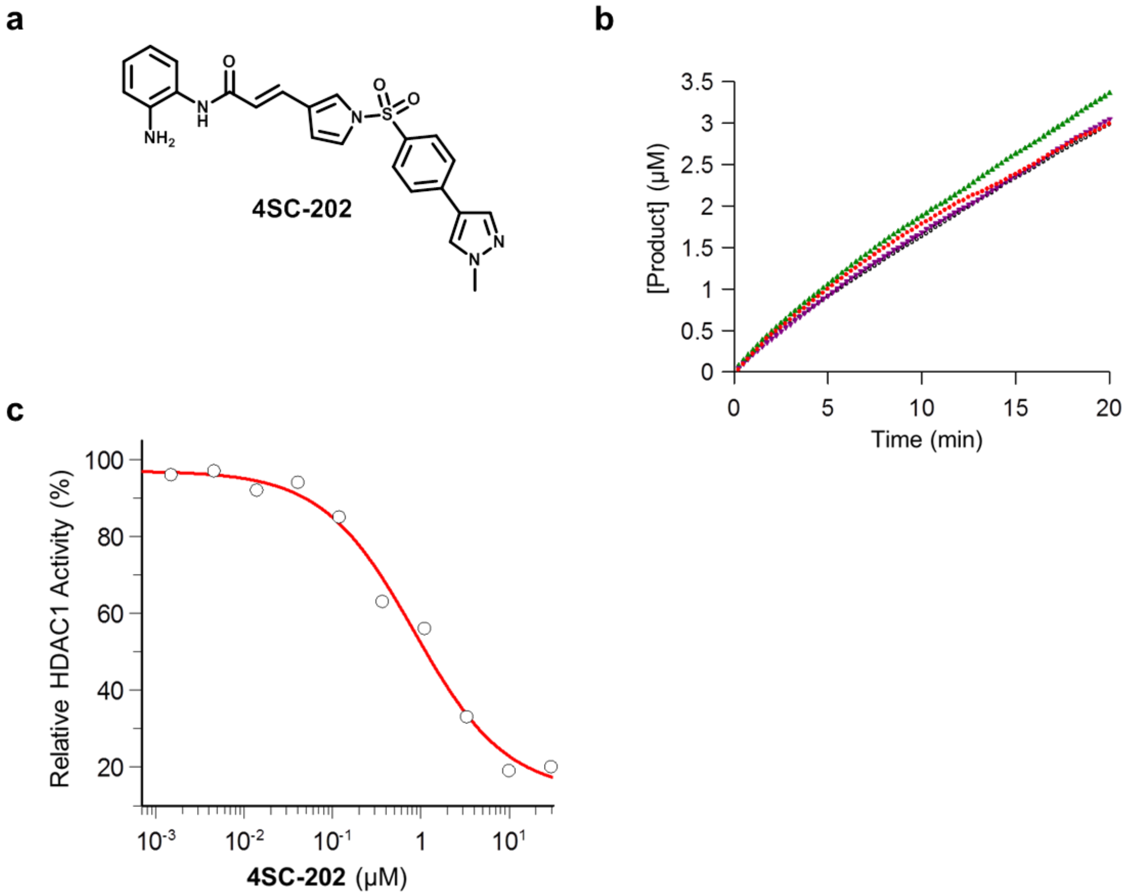

Supplementary Fig. 2 Kinetic data for the inhibition of LSD1 and HDAC1 by 4SC-202. **a** Structure of reported LSD1-HDAC dual inhibitor 4SC-202. **b** Steady-state progress curves generated by treating LSD1 with varying concentrations of inhibitor ([LSD1] = 110 nM, 300 µM dimethyl histone $H3K4_{1\text{-}21}$ peptide was used as substrate). No appreciable LSD1 inhibition was observed at concentrations up to 20 µM (hollow circles: vehicle, green: 5 µM, purple: 10 µM, red: 20 µM). **c** HDAC IC_{50} curve generated for 4SC-202. IC_{50} = 0.841 ± 0.230 µM, 5% DMSO/H_2O was used as the vehicle since the compound was not soluble in the standard 2% DMSO/H_2O ([HDAC1] = 2.86 nM, 20 µM acetylated $P53_{379\text{-}382}$ tetrapeptide RHKK(Ac) was used as substrate). Data are representative of three independent experiments.

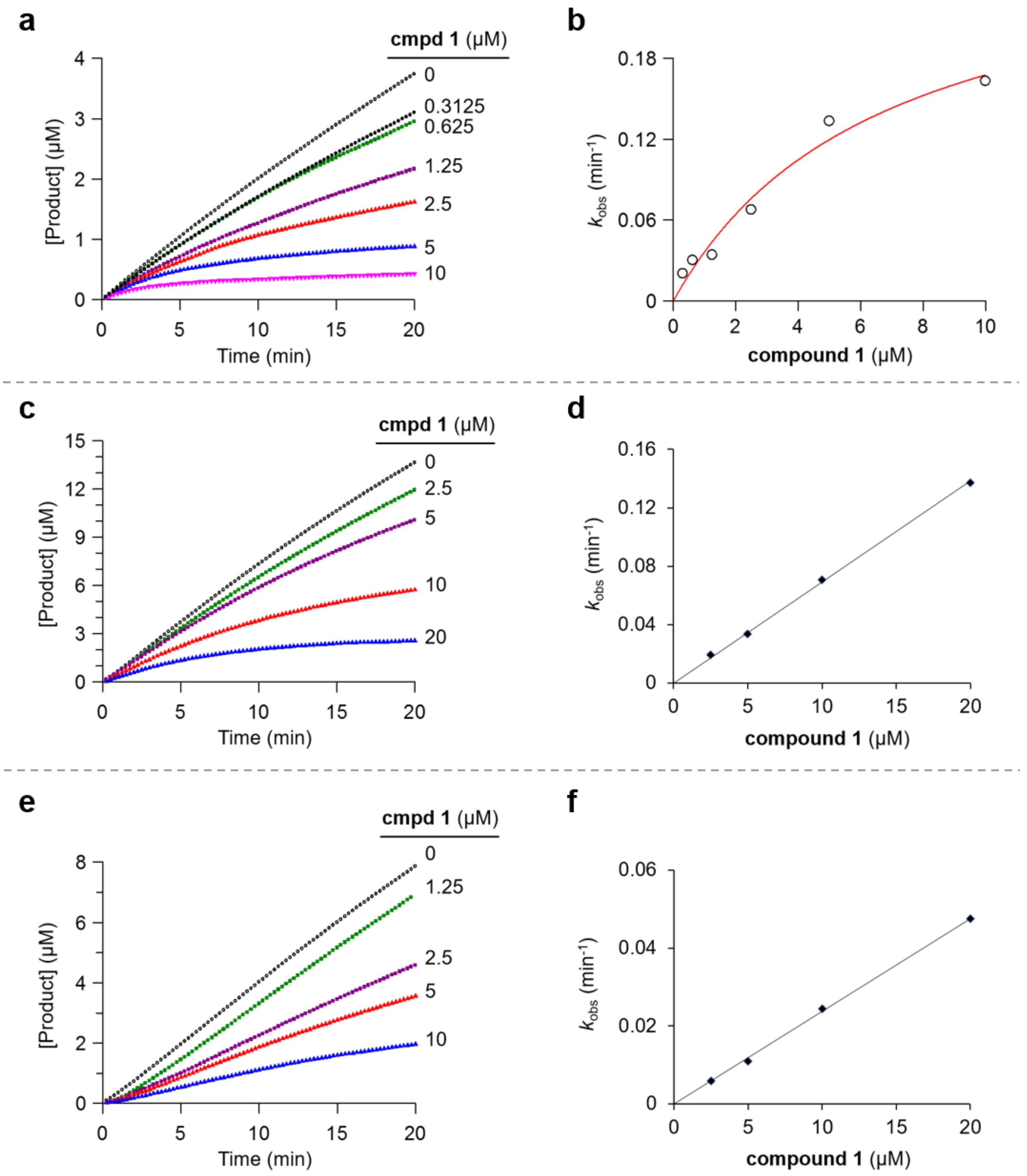

Supplementary Fig. 3 Representative kinetic data for compound **1**. Steady-state progress curves generated by the inactivation of LSD1 (**a**), MAO A (**c**), MAO B (**e**) with varying concentrations of inhibitor. Rate constants (k_{obs}) were determined and plotted against inhibitor concentration. The data was fit to the Kitz-Wilson equation to determine k_{inact} and $K_{i(inact)}$ values for LSD1 (**b**) or fit to a linear regression with the slope representing $k_{inact}/K_{i(inact)}$ for MAO A (**d**), MAO B (**f**). Data are representative of at least two independent experiments.

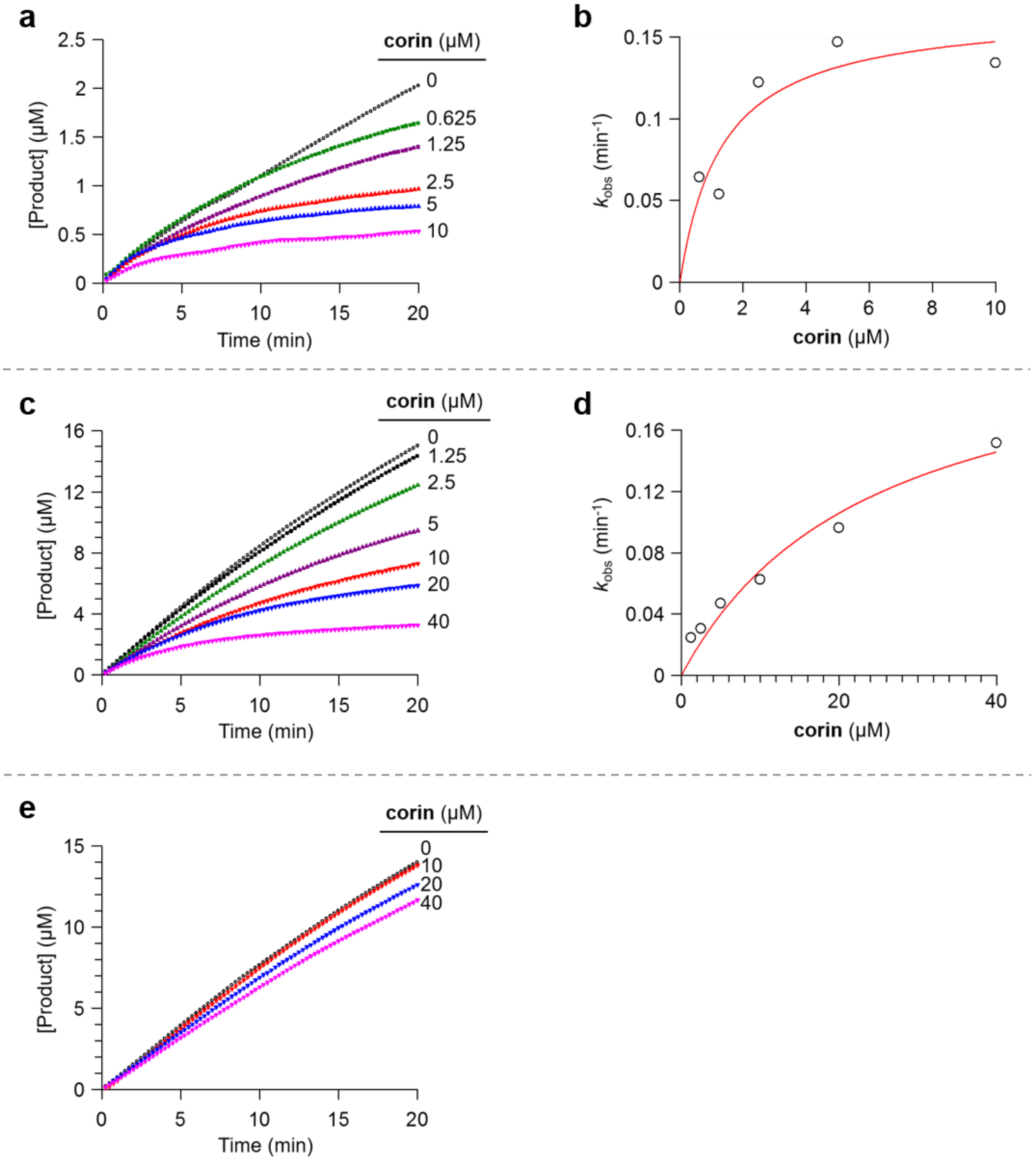

Supplementary Fig. 4 Representative kinetic data for corin. Steady-state progress curves generated by the inactivation of LSD1 (**a**), MAO A (**c**), MAO B (**e**) with varying concentrations of inhibitor. Rate constants (k_{obs}) were determined and plotted against inhibitor concentration. The data was fit to the Kitz-Wilson equation to determine k_{inact} and $K_{i(inact)}$ values for LSD1 (**b**), MAO A (**d**). Data are representative of at least two independent experiments.

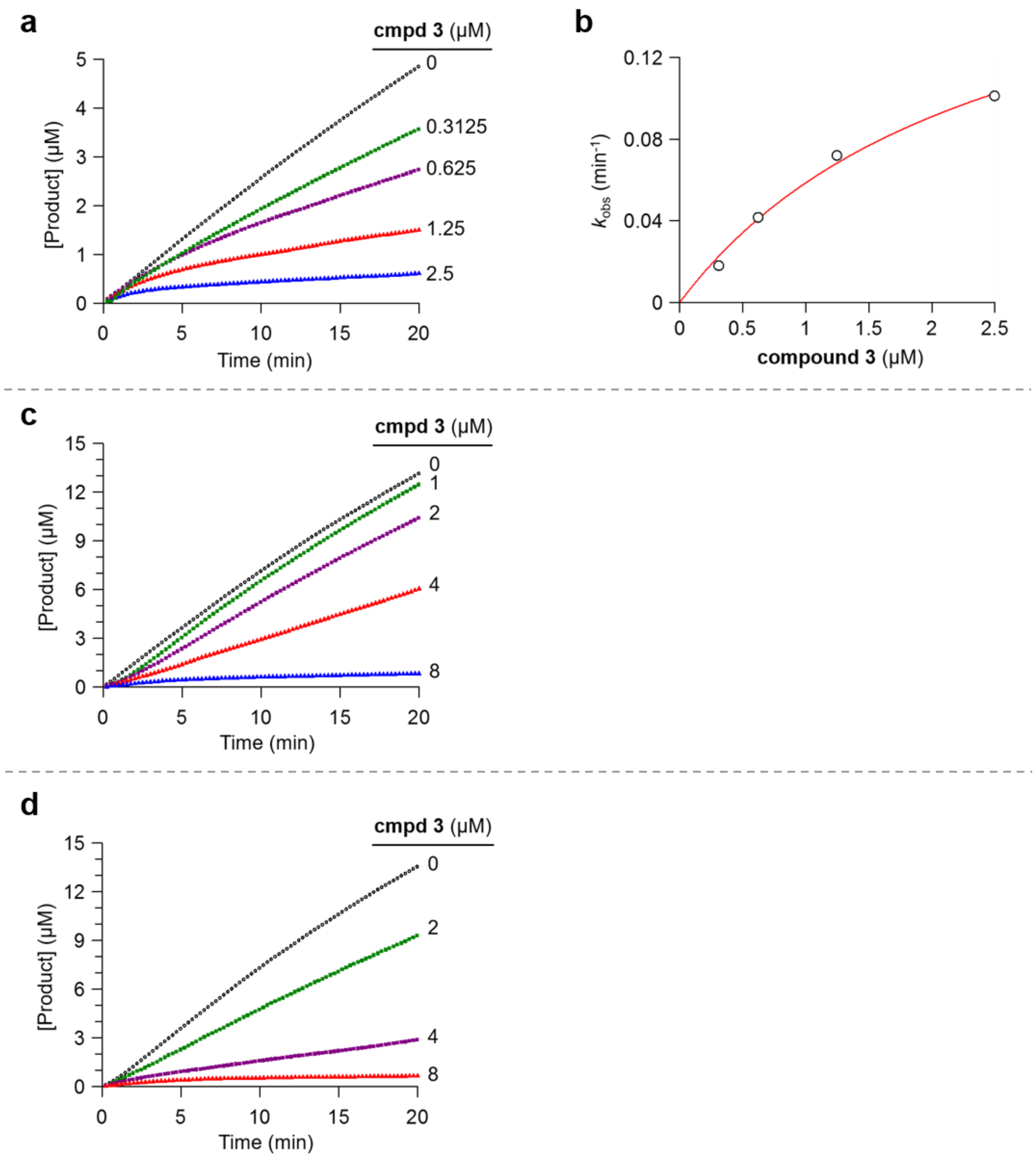

Supplementary Fig. 5 Representative kinetic data for compound **3**. Steady-state progress curves generated by the inactivation of LSD1 (**a**), MAO A (**c**), MAO B (**d**) with varying concentrations of inhibitor. Rate constants (k_{obs}) were determined and plotted against inhibitor concentration. The data was fit to the Kitz-Wilson equation to determine k_{inact} and $K_{i(inact)}$ values for LSD1 (**b**). Inhibitor efficiency ($k_{inact}/K_{i(inact)}$) was estimated from steady-state progress curves for MAO A and MAO B. Data are representative of at least two independent experiments.

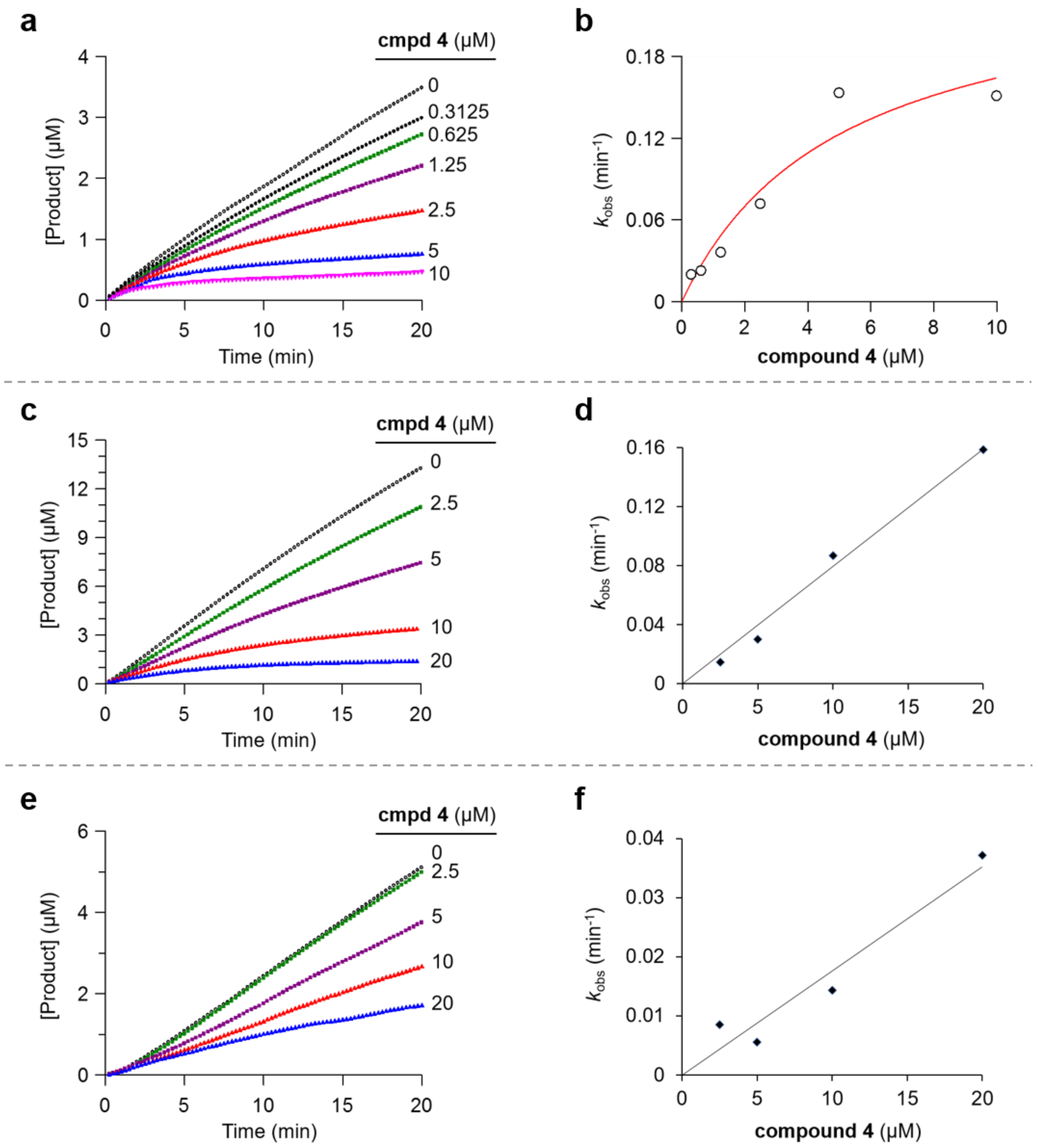

Supplementary Fig. 6 Representative kinetic data for compound **4**. Steady-state progress curves generated by the inactivation of LSD1 (**a**), MAO A (**c**), MAO B (**e**) with varying concentrations of inhibitor. Rate constants (k_{obs}) were determined and plotted against inhibitor concentration. The data was fit to the Kitz-Wilson equation to determine k_{inact} and $K_{i(inact)}$ values for LSD1 (**b**) or fit to a linear regression with the slope representing $k_{inact}/K_{i(inact)}$ for MAO A (**d**), MAO B (**f**). Data are representative of at least two independent experiments.

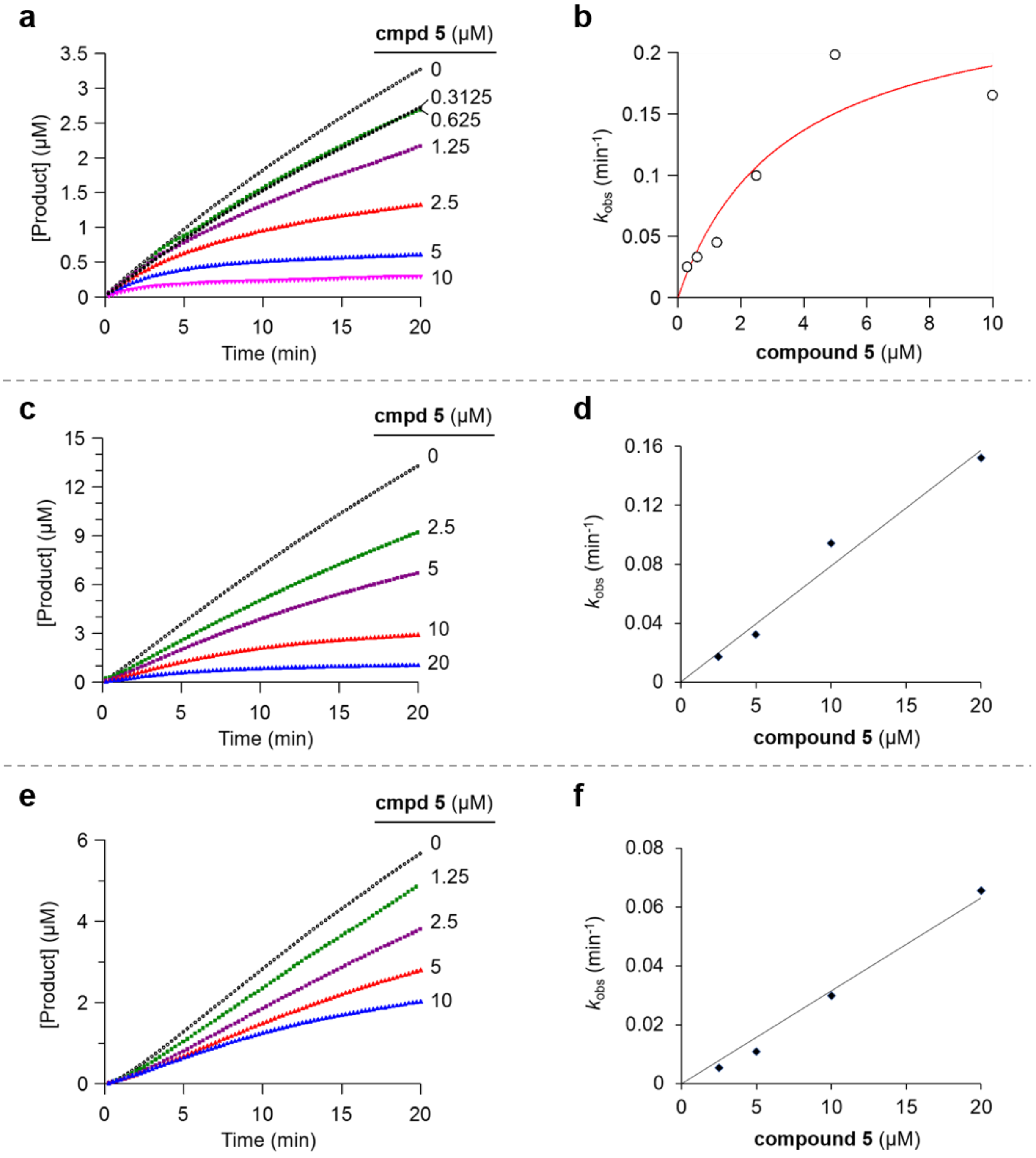

Supplementary Fig. 7 Representative kinetic data for compound **5**. Steady-state progress curves generated by the inactivation of LSD1 (**a**), MAO A (**c**), MAO B (**e**) with varying concentrations of inhibitor. Rate constants (k_{obs}) were determined and plotted against inhibitor concentration. The data was fit to the Kitz-Wilson equation to determine k_{inact} and $K_{i(inact)}$ values for LSD1 (**b**) or fit to a linear regression with the slope representing $k_{inact}/K_{i(inact)}$ for MAO A (**d**), MAO B (**f**). Data are representative of at least two independent experiments.

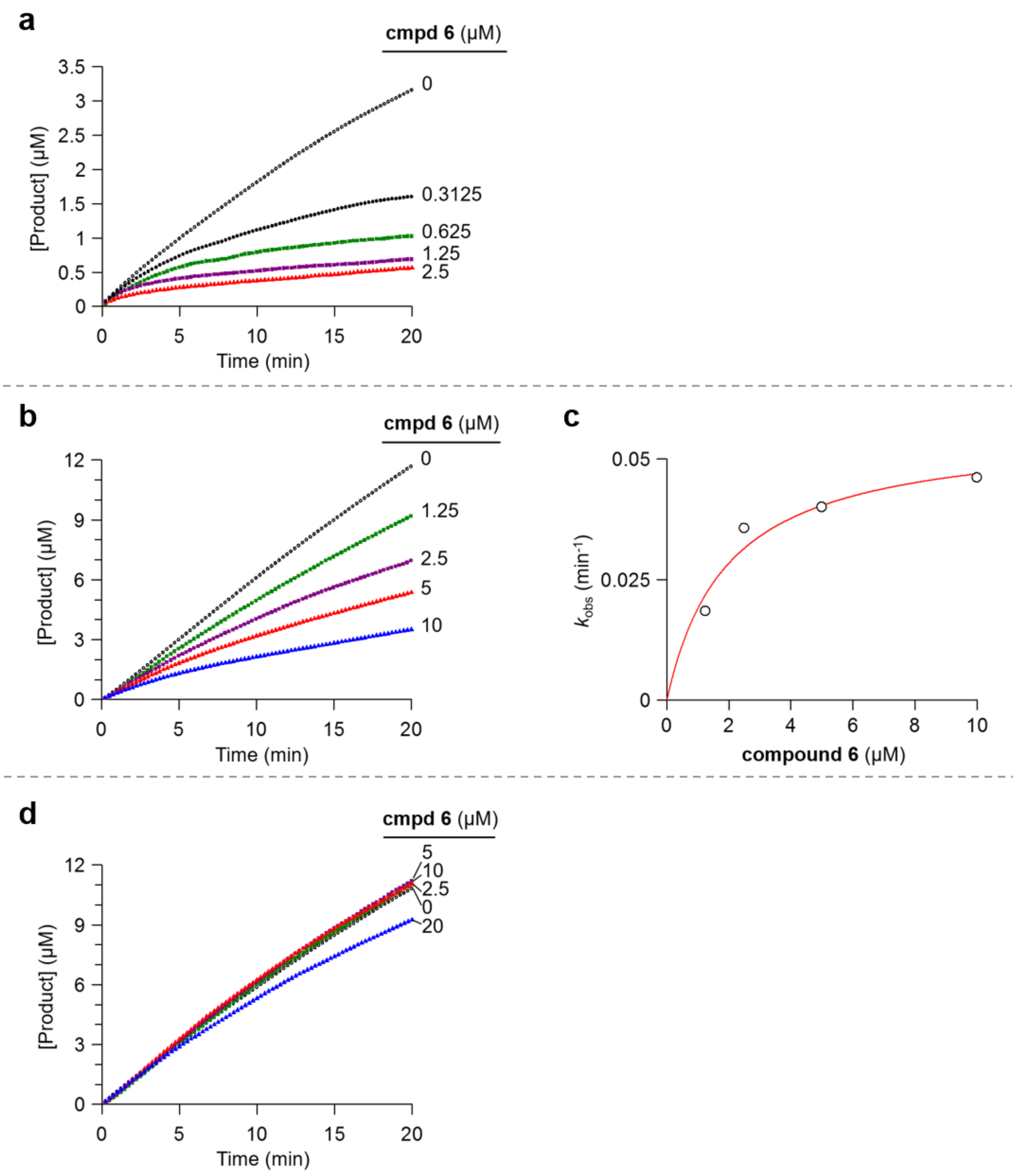

Supplementary Fig. 8 Representative kinetic data for compound **6**. Steady-state progress curves generated by the inactivation of LSD1 (**a**), MAO A (**b**), MAO B (**d**) with varying concentrations of inhibitor. Rate constants (k_{obs}) were determined and plotted against inhibitor concentration. The data was fit to the Kitz-Wilson equation to determine k_{inact} and $K_{i(inact)}$ values for MAO A (**c**). Inhibitor efficiency ($k_{inact}/K_{i(inact)}$) was estimated from steady-state progress curves for LSD1. Data are representative of at least two independent experiments.

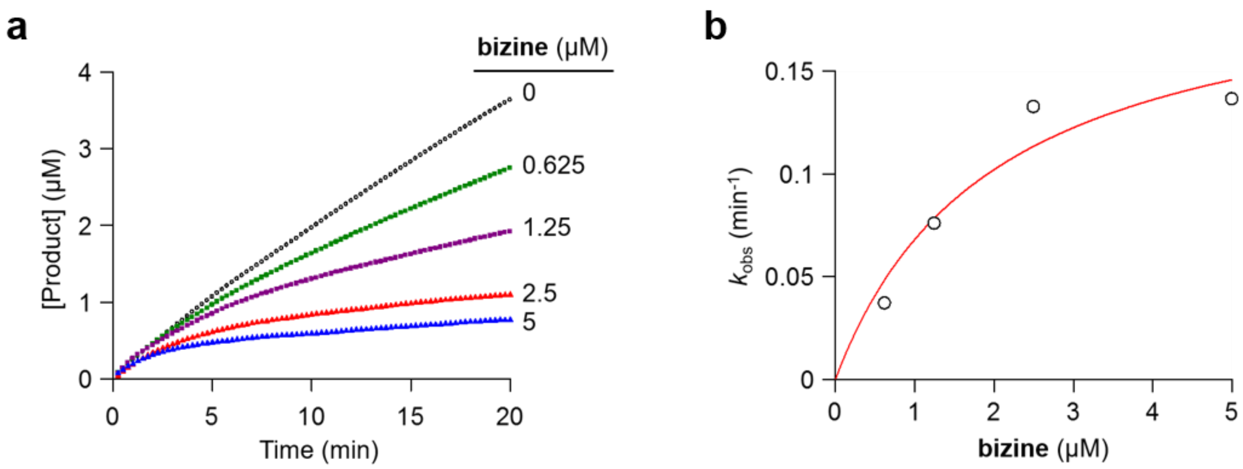

Supplementary Fig. 9 Representative kinetic data for bizine. Steady-state progress curves generated by the inactivation of LSD1 (**a**) with varying concentrations of inhibitor. Rate constants (k_{obs}) were determined and plotted against inhibitor concentration. The data was fit to the Kitz-Wilson equation to determine k_{inact} and $K_{i(inact)}$ values for LSD1 (**b**). Data are representative of at least two independent experiments.

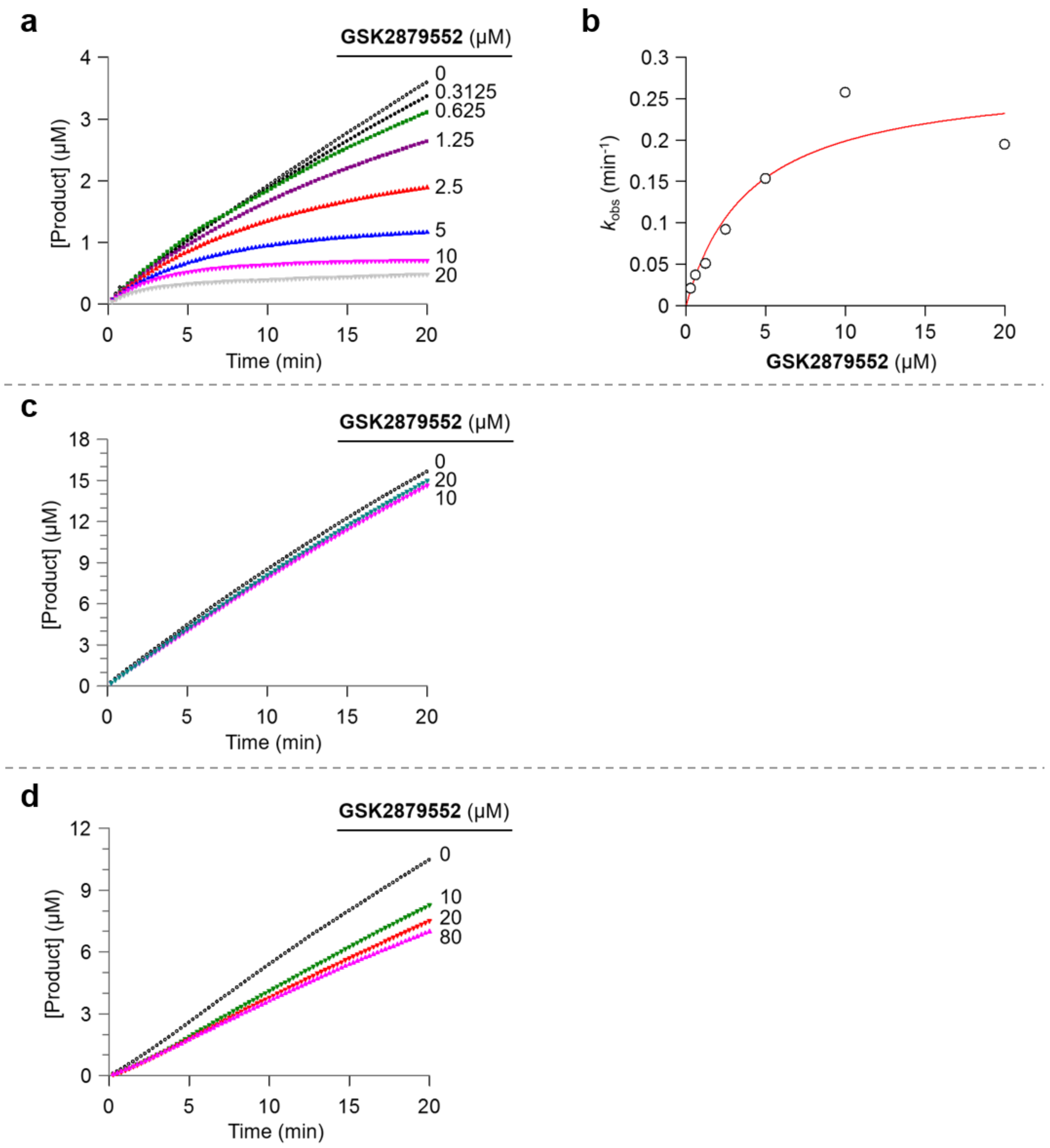

Supplementary Fig. 10 Representative kinetic data for GSK2879552. Steady-state progress curves generated by the inactivation of LSD1 (**a**), MAO A (**c**), MAO B (**d**) with varying concentrations of inhibitor. Rate constants (k_{obs}) were determined and plotted against inhibitor concentration. The data was fit to the Kitz-Wilson equation to determine k_{inact} and $K_{i(inact)}$ values for LSD1 (**b**). Data are representative of at least two independent experiments.

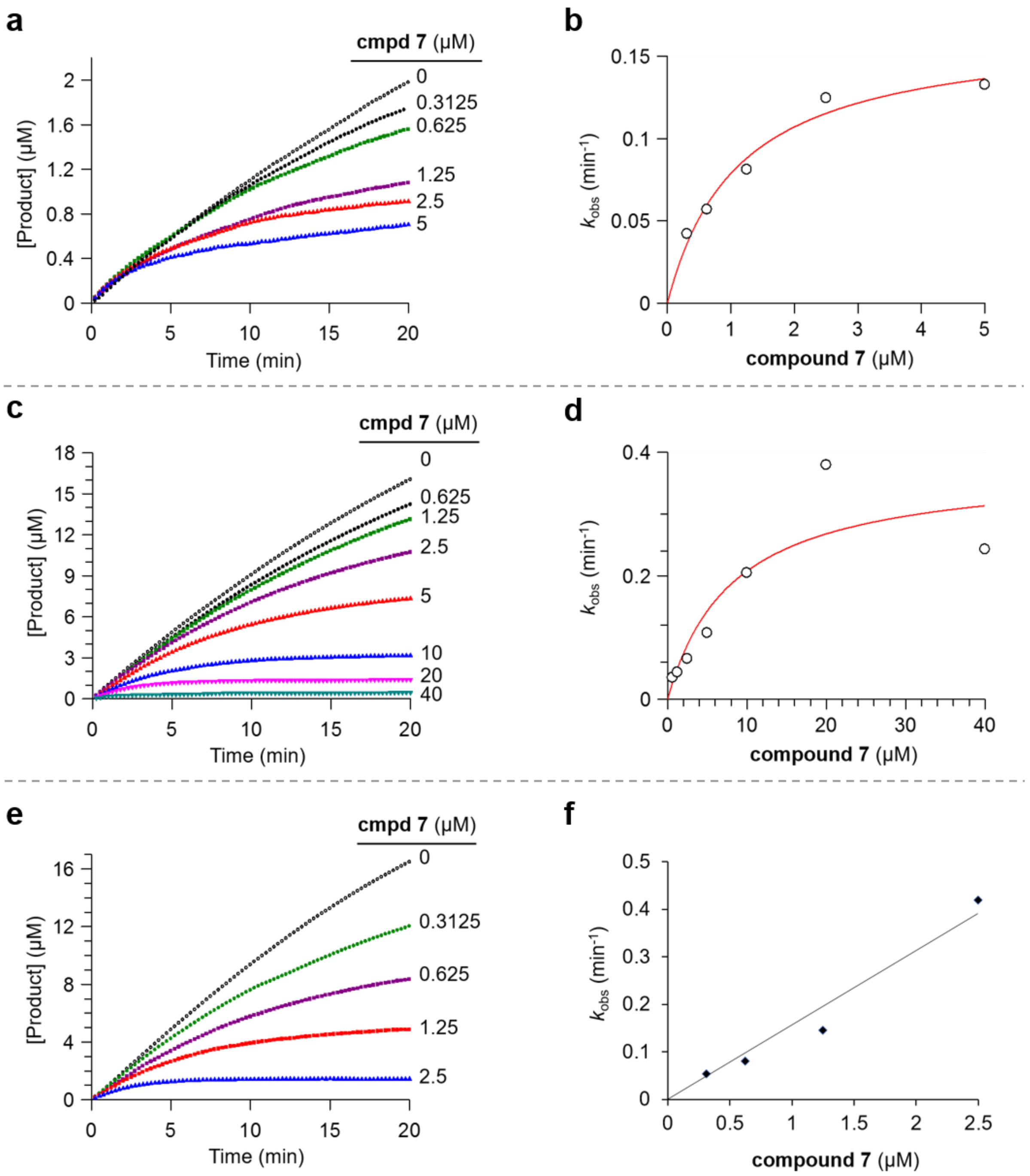

Supplementary Fig. 11 Representative kinetic data for compound **7**. Steady-state progress curves generated by the inactivation of LSD1 (**a**), MAO A (**c**), MAO B (**e**) with varying concentrations of inhibitor. Rate constants (k_{obs}) were determined and plotted against inhibitor concentration. The data was fit to the Kitz-Wilson equation to determine k_{inact} and $K_{i(inact)}$ values for LSD1 (**b**), MAO A (**d**) or fit to a linear regression with the slope representing $k_{inact}/K_{i(inact)}$ for MAO B (**f**). Data are representative of at least two independent experiments.

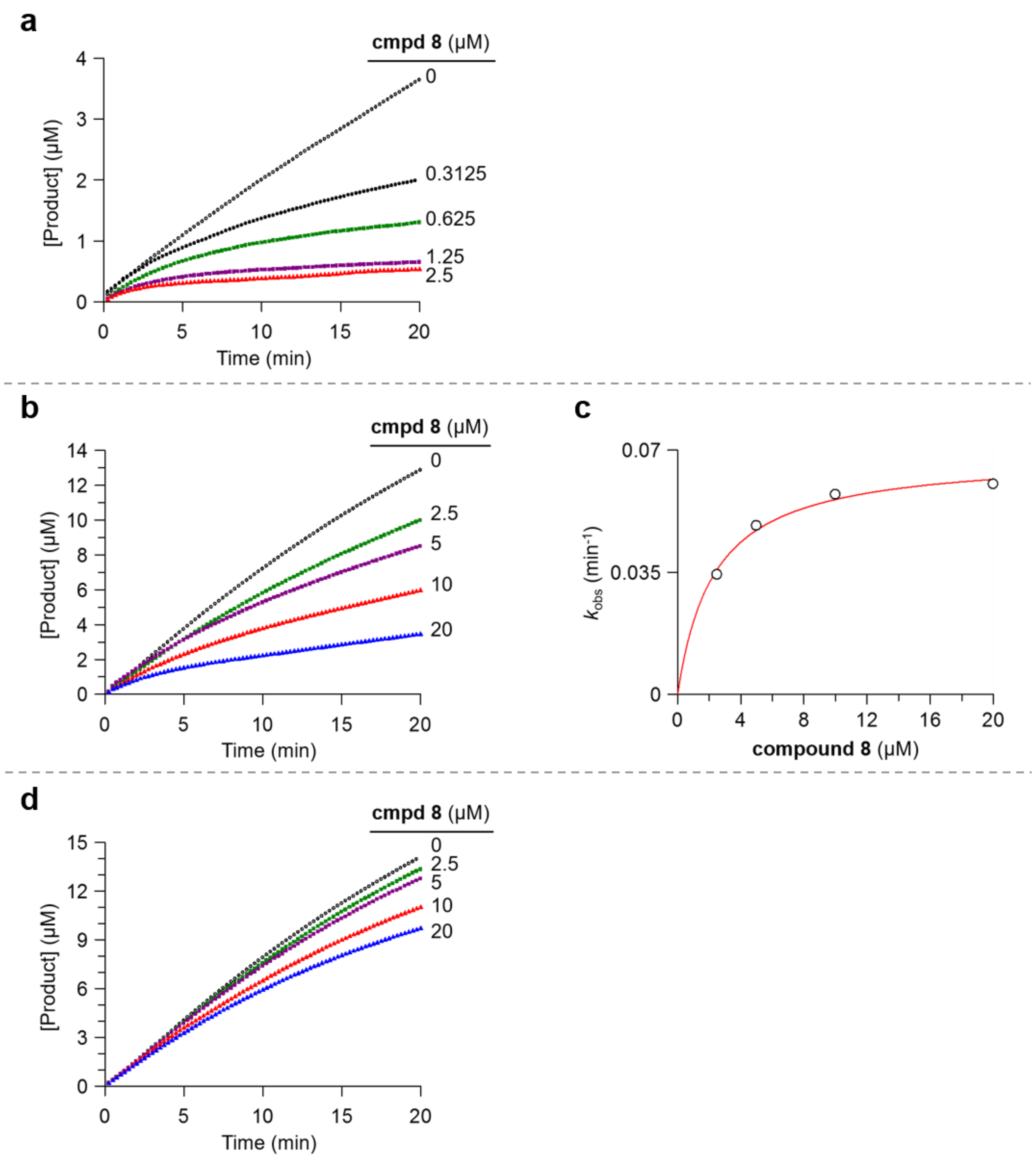

Supplementary Fig. 12 Representative kinetic data for compound **8**. Steady-state progress curves generated by the inactivation of LSD1 (**a**), MAO A (**b**), MAO B (**d**) with varying concentrations of inhibitor. Rate constants (k_{obs}) were determined and plotted against inhibitor concentration. The data was fit to the Kitz-Wilson equation to determine k_{inact} and $K_{i(inact)}$ values for MAO A (**c**). Inhibitor efficiency ($k_{inact}/K_{i(inact)}$) was estimated from steady-state progress curves for LSD1. Data are representative of at least two independent experiments.

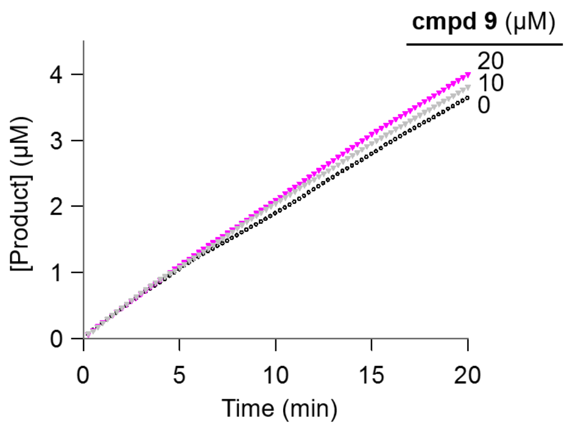

Supplementary Fig. 13 Representative kinetic data for compound **9**. Steady-state progress curves generated by the inactivation of LSD1 by compound **9**. No appreciable inhibition of LSD1 was observed at concentrations up to 20 μM. Data are representative of at least two independent experiments.

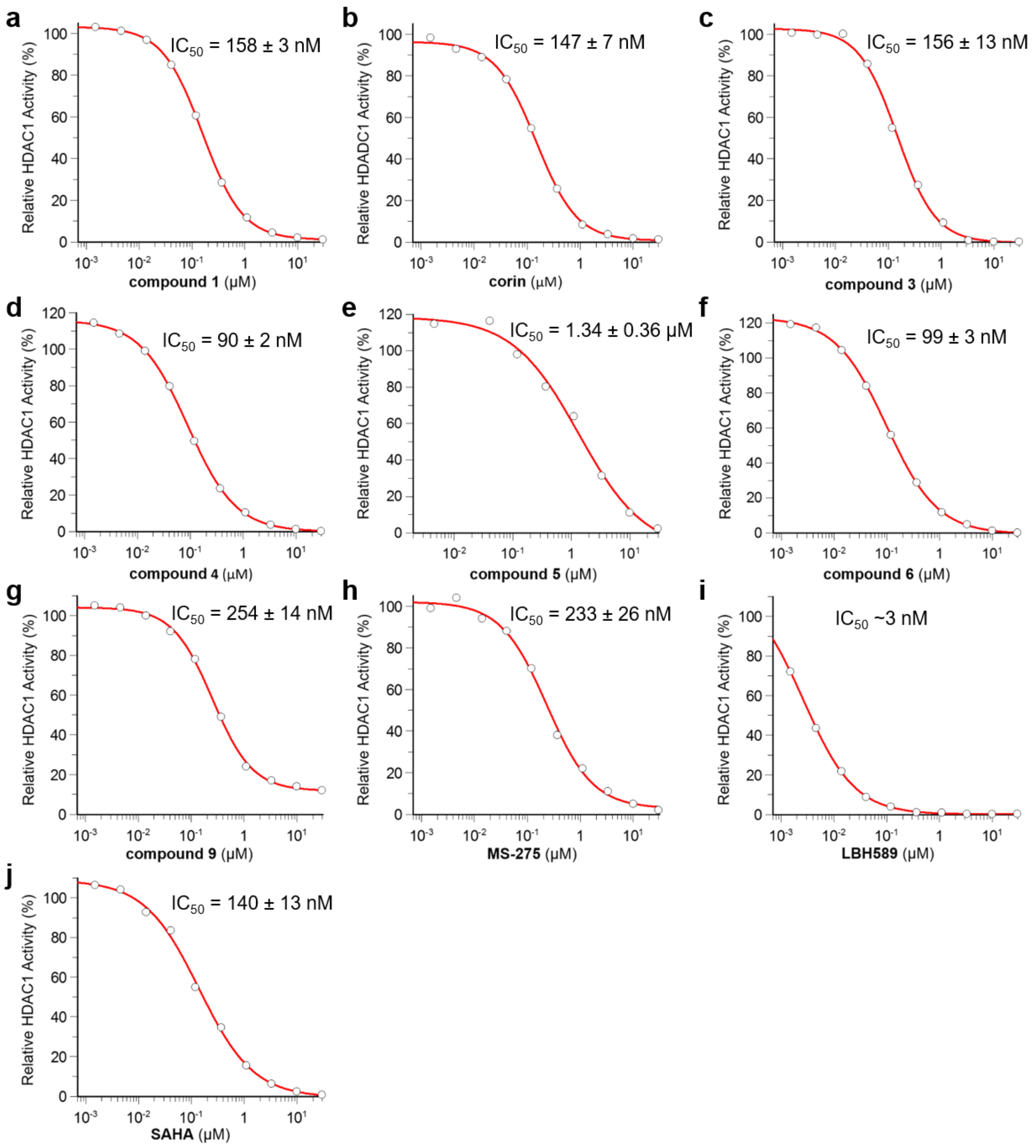

Supplementary Fig. 14 Inhibition of isol. HDAC1 by dual inhibitors and known HDAC inhibitors. Dose response curves generated for compound **1** (**a**), corin (**b**), compound **3** (**c**), compound **4** (**d**), compound **5** (**e**), compound **6** (**f**), compound **9** (**g**), MS-275 (**h**), LBH589 (**i**), SAHA (**j**). Data (± SE) are representative of at least three independent experiments ([HDAC1] = 2.86 nM, 20 μM acetylated $P53_{379\text{-}382}$ tetrapeptide RHKK(Ac) was used as substrate).

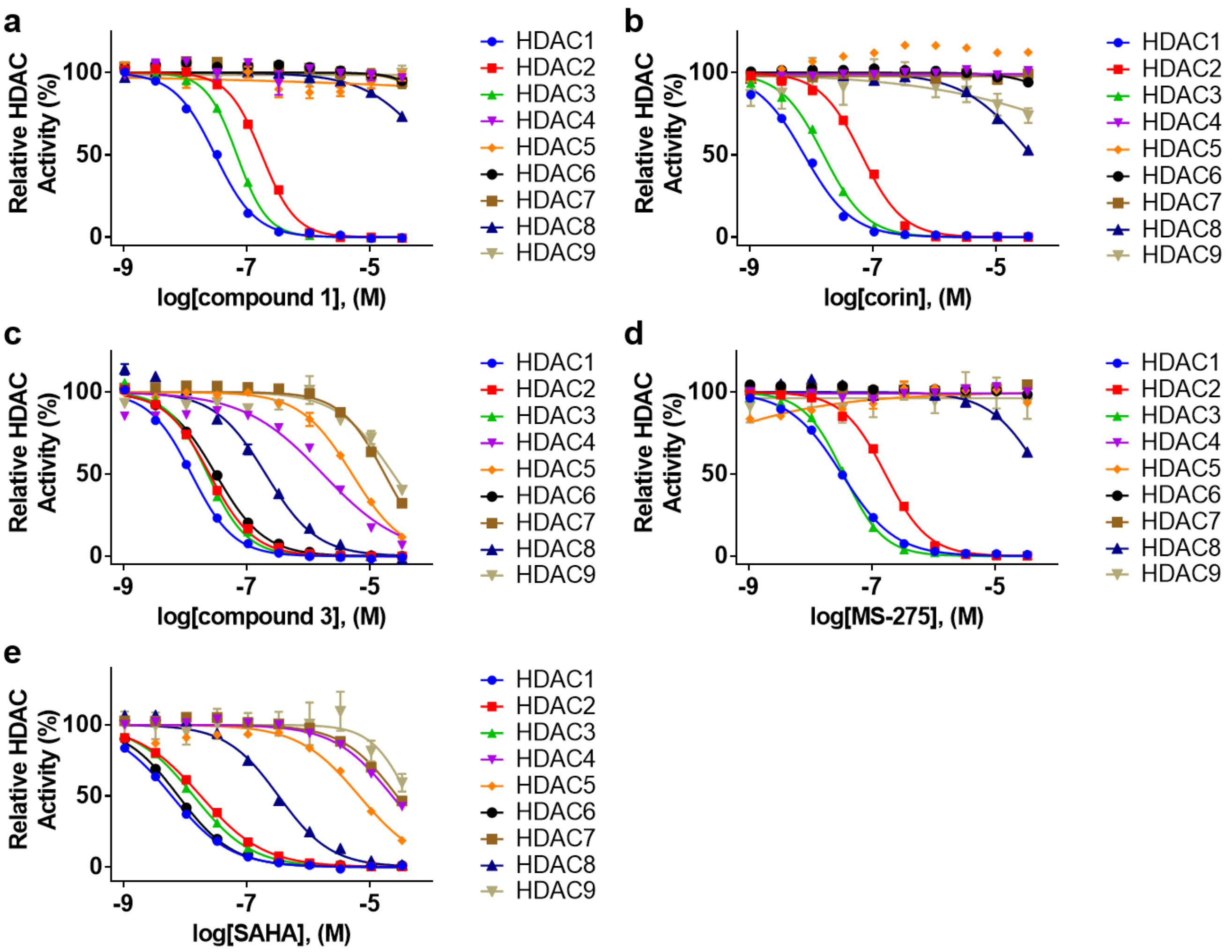

Supplementary Fig. 15 Dose-dependent inhibition of HDAC isoforms 1-9 by compound **1** (**a**), corin (**b**), compound **3** (**c**), MS-275 (**d**), SAHA (**e**). IC_{50} < 200 nM for compound **1**, corin, and MS-275 against HDACs 1-3 but IC_{50} > 30 µM for HDACs 4-9. Data (mean ± SE) were obtained using the biochemical HDAC isoform inhibition assay.

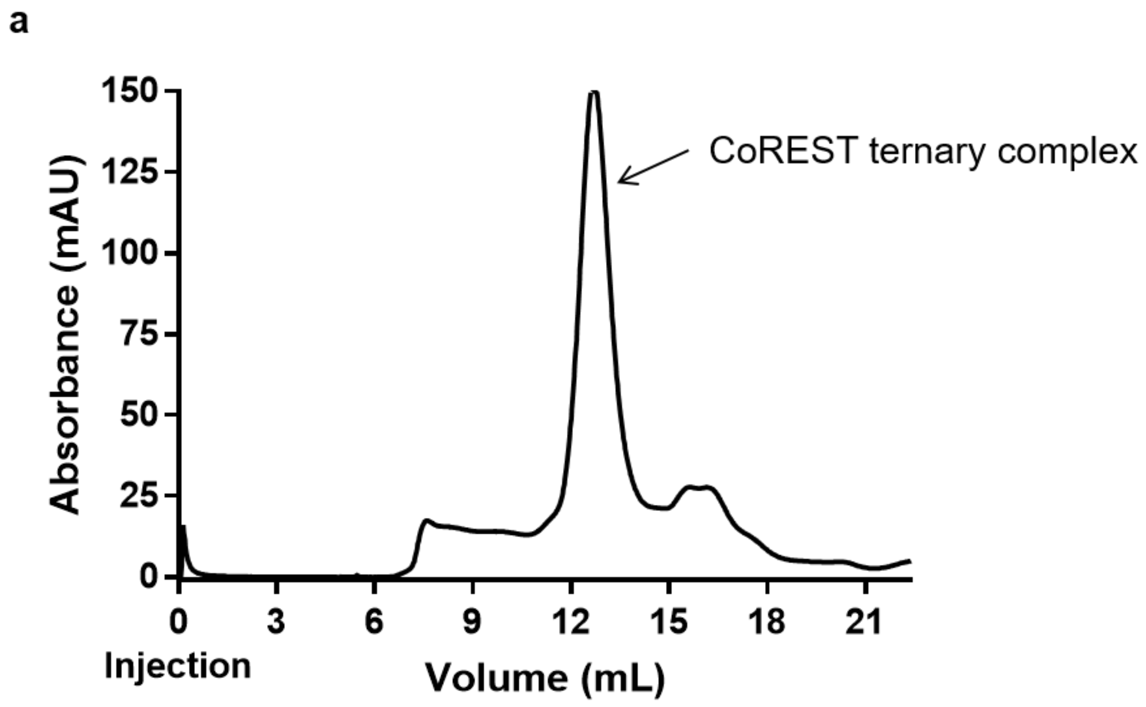

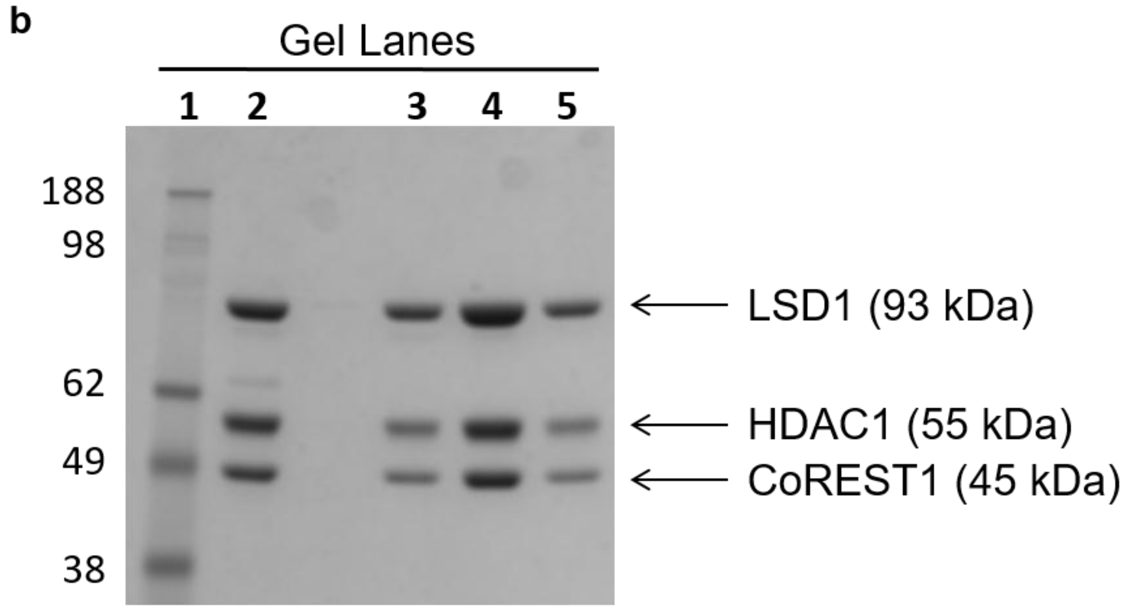

Supplementary Fig. 16 The CoREST ternary complex is stably associated and exhibits 1:1:1 stoichiometry. **a** Purification of the CoREST ternary complex after TEV cleavage from the FLAG resin by size exclusion chromatography. **b** SDS-PAGE gel illustrating the 1:1:1 stoichiometry of the CoREST complex. Lane 1: ladder, lane 2: complex before purification by size exclusion chromatography, lanes 3-5: fractions collected during size exclusion chromatography.

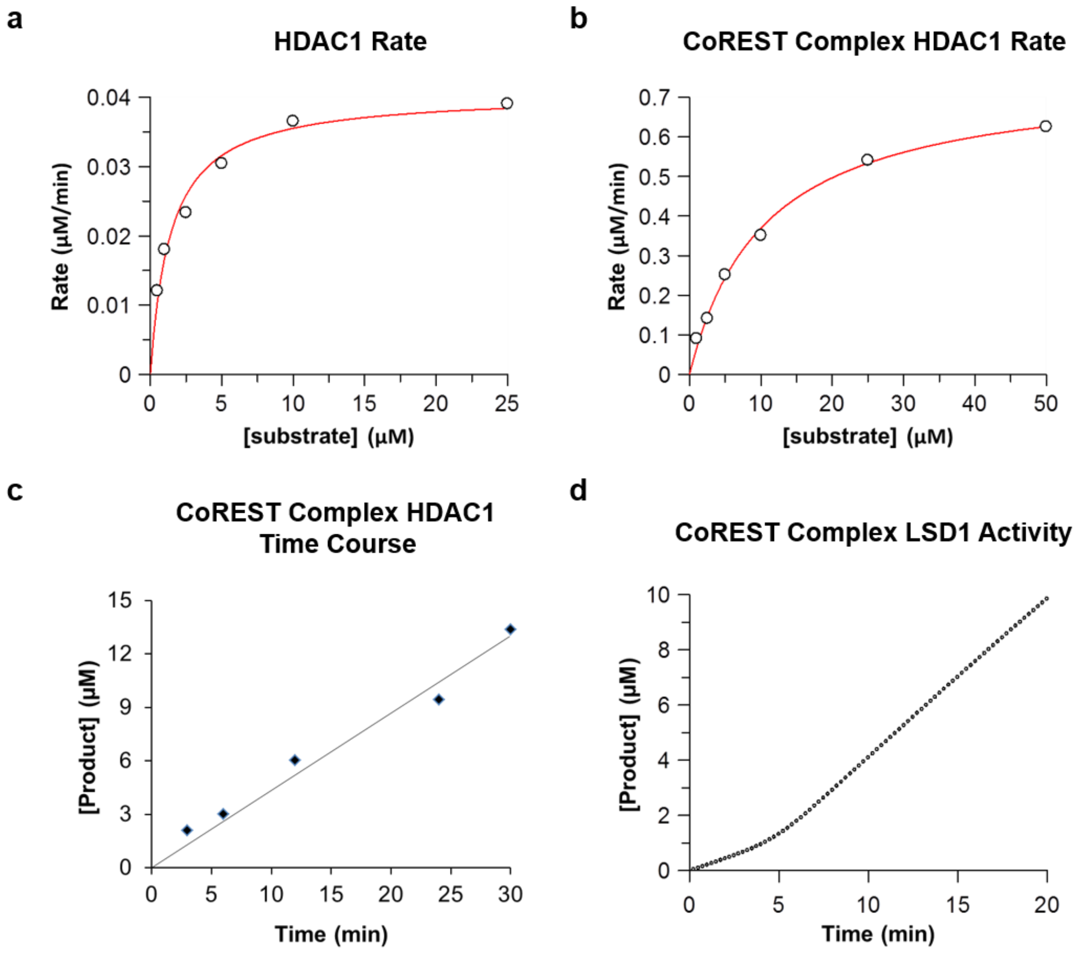

Supplementary Fig. 17 Kinetic analysis of the CoREST ternary complex. **a** Michaelis-Menten plot for characterization of isol. HDAC1 kinetics (k_{cat} = 14.20 ± 0.56 min^{-1}, K_m = 1.43 ± 0.22 µM, [HDAC1] = 2.86 nM). **b** Michaelis-Menten plot for characterization of CoREST complex HDAC1 kinetics (k_{cat} = 378.8 ± 13.5 min^{-1}, K_m = 10.51 ± 1.03 µM, [CoREST complex] = 2 nM). **c** Time course illustrating the linear nature of CoREST complex HDAC1 kinetics ([CoREST complex] = 2 nM, 50 µM acetylated $P53_{379-382}$ tetrapeptide (RHKK(Ac) substrate). **d** Time course illustrating the bi-phasic nature of CoREST complex LSD1 kinetics ([CoREST complex] = 100 nM, 60 µM dimethyl histone $H3K4_{1-21}$ peptide substrate).

Panel/Cell Line	Growth Percent
Leukemia	
HL-60(TB)	3.47
K-562	2.70
MOLT-4	5.45
SR	-3.45
Non-Small Cell Lung Cancer	
A549/ATCC	-6.78
EKVX	-25.49
NCI-H226	0.53
NCI-H23	-9.16
NCI-H460	-12.35
NCI-H522	62.46
Colon Cancer	
COLO 205	33.76
HCC-2998	8.53
HCT-116	19.38
HCT-15	-59.36
HT29	18.68
KM12	-17.09
SW-620	13.14
CNS Cancer	
SF-268	-4.53
SF-295	5.97
SF-539	-2.82
SNB-19	-21.60
SNB-75	-38.30
U251	23.41
Melanoma	
LOX IMVI	15.29
MALME-3M	6.40
M14	1.06
MDA-MB-435	10.02
SK-MEL-2	32.52
SK-MEL-28	10.11
SK-MEL-5	69.13
UACC-257	32.02
UACC-62	52.87
Ovarian Cancer	
IGROV1	-16.23
OVCAR-3	2.62
OVCAR-4	-21.60
OVCAR-5	-5.00
OVCAR-8	10.72
NCI/ADR-RES	-70.52
SK-OV-3	-4.32
Renal Cancer	
786-0	-10.75
ACHN	-23.97
CAKI-1	-68.10
RXF 393	7.28
SN12C	-15.71
TK-10	19.46
UO-31	-34.74
Prostate Cancer	
DU-145	-10.66
Breast Cancer	
MCF7	10.38
MDA-MB-231/ATCC	-19.49
HS 578T	-8.59
BT-549	-19.39
T-47D	20.21
MDA-MB-468	32.47
Range	28.06
Delta	69.13
Mean	139.65

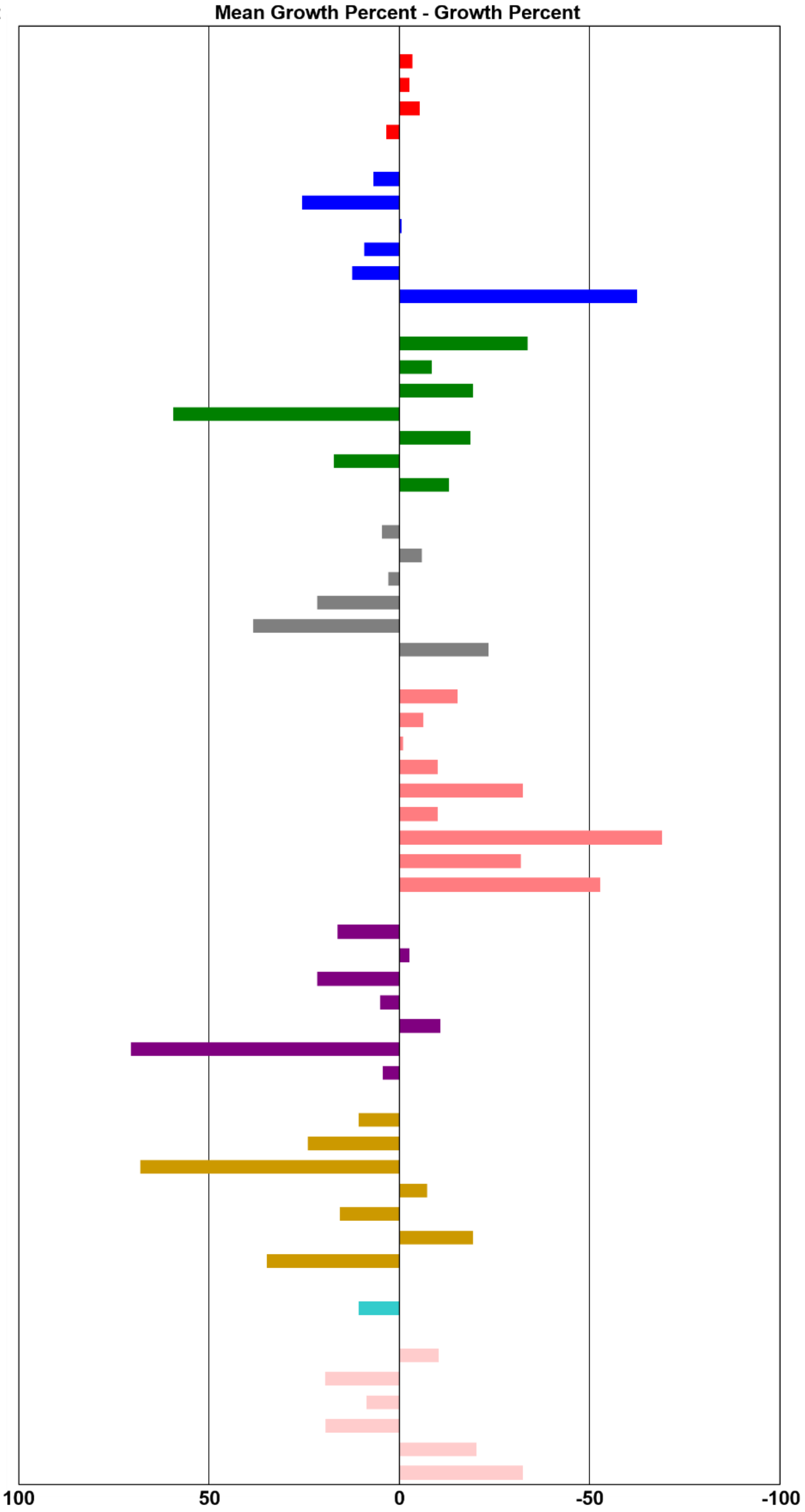

Supplementary Fig. 18 Corin (10 µM) significantly inhibited a variety of melanoma cells lines after 48 h treatment in the NCI-60 Human Tumor Cell Line Screen.

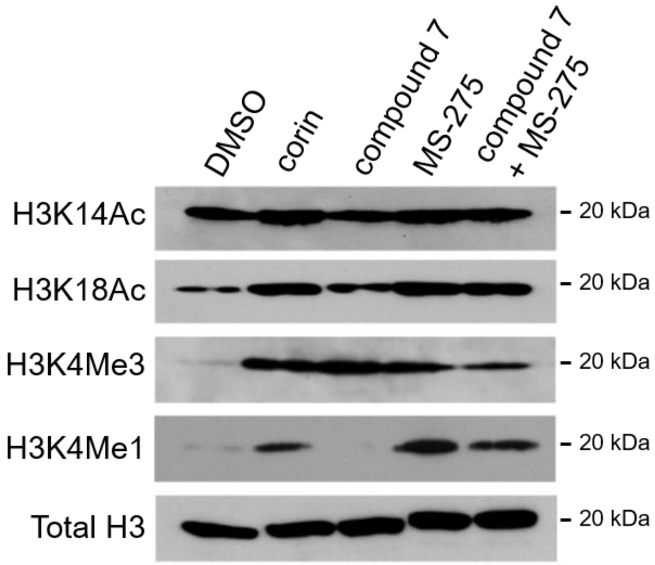

Supplementary Fig. 19 Western blot depicting various histone mark changes in WM983B cells induced by inhibitor treatment. Cells were treated for 24 h with 10 µM compound. Data are representative of two biological replicates.

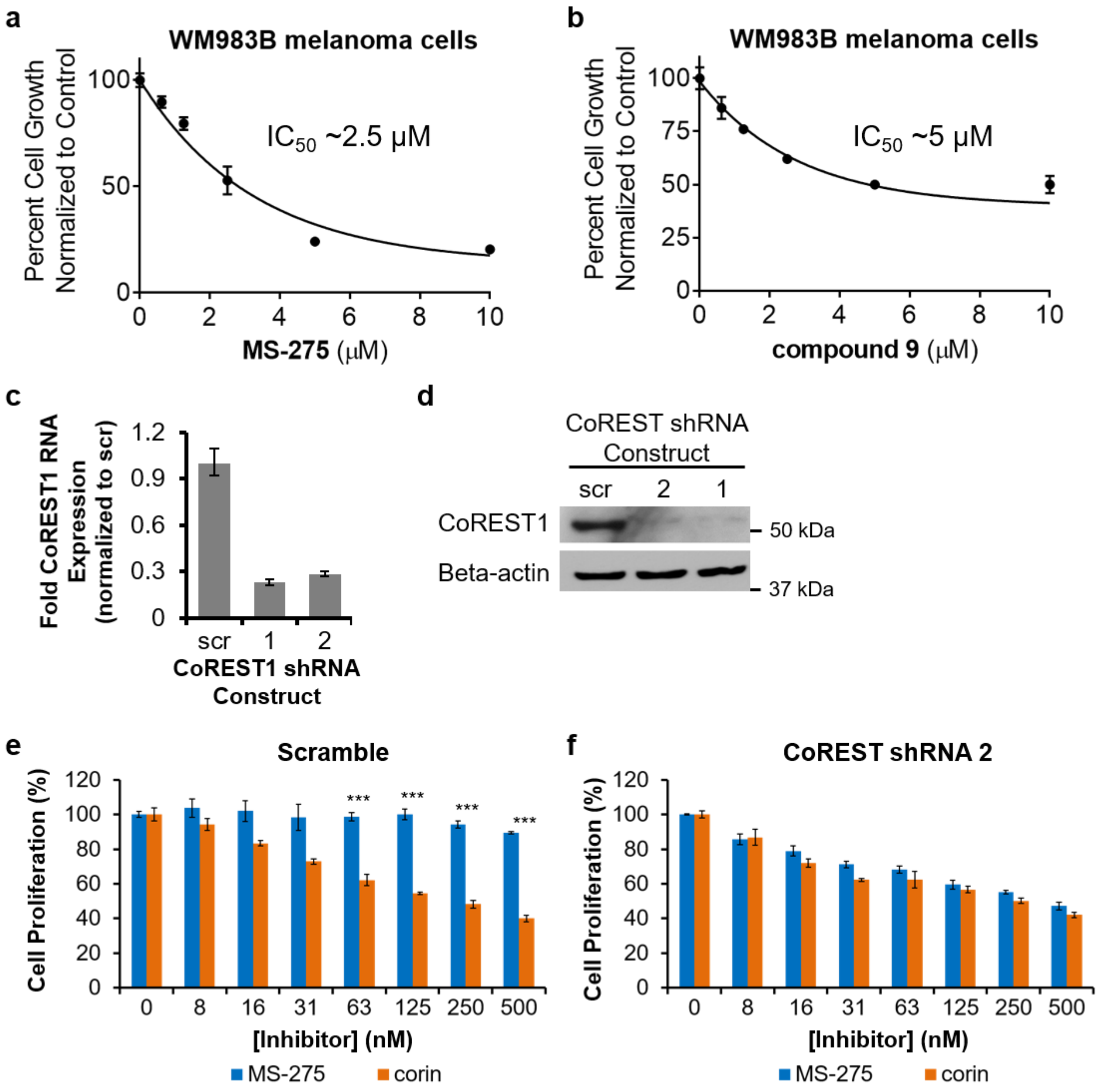

Supplementary Fig. 20 MS-275 and control HDACi, compound **9**, are similarly potent in WM983B melanoma cells. Knockdown of CoREST1 using an alternative shRNA construct reproduces the sensitization effects observed with CoREST shRNA 1. **a**, **b** MS-275 (IC_{50} ~2.5 μM) and compound **9** (IC_{50} ~5 μM) inhibited WM983B melanoma cell growth after 72 h treatment with similar potency. **c** Knockdown efficiency of two shRNA constructs toward CoREST1 as determined by qRT-PCR. **d** Western blot confirming CoREST1 knockdown by two different shRNA constructs in WM983B melanoma cells. **e**, **f** Knockdown of CoREST1 enhances the potency of MS-275 but does not affect the potency of corin as determined after 72 h treatment. Note that cell proliferation was determined using the PicoGreen® cell proliferation assay and data was normalized to zero inhibitor concentration for individual cell lines (scrambled shRNA and CoREST shRNA1). Data (mean ± SE) are representative of at least two independent experiments (unpaired *t* test, ***$p < 0.001$).

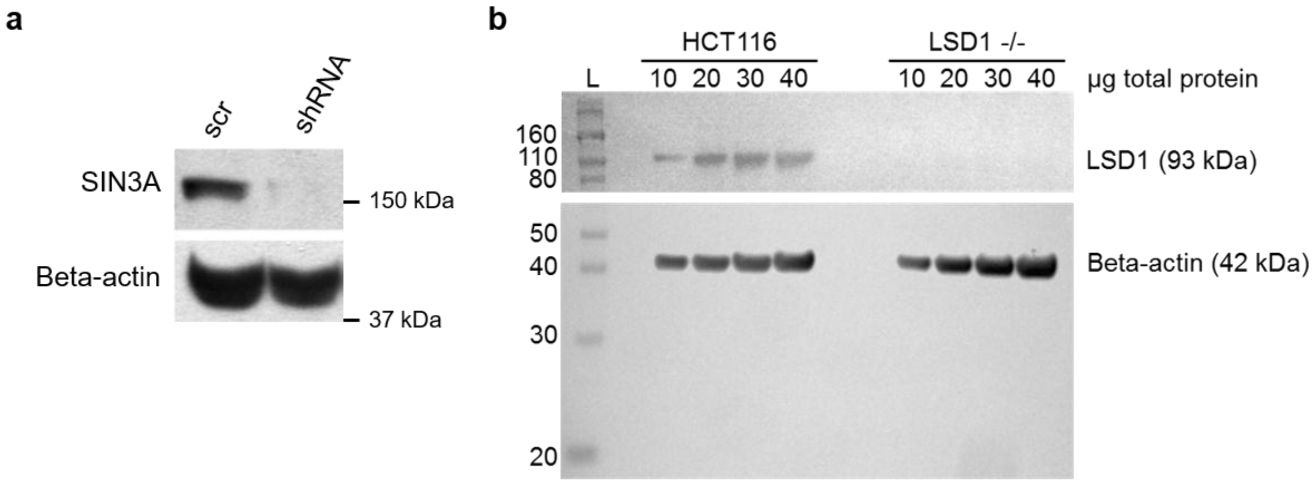

Supplementary Fig. 21 Analysis of target protein expression levels. **a** Western blot confirming shRNA knockdown of the SIN3A corepressor in WM983B melanoma cells. **b** Western blot depicting LSD1 expression in parental and LSD1-/- HCT116 cells. Data are representative of at least two biological replicates.

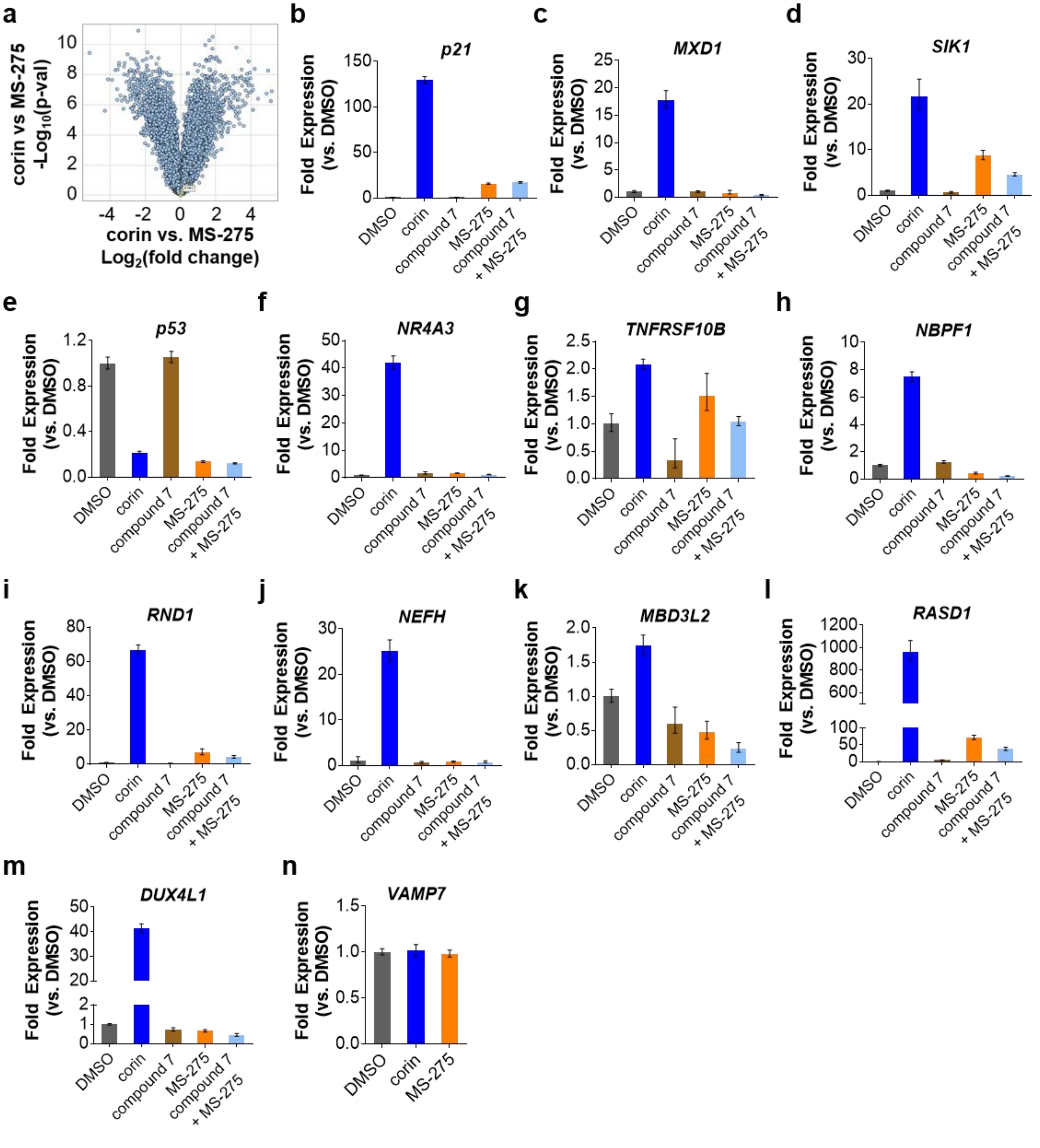

Supplementary Fig. 22 Additional qRT-PCR validation of gene expression changes identified via microarray. **a** Volcano plot suggesting that a unique set of genes was altered by corin treatment as compared to MS-275. **b**-**n** Gene expression changes observed with inhibitor treatment. WM983B melanoma cells were treated with inhibitor (2.5 µM) for 24 h prior to RNA isolation and analysis. Data (mean ± SE) are representative of three independent experiments.

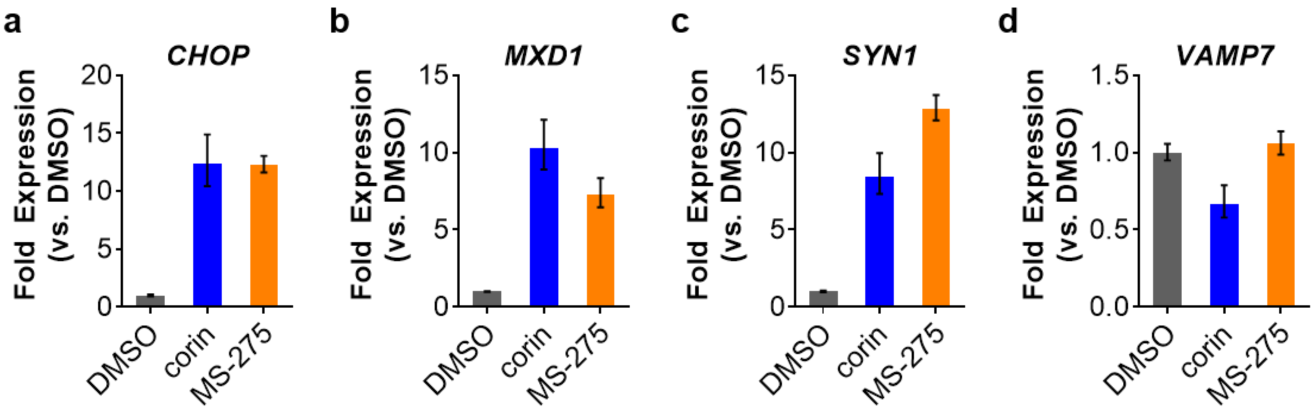

Supplementary Fig. 23 Analysis of gene expression changes in CoREST knockdown WM983B melanoma cells. Cells were treated with inhibitor (2.5 µM) for 24 h prior to RNA isolation and analysis. Data (mean ± SE) are representative of three independent experiments.

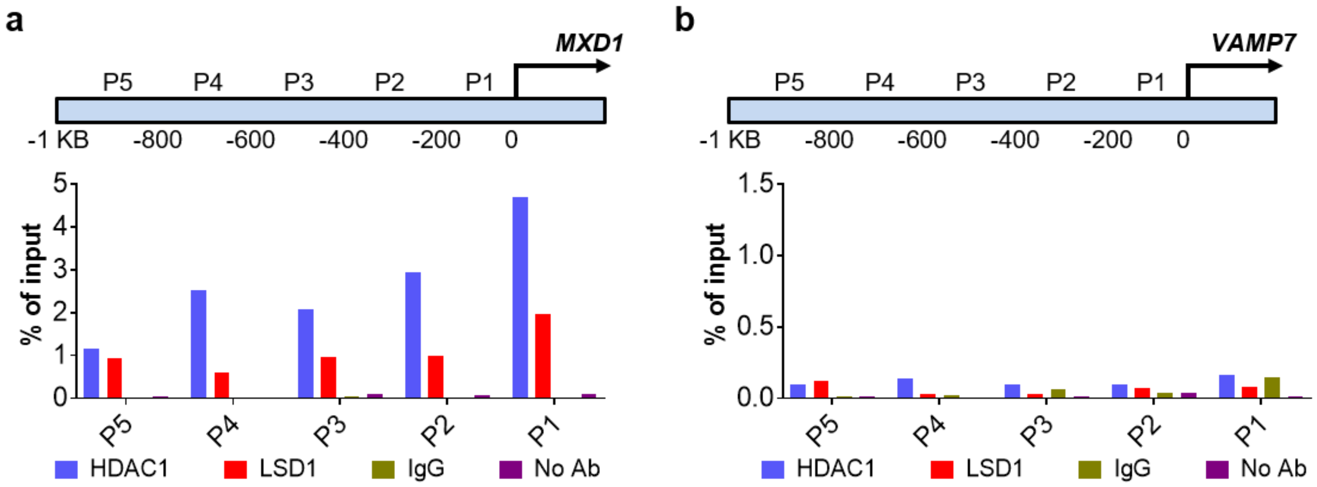

Supplementary Fig. 24 Analysis of HDAC1 and LSD1 localization in the promoter regions of *MXD1* and *VAMP7* by ChIP-PCR. **a** HDAC1 and LSD1, both components of the CoREST complex, were localized to the promoter region of *MXD1*. **b** HDAC1 and LSD1 were not observed in the promoter region of *VAMP7*. WM983B melanoma cells were treated with inhibitor (2.5 µM) for 24 h prior to ChIP-PCR (n=3).

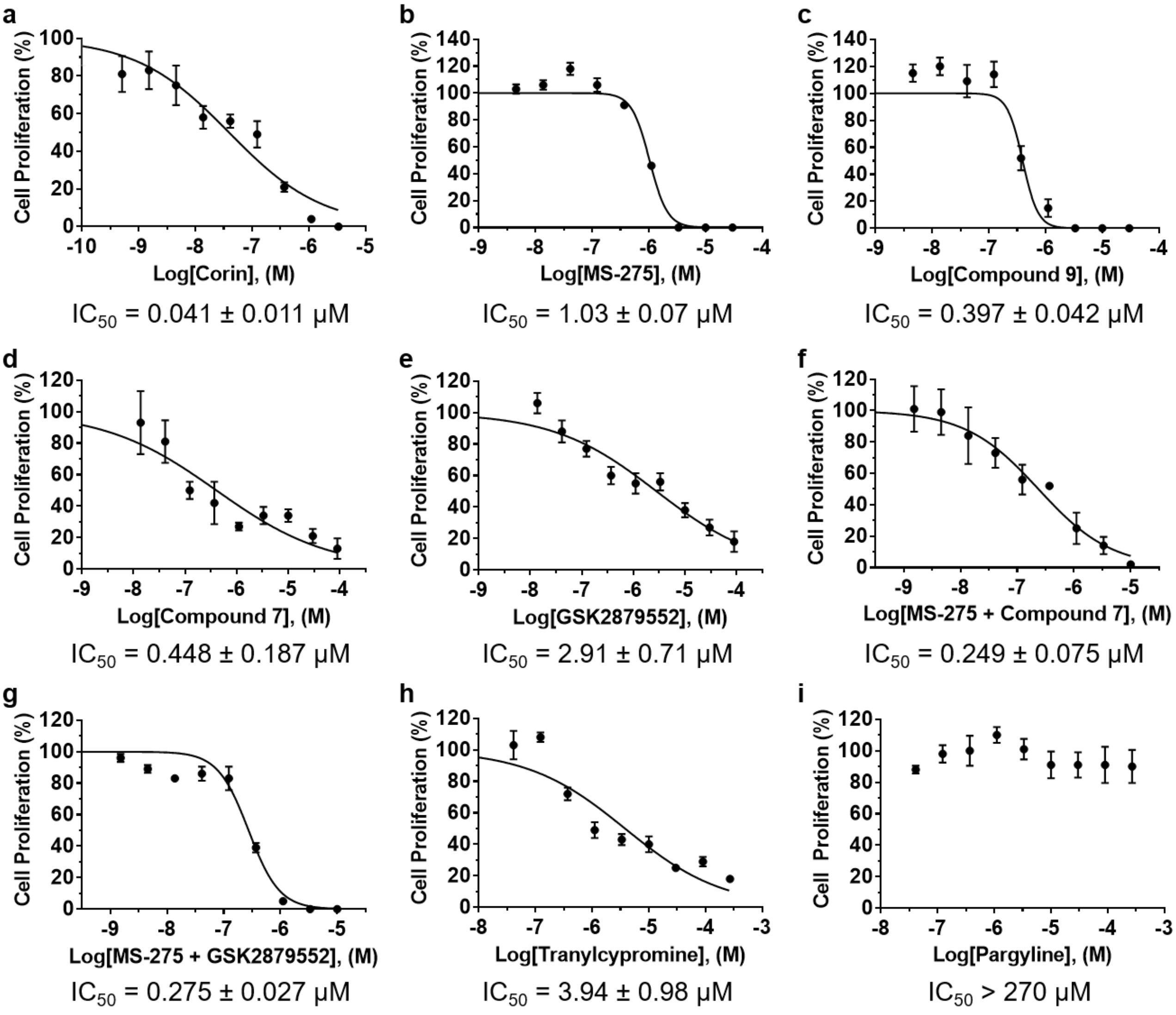

Supplementary Fig. 25 Primary cSCC-IC1 cell proliferation was sensitive to LSD1 inhibitors as well as combinations of LSD1 and HDAC inhibitors as determined by [^{3}H]thymidine incorporation after 72 h treatment. Dose response curve generated for corin (**a**), MS-275 (**b**), compound **9** (**c**), compound **7** (**d**), GSK2879552 (**e**), MS-275 + compound **7** (**f**), MS-275 + GSK2879552 (**g**), tranylcypromine (**h**), pargyline (**i**). Data (mean ± SE) are representative of at least three independent experiments.

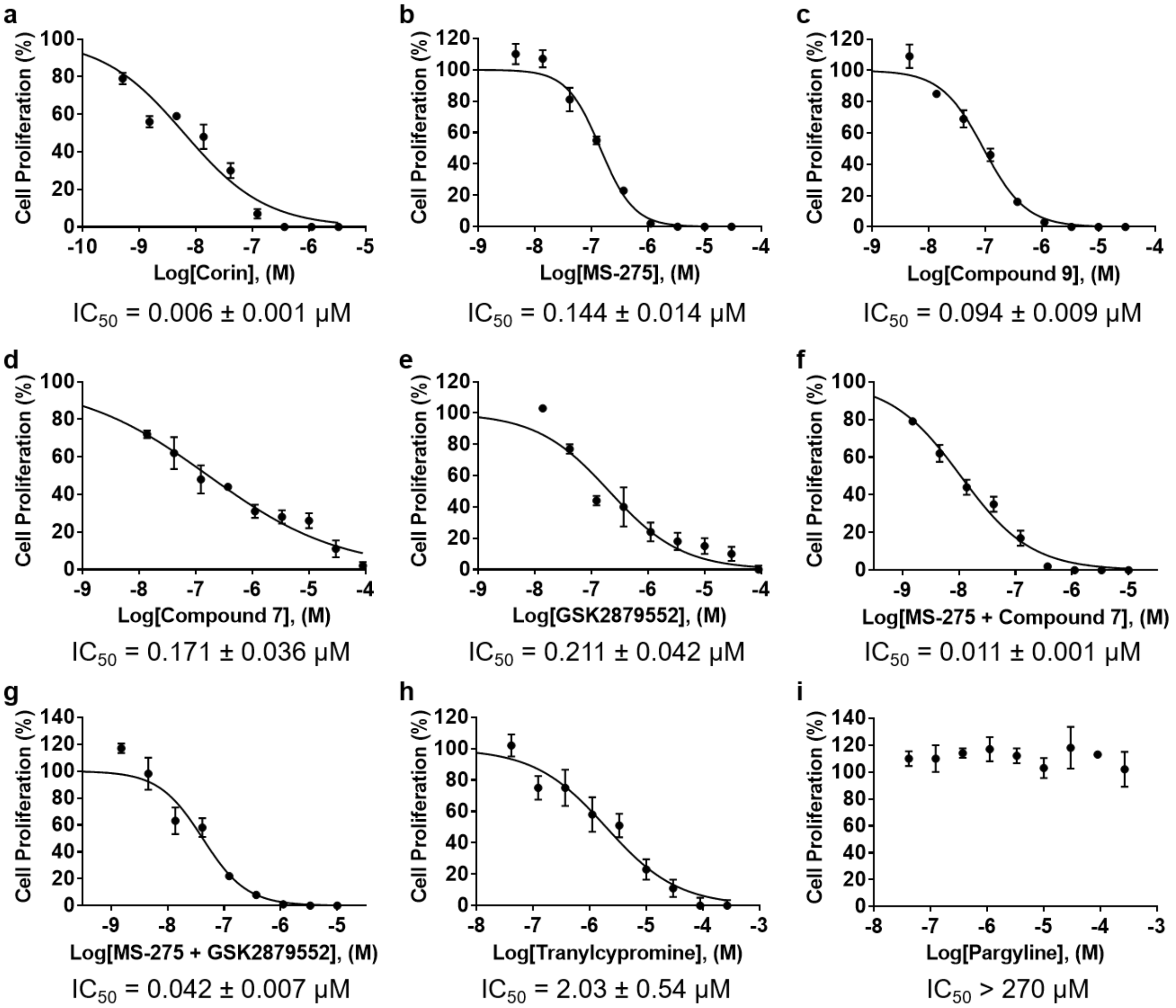

Supplementary Fig. 26 Primary cSCC-MET1 cell proliferation was sensitive to LSD1 inhibitors as well as combinations of LSD1 and HDAC inhibitors as determined by [^{3}H]thymidine incorporation after 72 h treatment. Dose response curve generated for corin (**a**), MS-275 (**b**), compound **9** (**c**), compound **7** (**d**), GSK2879552 (**e**), MS-275 + compound **7** (**f**), MS-275 + GSK2879552 (**g**), tranylcypromine (**h**), pargyline (**i**). Data (mean ± SE) are representative of at least three independent experiments.

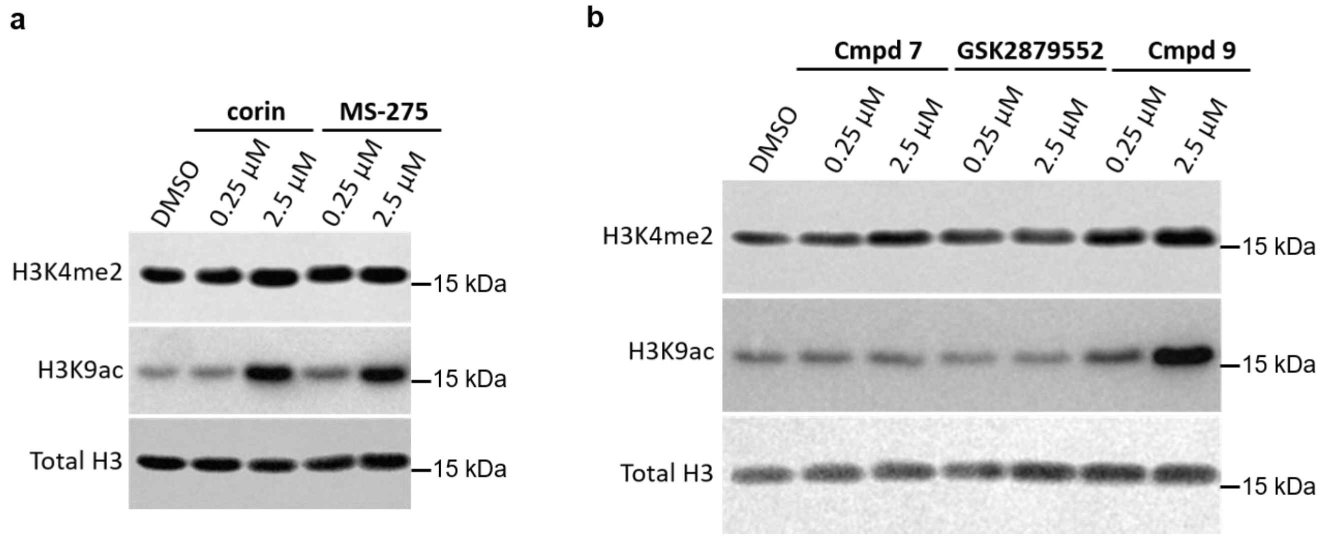

Supplementary Fig. 27 Western blot depicting histone mark changes in SCC-IC1 cells induced by 24 h inhibitor treatment. **a** Both the dual inhibitor corin and the HDACi MS-275 induced increases in global H3K4 dimethylation and H3K9 acetylation after 24 h treatment as determined by Western blot. **b** Monofunctional LSD1 inhibitors compound **7** and GSK2879552 induced modest increases in global H3K4 dimethylation at 2.5 µM but had no effect on H3K9 acetylation after 24 h treatment whereas HDACi compound **9** induced histone mark changes similar to MS-275. Histones were extracted prior to gel loading and transfer to nitrocellulose membranes. Data are representative of two biological replicates.

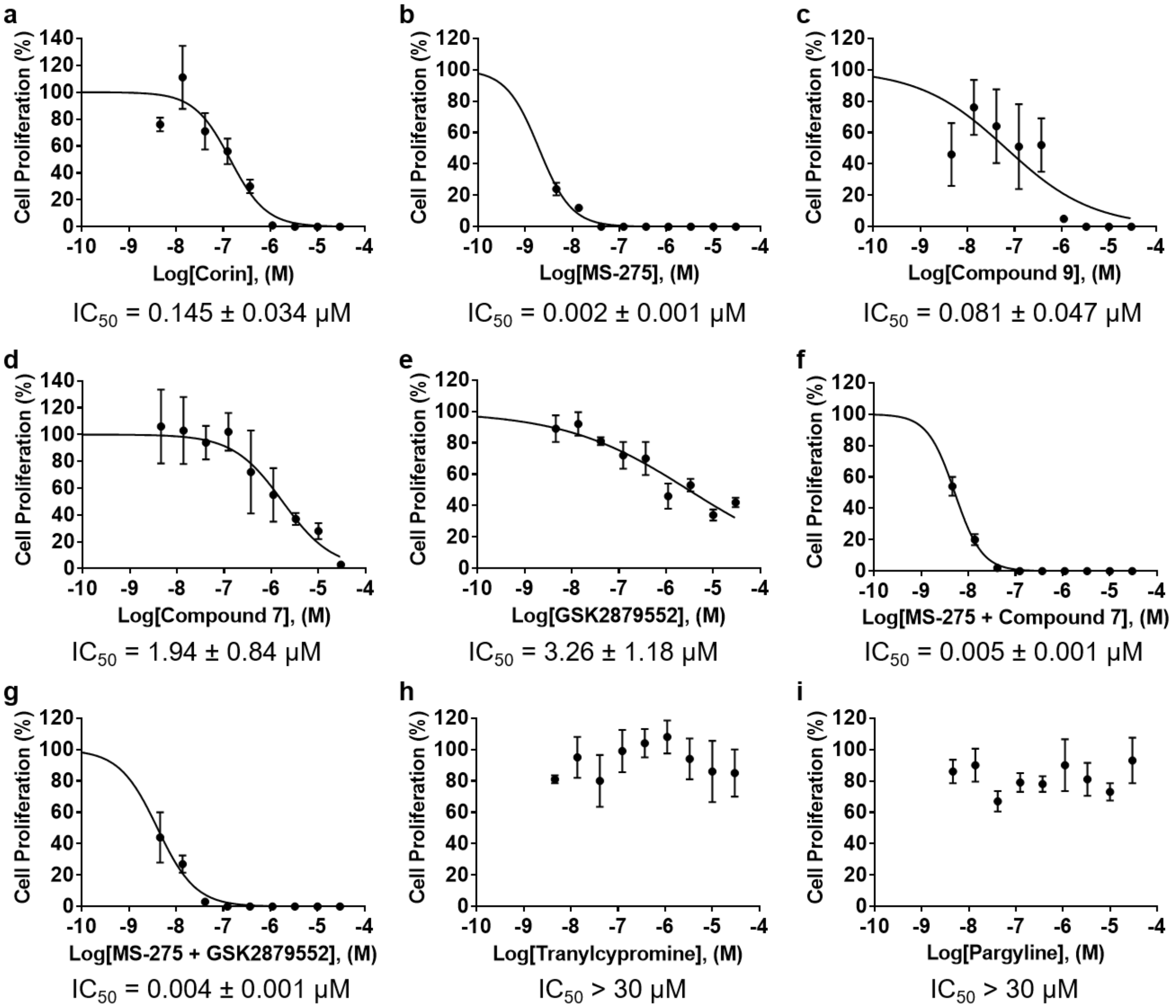

Supplementary Fig. 28 Primary keratinocyte cell proliferation was sensitive to LSD1 inhibitors as well as combinations of LSD1 and HDAC inhibitors as determined by [^{3}H]thymidine incorporation after 72 h treatment. Dose response curve generated for corin (**a**), MS-275 (**b**), compound **9** (**c**), compound **7** (**d**), GSK2879552 (**e**), MS-275 + compound **7** (**f**), MS-275 + GSK2879552 (**g**), tranylcypromine (**h**), pargyline (**i**). Data (mean ± SE) are representative of at least three independent experiments.

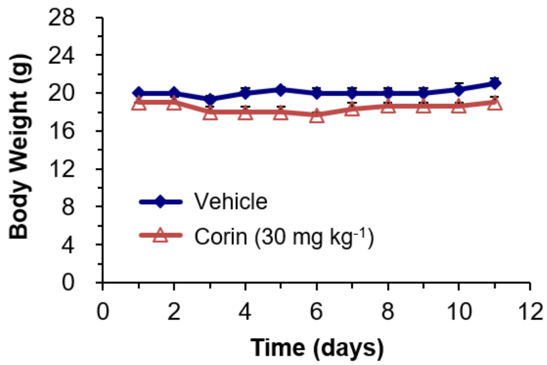

Supplementary Fig. 29 Effect of daily IP corin (30 mg kg^{-1}) on healthy BALB/c nude mice. Corin vs. vehicle effects on mouse body weight over the course of 10 days (n = 3 for vehicle and corin treated). Data are reported as the mean ± SE.

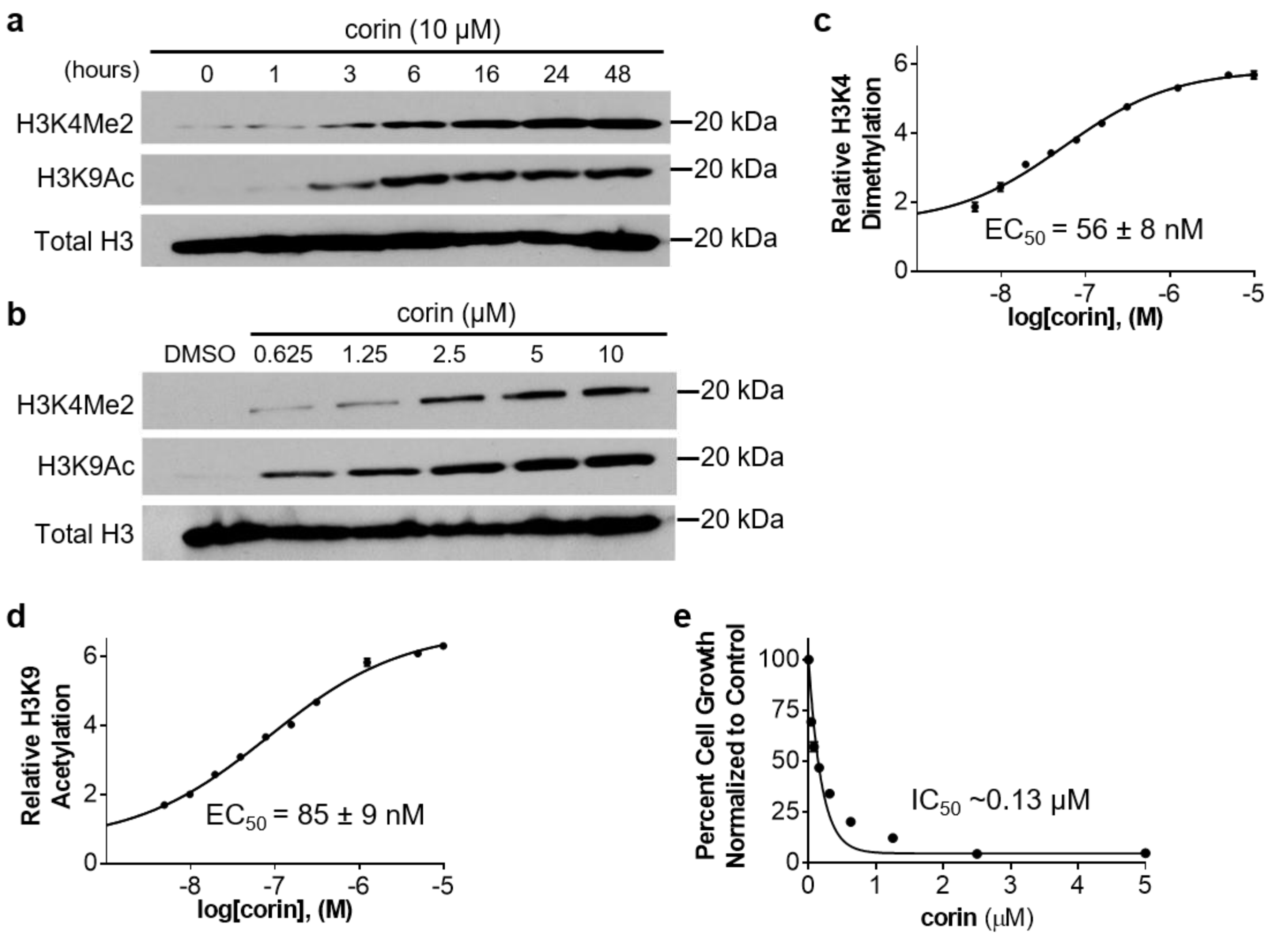

Supplementary Fig. 30 Corin inhibited the growth of SK-MEL-5 melanoma cells and induced dose and time dependent increases in histone methylation and acetylation in the same cell line. **a** Western blot illustrating time dependent increases in histone acetylation and methylation induced by 10 µM corin. **b** Western blot illustrating a dose dependent response induced by corin after 24 h treatment. **c**, **d** Corin induced H3K4 methylation (EC_{50} = 56 ± 8 nM) and H3K9 acetylation (EC_{50} = 85 ± 9 nM) in SK-MEL-5 melanoma cells as determined by ELISA after 24 h inhibitor treatment. **e** Corin (IC_{50} ~0.13 µM) significantly inhibited the growth of SK-MEL-5 cells after 72 h treatment as determined using a PicoGreen® cell proliferation assay. Data (mean ± SE) are representative of two independent experiments.

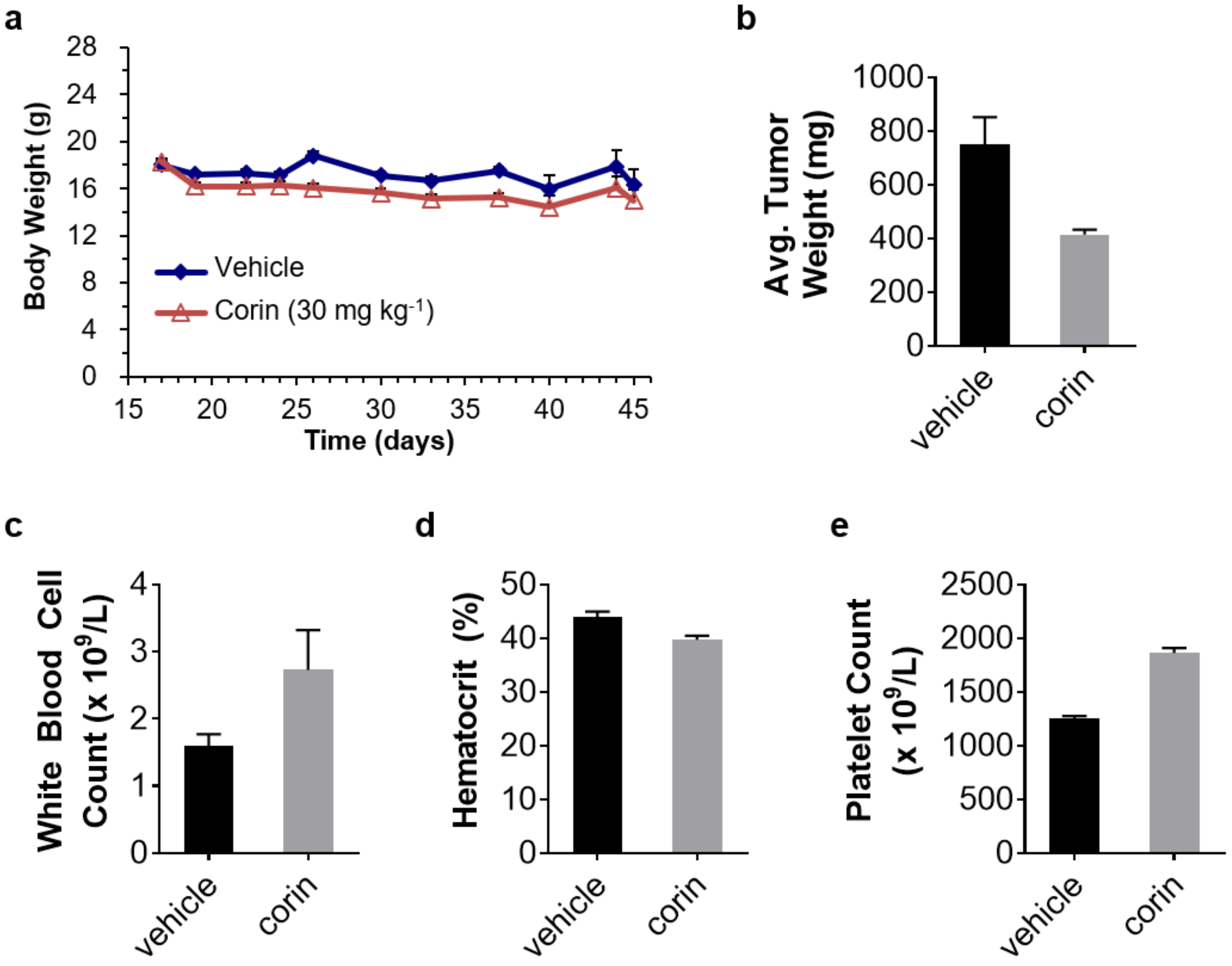

Supplementary Fig. 31 Effects of daily IP corin (30 mg kg^{-1}) on SK-MEL-5 xenograft mice. **a** Corin vs. vehicle effects on mouse body weight over the course of the 28-day xenograft experiment (n = 10 for vehicle and corin treated). **b** Size of the excised tumors in vehicle vs. corin treated mice (n = 10 for vehicle and corin treated) after 28 days. **c**, **d**, **e** Effects of corin vs. vehicle on white blood cell count, hematocrit, and platelet counts after 28 days (n = 3 for vehicle and corin treated). Data (mean ± SE) are representative of at least two independent experiments.

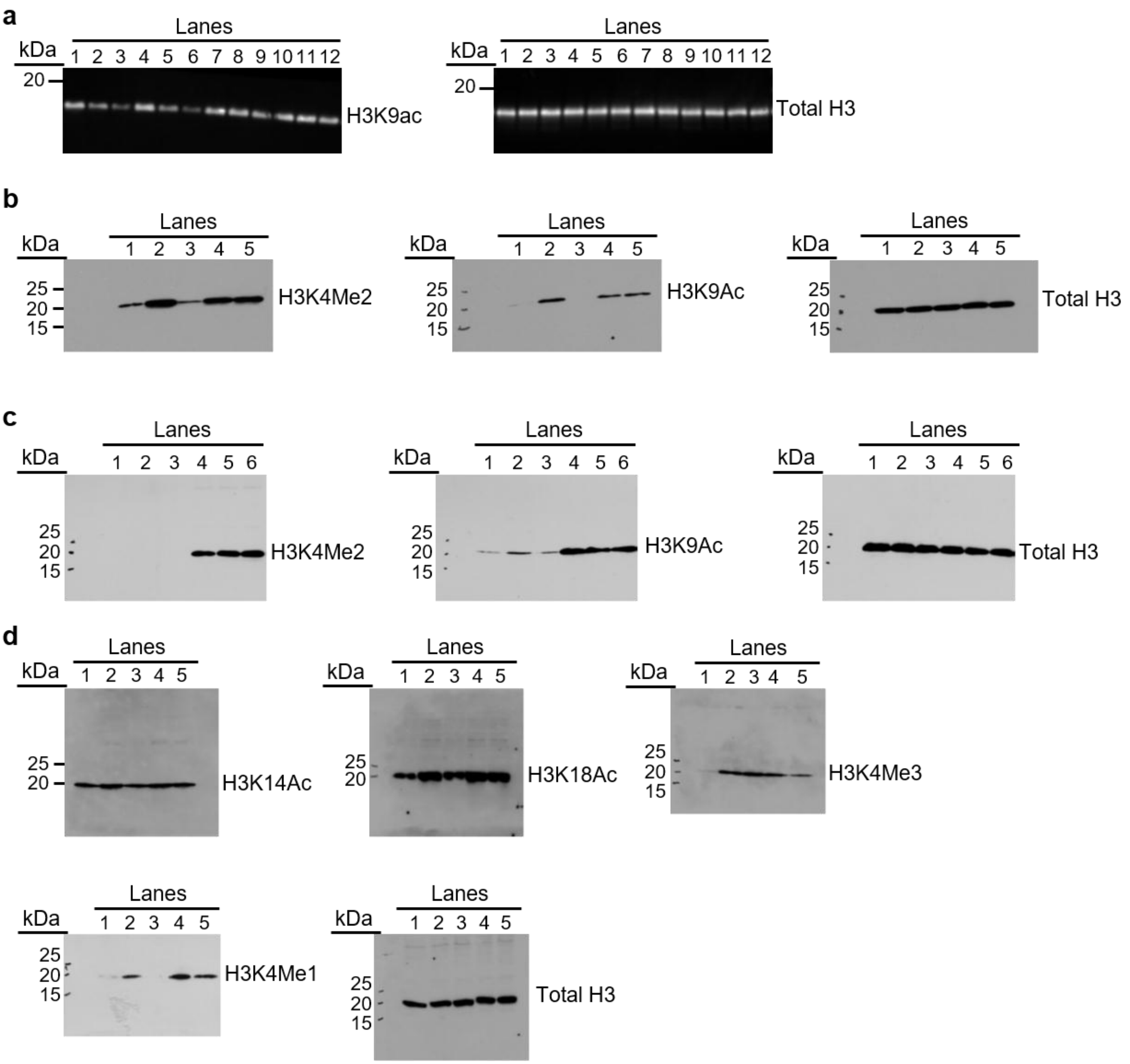

Supplementary Fig. 32 Uncropped Western blots. **a** Fig. 2d, lane 1: untreated (0 h), lane 2: untreated (1 h), lane 3: untreated (2 h), lane 4: vehicle (0 h), lane 5: vehicle (1 h), lane 6: vehicle (2 h), lane 7: 1 µM corin (0 h), lane 8: 1 µM corin (1 h), lane 9: 1 µM corin (2 h), lane 10: 10 µM corin (0 h), lane 11: 10 µM corin (1 h), lane 12: 10 µM corin (2 h). **b** Fig. 4a, lane 1: DMSO, lane 2: corin, lane 3: compound **7**, lane 4: MS-275, lane 5: MS-275 + compound **7**. **c** Fig. 6b, lane 1: mouse 1 (DMSO), lane 2: mouse 2 (DMSO), lane 3: mouse 3 (DMSO), lane 4: mouse 1 (corin 30 mg kg^{-1}), lane 5: mouse 2 (corin 30 mg kg^{-1}), lane 6: mouse 3 (corin 30 mg kg^{-1}). **d** Supplementary Fig. 19, lane 1: DMSO, lane 2: corin, lane 3: compound **7**, lane 4: MS-275, lane 5: MS-275 + compound **7**. For panels **b**, **c**, and **d**, molecular weight markings were drawn on the film after aligning with membrane.

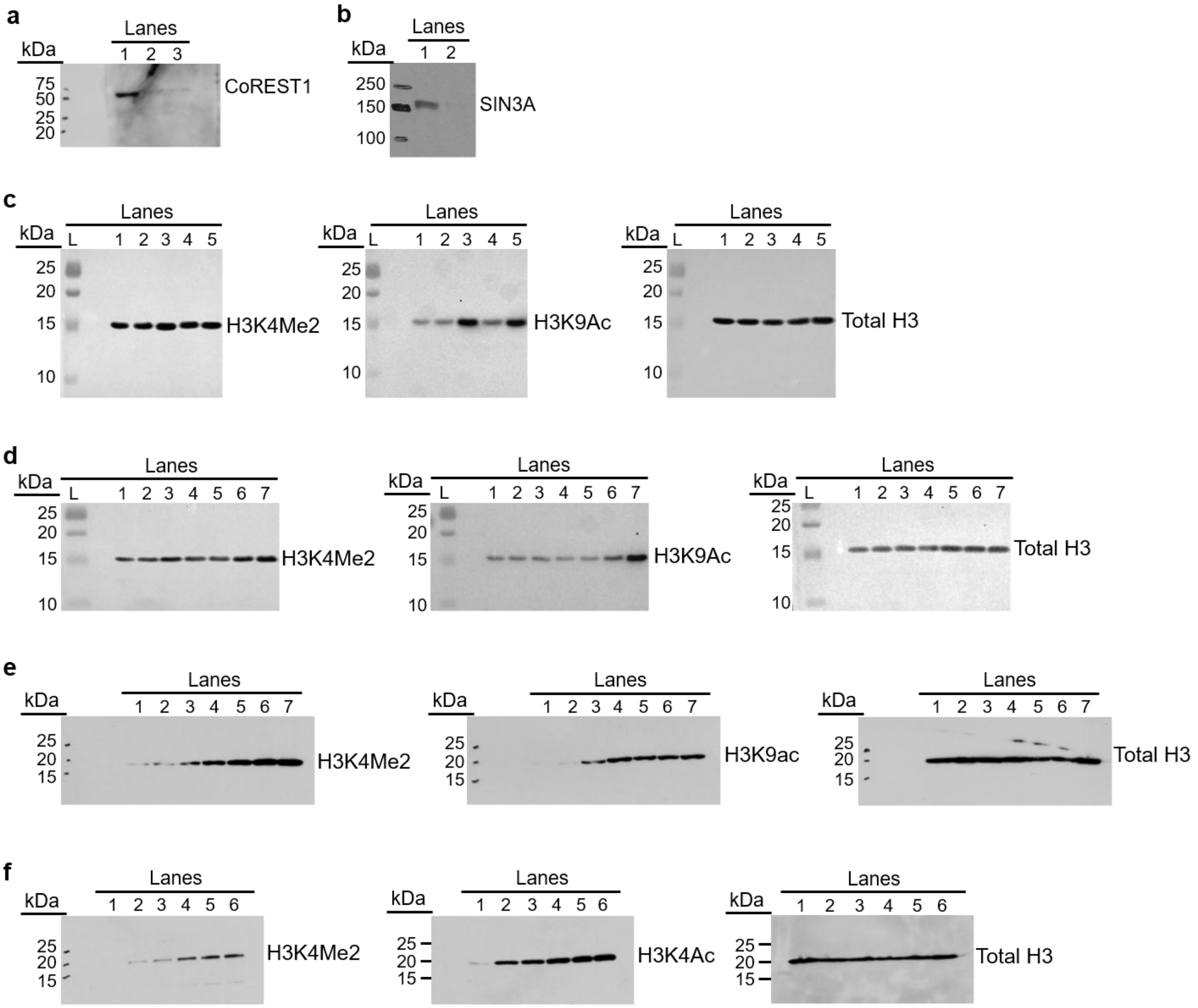

Supplementary Fig. 33 Uncropped Western blots continued. **a** Supplementary Fig. 20d, lane 1: WM983B cells, scr shRNA, lane 2: WM983B cells, CoREST1 shRNA 2, lane 3: WM983B cells, CoREST1 shRNA 1. **b** Supplementary Fig. 21a, lane 1: WM983B cells, scr shRNA, lane 2: WM983B cells, SIN3A shRNA. **c** Supplementary Fig. 27a, lane 1: DMSO, lane 2: 0.25 µM corin, lane 3: 2.5 µM corin, lane 4: 0.25 µM MS-275, lane 5: 2.5 µM MS-275. **d** Supplementary Fig. 27b, lane 1: DMSO, lane 2: 0.25 µM compound **7**, lane 3: 2.5 µM compound **7**, lane 4: 0.25 µM GSK2879552, lane 5: 2.5 µM GSK2879552, lane 6: 0.25 µM compound **9**, lane 7: 2.5 µM compound **9**. **e** Supplementary Fig. 30a, lane 1: 10 µM corin (0 h), lane 2: 10 µM corin (1 h), lane 3: 10 µM corin (3 h), lane 4: 10 µM corin (6 h), lane 5: 10 µM corin (16 h), lane 6: 10 µM corin (24 h), lane 7: 10 µM corin (48 h). **f** Supplementary Fig. 30b, lane 1: DMSO, lane 2: 0.625 µM corin, lane 3: 1.25 µM corin, lane 4: 2.5 µM corin, lane 5: 5 µM corin, lane 6: 10 µM corin. For panels **a**, **b**, **e**, and **f**, molecular weight markings were drawn on the film after aligning with membrane.

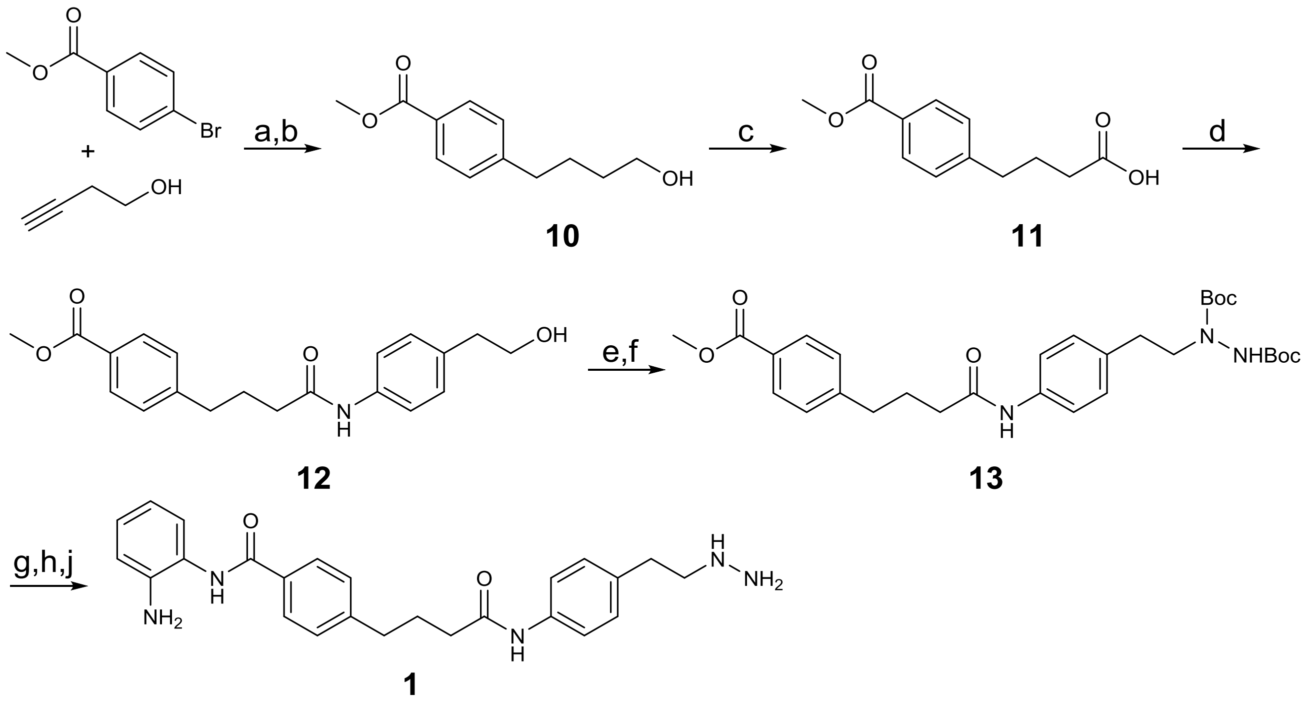

Supplementary Fig. 34 Synthetic route for the preparation of compound **1**. Reagents and conditions: a) $PdCl_2$, PPh_3, Et_2NH, CuI, RT, 24 h; b) Pd/C, H_2 (60 psi), EtOH, RT, 12 h; c) i) PCC, NaOAc, DCM, RT, 12 h; ii) $NaClO_2$, $NaH_2PO_4 \cdot H_2O$, H_2O_2, H_2O, MeCN, 0 °C, 2 h; d) 2-(4-aminophenyl)ethanol, HATU, Et_3N, DCM, 0 °C → RT, 16 h; e) PPh_3, CBr_4, DCM, RT, 30 min; f) BocNHNHBoc, NaH, DMF, -40 °C, 3 h; g) LiOH, THF, MeOH, H_2O, RT, 16 h; h) **15**, HATU, Et_3N, DCM, 0 °C → RT, 6 h; j) TFA, DCM, RT, 6 h.

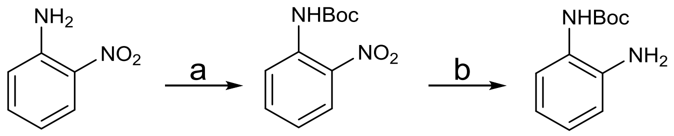

14 **15**

Supplementary Fig. 35 Preparation of the protected *o*-phenylenediamine intermediate **15**. Reagents and conditions: a) Boc_2O, NaHMDS, THF, RT, 2 h; b) H_2, Pd/C, MeOH, RT, 16 h.

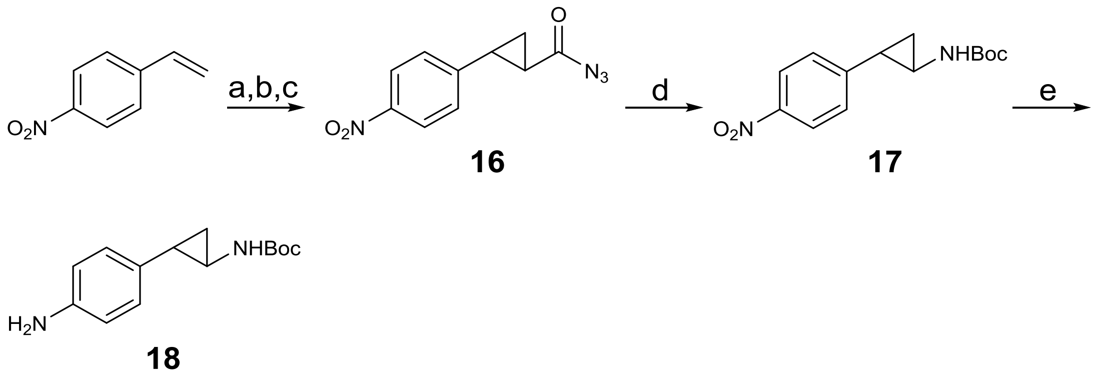

Supplementary Fig. 36 Synthesis of aryl amine intermediate **18**. Reagents and conditions: a) dirhodium tetraacetate, ethyl diazoacetate, DCM, RT, 16 h; b) LiOH, THF, MeOH, H_2O, 0 ºC, 4 h; c) i) $SOCl_2$, 0 ºC → reflux, 1 h; ii) NaN_3, acetone, H_2O, 0 ºC, 30 min; d) i) toluene, 100 ºC, 2 h; ii) ^{t}BuOH, reflux, 2 h; e) K_2CO_3, Pd/C, $NaPO_2H_2 \cdot H_2O$, THF, H_2O, 60 ºC, 2 h.

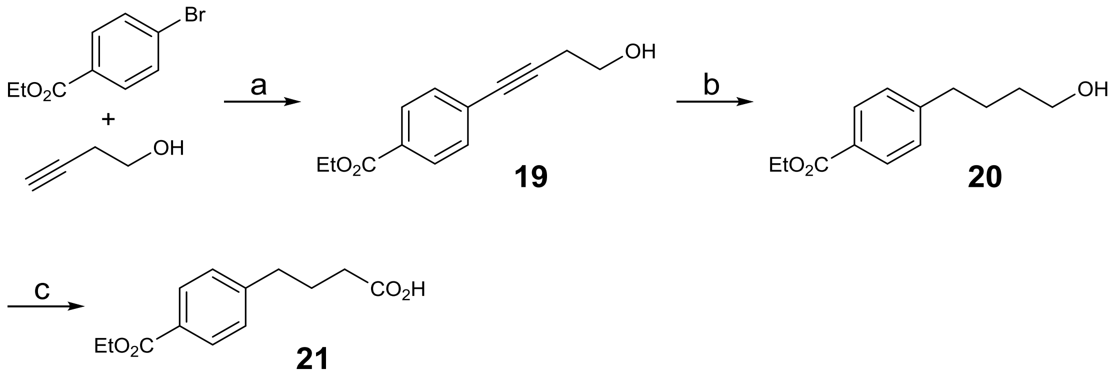

Supplementary Fig. 37 Synthesis of alkyl acid intermediate **21**. Reagents and conditions: a) $PdCl_2$, PPh_3, Et_2NH, CuI, DCM, RT, 1 h; b) Pd/C, H_2 (80 psi), EtOH, RT, 24 h; c) i) PCC, NaOAc, DCM, RT, 4 h; ii) $NaClO_2$, $NaH_2PO_4{\cdot}H_2O$, H_2O_2, H_2O, MeCN, 0 °C, 2.5 h.

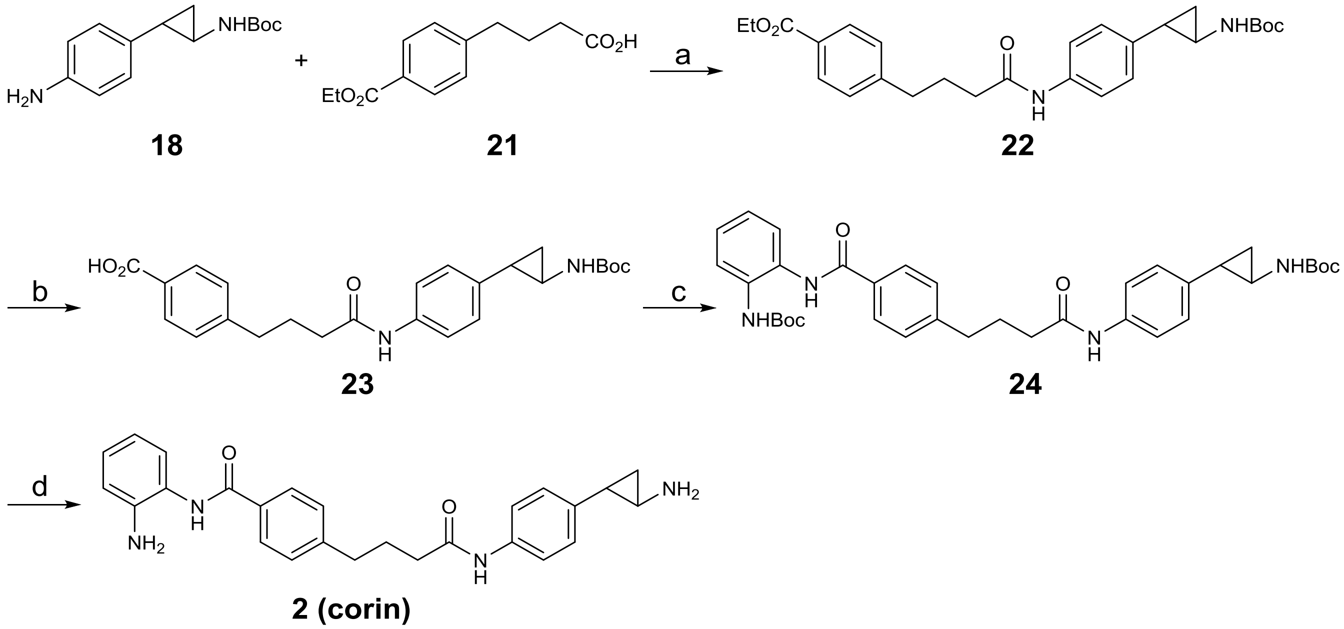

Supplementary Fig. 38 Synthetic route for the preparation of compound **2**, corin. Reagents and conditions: a) HATU, DIEA, DCM, 0 ºC → RT, 4 h; b) LiOH, THF, MeOH, H_2O, 0 ºC → RT, 4 h; c) **15**, HATU, DIEA, DCM, 0 ºC → RT, 4 h; d) PhSH, TFA, DCM, 0 ºC → RT, 2 h.

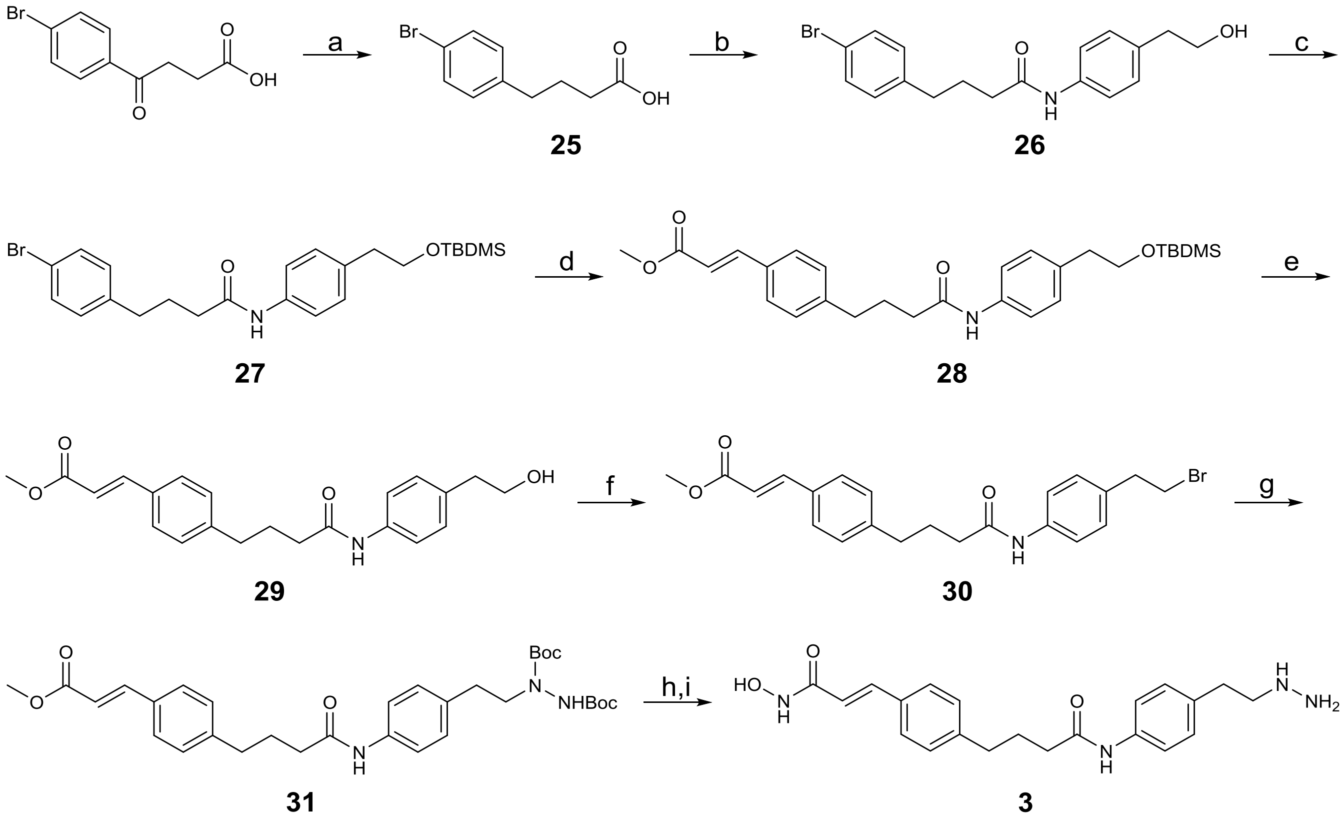

Supplementary Fig. 39 Synthesis of the LBH589/bizine dual functional inhibitor **3**. Reagents and conditions: a) N_2H_4, KOH, diethylene glycol, 120 °C → 200 °C, 5 h; b) 2-(4-aminophenyl)ethanol, HATU, Et_3N, DCM, 0 °C → RT, 4 h; c) TBDMSCl, DMAP, Et_3N, DCM, RT, 2 h; d) $Pd(OAc)_2$, PPh_3, TMED, methyl acrylate, toluene, 130 °C, 48 h; e) TBAF, THF, RT, 16 h; f) PPh_3, CBr_4, DCM, RT, 30 min; g) BocNHNHBoc, NaH, DMF, -40 °C, 3 h; h) NH_2OH (aq), NaOH, THF, MeOH, 0 °C → RT, 30 min; i) TFA, DCM, RT, 16 h.

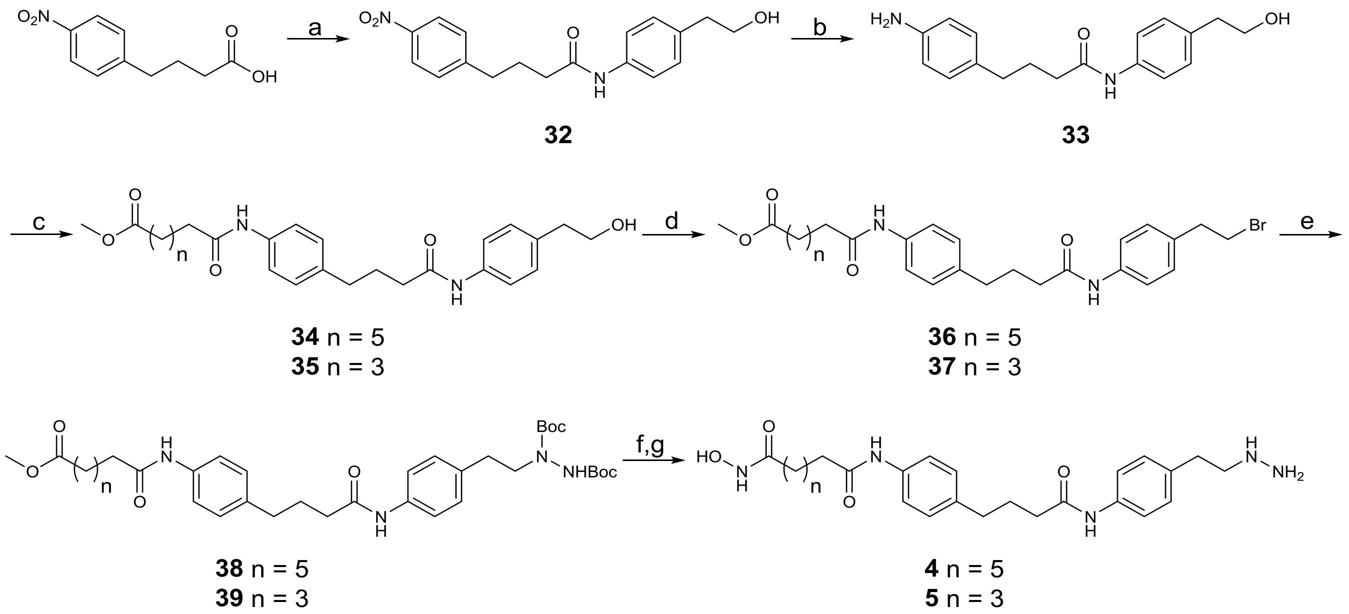

Supplementary Fig. 40 Synthetic scheme for the preparation of the SAHA/bizine dual inhibitors, compounds **4** and **5**. Reagents and conditions: a) 2-(4-aminophenyl)ethanol, HATU, Et_3N, DCM, 0 °C → RT, 4 h; b) H_2, Pd/C, AcOH, EtOH, RT, 16 h; c) suberic/adipic acid monomethyl ester, HATU, Et_3N, DCM, DMF, RT, 4 h; d) PPh_3, CBr_4, DCM, RT, 30 min; e) BocNHNHBoc, NaH, DMF, -40 °C, 16 h; f) NH_2OH (aq), NaOH, THF, MeOH, 0 °C → RT, 30 min; g) TFA, DCM, RT, 16 h.

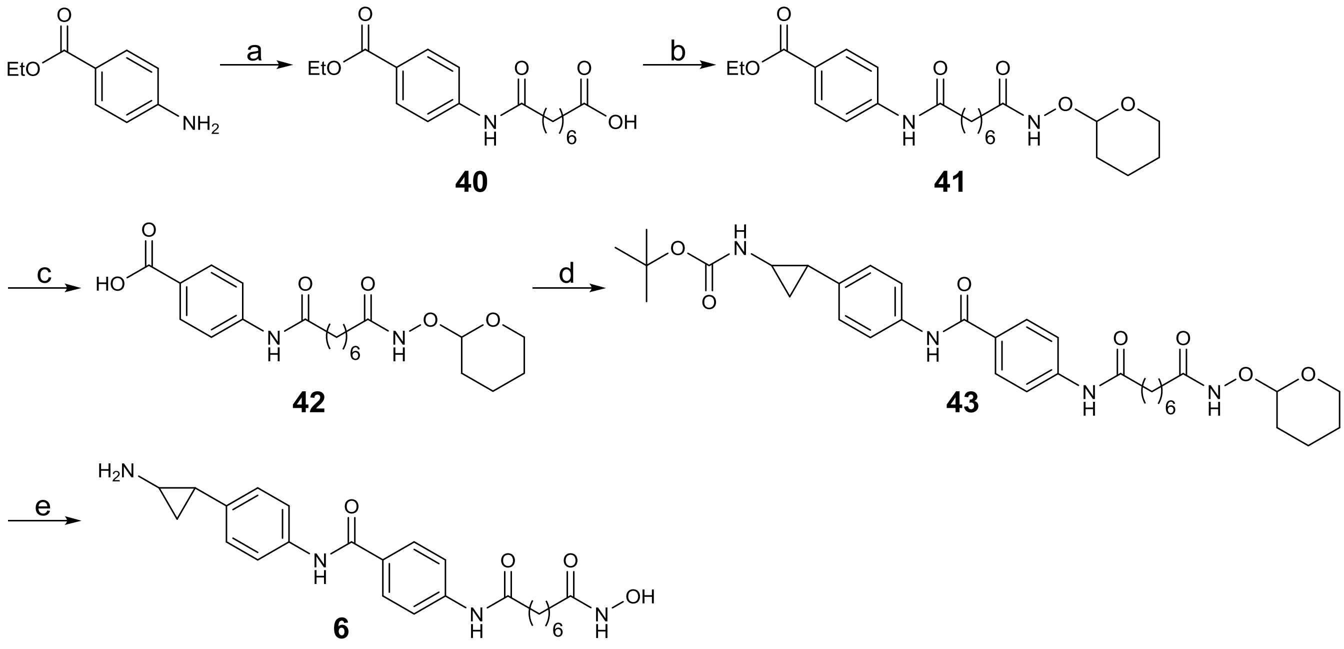

Supplementary Fig. 41 Synthetic scheme for the preparation of the SAHA/tranylcypromine dual inhibitor **6**. Reagents and conditions: a) suberic anhydride, THF, 0 °C, 1.5 h; b) i) $ClCO_2Et$, Et_3N, THF, 0 °C, 30 min; ii) $THPONH_2$, THF, 0 °C, 1.5 h; c) $LiOH{\cdot}H_2O$, THF/H_2O, RT, overnight; d) i) Et_3N, PyBOP, DMF, RT, 45 min; ii) **18**, DMF, RT, overnight; e) 4N HCl, dioxane/THF, RT, overnight.

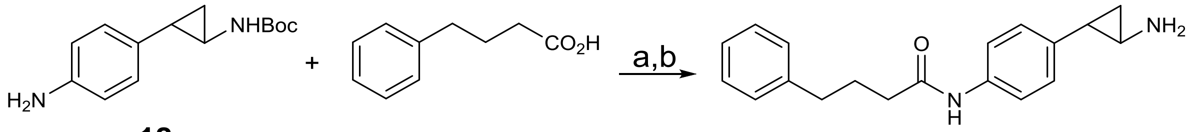

18 **7**

Supplementary Fig. 42 Synthetic scheme for the preparation of the structurally matched, tranylcypromine based LSD1 inhibitor **7**. Reagents and conditions: a) HATU, DIEA, DCM, 0 ºC → RT, 4 h; b) PhSH, TFA, DCM, 0 ºC, 2 h.

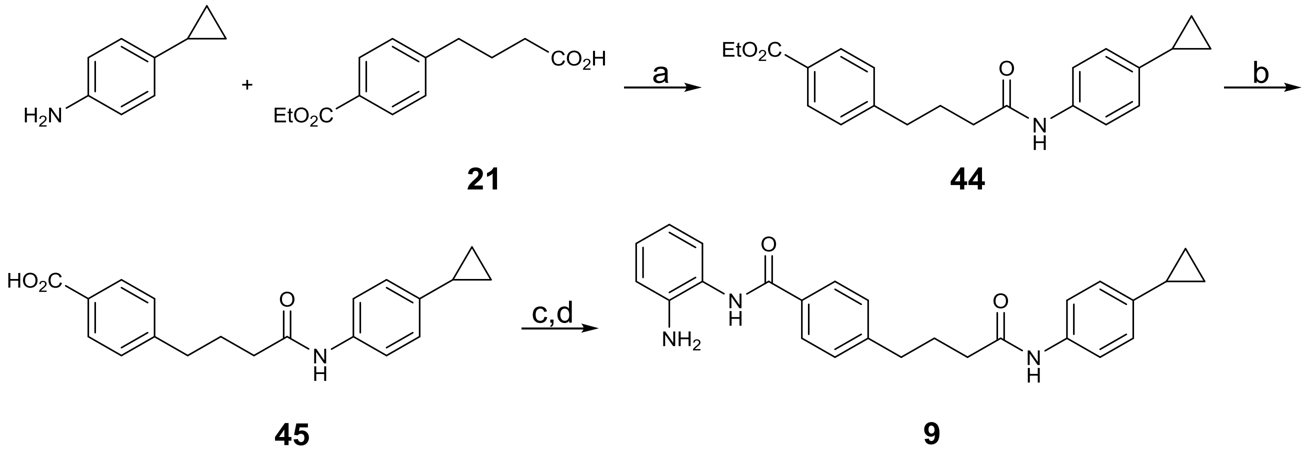

Supplementary Fig. 43 Synthetic route for the preparation of control compound **9**. Reagents and conditions: a) HATU, DIEA, DCM, 0 ºC → RT, 2 h; b) LiOH, THF, MeOH, H_2O, 0 ºC → RT, 4 h; c) **15**, HATU, DIEA, DCM, 0 ºC → RT, 2 h; d) PhSH, TFA, DCM, 0 ºC → RT, 2 h.

Supplementary Table 1 Physiochemical properties calculated for tested inhibitors.

Cmpd ID	MW (g mol^{-1})	cLogP	cLogD (pH 7.4)	tPSA
**1**	431.53	2.08	1.83	122.27
**corin**	428.54	2.19	1.34	110.24
**3**	382.46	2.29	1.04	116.48
**4**	483.60	1.27	2.09	145.58
**5**	455.55	0.51	1.37	145.58
**6**	438.52	0.73	0.47	133.55
**bizine**	297.39	2.63	2.16	67.15
**GSK2879552**	364.49	3.40	1.15	52.57
**7**	294.40	2.74	1.51	55.12
**8**	252.31	1.66	1.01	55.12
**9**	413.52	4.21	4.09	84.22

Calculations were done using software available from ACD Labs (https://ilab.acdlabs.com/iLab2/).

Supplementary Table 2 Counterscreen data illustrating the selectivity of dual inhibitors over other amine oxidase enzymes.

Enzyme	Inhibitor	k_{inact} (min^{-1})	$K_{i(inact)}$ (μM)	$k_{inact}/K_{i(inact)}$ (min^{-1} μM^{-1})	Fold Selectivity for LSD1
LSD2[a]	**1**	~0.28	>20	<0.014	>39
	**2 (corin)**	~0.17	>20	<0.0085	>200
	**3**	~0.20	>20	<0.010	>105
	**4**	~0.25	>20	<0.012	>53
	**5**	~0.25	>20	<0.013	>72
	**6**	ND	>20	ND	ND
	**GSK2879552**	---	---	---	---
	**7**	~0.17	>20	<0.0085	>200
	**8**	ND	>20	ND	ND
	**9**	---	---	---	---
MAO A	**1**[b]	ND	ND	0.055 ± 0.001	10
	**2 (corin)**	0.23 ± 0.04	3.1 ± 1.1	0.074	23
	**3**[c]	ND	ND	~0.06	~18
	**4**[b]	ND	ND	0.063 ± 0.003	10
	**5**[b]	ND	ND	0.062 ± 0.004	15
	**6**	0.056 ± 0.007	0.25 ± 0.09	0.22	~23
	**GSK2879552**	ND	>20	ND	ND
	**7**	0.38 ± 0.10	1.03 ± 0.78	0.37	5
	**8**	0.069 ± 0.003	0.29 ± 0.04	0.24	~21
	**9**	---	---	---	---
MAO B	**1**[b]	ND	ND	0.0087 ± 0.0001	63
	**2 (corin)**	ND	>20	ND	ND
	**3**[c]	ND	ND	~0.02	~53
	**4**[b]	ND	ND	0.0065 ± 0.0006	98
	**5**[b]	ND	ND	0.012 ± 0.001	78
	**6**	ND	>20	ND	ND
	**GSK2879552**	ND	>80	ND	ND
	**7**	ND	ND	0.37 ± 0.03	5
	**8**	ND	>20	ND	ND
	**9**	---	---	---	---

ND indicates that the parameter was not able to be determined accurately. Dash marks indicate that compound was not evaluated in the corresponding assay. Data (k_{inact} or $K_{i(inact)}$ ± SE) are representative of at least two independent experiments. [a]Compounds did not appear to inhibit LSD2 at concentrations up to 20 μM. k_{inact} values for inhibitors against LSD2 were considered comparable to those against LSD1 in order to approximate fold selectivity (inhibitory efficiency toward LSD1/estimated inhibitor efficiency toward LSD2). [b]Plots of the k_{obs} values versus inhibitor concentration appeared linear which precluded use of the Kitz-Wilson equation to determine k_{inact} and apparent $K_{i(inact)}$. Instead, the data were fit to a linear regression with the slope representing $k_{inact}/K_{i(inact)}$. [c]$k_{inact}/K_{i(inact)}$ values were estimated from steady-state progress curves. LSD2 assay: [LSD2] = 430 nM, substrate = 100 μM dimethyl histone H3K4$_{1\text{-}21}$ peptide. MAO A assay: [MAO A] = 200 nM, substrate = 200 μM tyramine. MAO B assay: [MAO B] = 1.674 μM, substrate = 125 μM tyramine.

Supplementary Table 3 Major cellular processes that were significantly affected by corin treatment.

**Gene Ontology Process**	**Number of Genes Affected**	***p*-value**
Cell Proliferation	72	0.00149
Cellular Differentiation	29	0.00316
Cell Migration	28	4.41E-05
Cell Motility	22	1.52E-05
Cell Division	22	5.79E-06
Chromosome Segregation	11	2.77E-08
Leukocyte Migration	9	0.00992
Lymphocyte Migration	5	0.00232

The second column represents the number of genes that were upregulated by at least 2σ with corin treatment compared to MS-275 for the process identified. The *p*-values provide a measure as to how significantly each particular process was affected.

Supplementary Table 4 Tumor suppressor genes that were significantly upregulated (2-fold) by both corin and MS-275, but were upregulated at least 1.5-fold more by corin.

Gene Symbol	Gene Name	Fold Increase (corin vs DMSO)	*p*-value (corin vs DMSO)	Fold Increase (corin vs MS-275)	*p*-value (corin vs MS-275)
MT1G	metallothionein 1G	52.9086	3.11E-07	17.2245	2.23E-06
DUSP1	dual specificity phosphatase 1	10.3256	9.17E-11	4.49407	1.29E-09
TFPI2	tissue factor pathway inhibitor 2	27.2894	1.30E-09	4.08157	2.16E-07
TP63	tumor protein p63	8.87469	2.61E-08	3.56835	6.53E-07
CLU	clusterin	11.9682	1.20E-07	3.27537	9.43E-06
EPHB2	EPH receptor B2	9.81271	1.91E-08	2.74157	2.47E-06
EPHB3	EPH receptor B3	8.71412	2.86E-09	2.51884	4.63E-07
DUSP5	dual specificity phosphatase 5	7.43214	3.01E-08	2.50852	3.09E-06
TNFSF9	tumor necrosis factor superfamily member 9	7.61012	7.08E-07	2.50284	7.28E-05
CSRNP1	cysteine-serine-rich nuclear protein 1	5.02657	8.74E-09	2.24736	5.37E-07
EGR1	early growth response 1	2.49E-08	6.53784	2.22118	4.02E-06
UNC5D	unc-5 netrin receptor D	4.83005	2.47E-06	2.14599	0.000161
LINC-PINT	long intergenic non-protein coding RNA, p53 induced transcript	8.36867	1.53E-09	2.05998	9.57E-07
PCDH9	protocadherin 9	4.10077	1.70E-06	2.04288	8.86E-05
UNC5B	unc-5 netrin receptor B	4.58766	1.03E-06	2.00321	9.88E-05
CITED2	Cbp/p300-interacting transactivator, with Glu/Asp rich carboxy-terminal domain, 2	4.9016	2.45E-07	1.87579	5.61E-05
ZFP36	ZFP36 ring finger protein	4.25728	1.20E-08	1.8211	2.29E-06
CAMTA1	calmodulin binding transcription activator 1	4.09065	1.59E-06	1.74568	0.00033
CXCL14	chemokine (C-X-C motif) ligand 14	8.43421	2.89E-08	1.70256	0.000101
GLIPR1	GLI pathogenesis-related 1	7.31817	5.37E-07	1.67251	0.001201
VWA5A	von Willebrand factor A domain containing 5A	4.15315	4.00E-07	1.63342	0.000195
LIFR	leukemia inhibitory factor receptor alpha	10.2522	1.52E-08	1.63017	0.000146
BRINP1	bone morphogenetic protein/retinoic acid inducible neural-specific 1	3.69831	0.000158	1.62025	0.021384
TMEFF2	transmembrane protein with EGF-like and two follistatin-like domains 2	8.08072	1.54E-07	1.56505	0.001067
SLIT2	slit guidance ligand 2	8.35948	2.69E-11	1.52307	4.32E-07
IQGAP2	IQ motif containing GTPase activating protein 2	3.64524	7.08E-07	1.50728	0.000519

Supplementary Table 5 Tumor suppressor genes that were significantly upregulated (2-fold) by corin but not MS-275.

Gene Symbol	Gene Name	Fold Increase (corin vs DMSO)	*p*-value (corin vs DMSO)	Fold Increase (corin vs MS-275)	*p*-value (corin vs MS-275)
ATF3	activating transcription factor 3	7.47311	1.17E-05	4.4264	6.68E-05
ASS1	argininosuccinate synthase 1	7.36801	3.55E-09	3.8762	3.62E-08
GADD45B	growth arrest and DNA damage inducible beta	5.44369	1.79E-08	3.80599	7.40E-08
PAEP	progestagen-associated endometrial protein	6.79171	6.35E-06	3.79397	5.18E-05
IRF8	interferon regulatory factor 8	4.66797	1.28E-06	3.25053	6.18E-06
SIK1	salt inducible kinase 1	4.68579	3.62E-07	3.12317	2.20E-06
IL24	interleukin 24	3.29405	2.41E-05	2.91368	4.50E-05
NR4A3	nuclear receptor subfamily 4 group A member 3	3.80012	5.24E-06	2.82569	2.26E-05
TNFRSF10B	tumor necrosis factor receptor superfamily member 10b	3.5807	2.28E-07	2.7861	8.38E-07
ERRFI1	ERBB receptor feedback inhibitor 1	5.0274	9.45E-07	2.7152	1.59E-05
DNAJB1	DnaJ heat shock protein family (Hsp40) member B1	2.57638	5.60E-06	2.69671	4.25E-06
CNN1	calponin 1, basic, smooth muscle	3.92382	1.83E-06	2.68243	1.24E-05
MEG3	maternally expressed 3 (non-protein coding)	2.94187	6.73E-05	2.53168	0.000157
CDH4	cadherin 4, type 1, R-cadherin (retinal)	2.63863	3.48E-06	2.49746	4.90E-06
CDH13	cadherin 13	2.75075	1.35E-05	2.46954	2.59E-05
CEBPA	CCAAT/enhancer binding protein (C/EBP), alpha	3.23415	3.72E-05	2.43462	0.000178
BIK	BCL2-interacting killer (apoptosis-inducing)	3.13428	4.54E-05	2.41783	0.000195
GADD45A	growth arrest and DNA damage inducible alpha	4.60353	7.04E-08	2.41136	1.86E-06
FLNA	filamin A, alpha	2.68403	1.27E-05	2.28539	3.55E-05
PCDH8	protocadherin 8	2.76886	1.02E-06	2.21278	4.44E-06
TNFRSF10A	tumor necrosis factor receptor superfamily member 10a	2.16635	3.49E-05	2.2114	3.00E-05
LTF	lactotransferrin	3.9465	1.82E-07	2.20382	4.79E-06
ETS2	v-ets avian erythroblastosis virus E26 oncogene homolog 2	2.7404	4.98E-08	2.1974	2.18E-07
DCUN1D3	DCN1, defective in cullin neddylation 1, domain containing 3	3.5486	1.07E-05	2.1815	0.000172
TBRG1	transforming growth factor beta regulator 1	2.38338	1.15E-05	2.16879	2.24E-05
HSP90B1	heat shock protein 90kDa beta family member 1	2.18091	1.27E-06	2.16851	1.33E-06
KMT2C	lysine (K)-specific methyltransferase 2C	2.63828	6.89E-08	2.15515	2.77E-07
AZGP1	alpha-2-glycoprotein 1, zinc-binding	2.41922	7.73E-05	2.045	0.000252
BCL2L11	BCL2-like 11	2.92706	7.96E-06	2.0448	8.29E-05
DAPK3	death-associated protein kinase 3	2.66647	1.25E-05	2.02944	8.13E-05

Gene Symbol	Gene Name	Fold Increase (corin vs DMSO)	*p*-value (corin vs DMSO)	Fold Increase (corin vs MS-275)	*p*-value (corin vs MS-275)
EPHA2	EPH receptor A2	3.61017	2.07E-08	1.94775	1.02E-06
VEGFA	vascular endothelial growth factor A	2.36093	7.05E-06	1.92501	3.41E-05
NDRG1	N-myc downstream regulated 1	3.39264	1.82E-07	1.88599	8.73E-06
DEFB1	defensin beta 1	2.52609	0.000305	1.87175	0.002381
MAP3K8	mitogen-activated protein kinase kinase kinase 8	2.66995	1.33E-05	1.87067	0.000174
GPC3	glypican 3	2.24841	0.001197	1.8704	0.004333
AJAP1	adherens junctions associated protein 1	2.13421	0.000226	1.86229	0.000665
SRGAP3	SLIT-ROBO Rho GTPase activating protein 3	2.98824	5.41E-06	1.84463	0.000153
CDKN1A	cyclin-dependent kinase inhibitor 1A	3.51207	2.47E-07	1.7973	2.19E-05
ANGPTL4	angiopoietin like 4	3.32293	7.32E-05	1.7868	0.003545
SIRT4	sirtuin 4	2.12869	6.88E-05	1.7791	0.000313
ATMIN	ATM interactor	2.14881	1.16E-05	1.7701	6.27E-05
FBXO31	F-box protein 31	2.10762	8.51E-06	1.70075	6.04E-05
RBM38	RNA binding motif protein 38	2.07943	0.002542	1.67961	0.012579
IRF1	interferon regulatory factor 1	2.43335	1.46E-05	1.65251	0.000369
STAT3	signal transducer and activator of transcription 3 (acute-phase response factor)	2.82122	5.23E-08	1.63042	4.54E-06
KLF4	Kruppel-like factor 4 (gut)	2.69937	3.05E-07	1.61681	2.19E-05
BEX2	brain expressed X-linked 2	2.61799	0.000144	1.60783	0.005686
ARNTL	aryl hydrocarbon receptor nuclear translocator like	2.50779	7.44E-07	1.60499	3.64E-05
SAA1	serum amyloid A1	2.40743	0.00019	1.57848	0.005626
KIF1B	kinesin family member 1B	2.14684	2.14E-06	1.57825	4.31E-05
SOCS1	suppressor of cytokine signaling 1	2.14279	0.000767	1.56825	0.010086
ALPL	alkaline phosphatase, liver/bone/kidney	2.12582	2.24E-05	1.55768	0.000445
EXT1	exostosin glycosyltransferase 1	2.25619	2.03E-08	1.5281	9.87E-07
EPAS1	endothelial PAS domain protein 1	2.90537	4.21E-06	1.52324	0.000811
ZBTB7C	zinc finger and BTB domain containing 7C	2.01201	0.001663	1.52159	0.017629
HDAC3	histone deacetylase 3	2.21948	4.32E-06	1.51683	0.00018
FAT1	FAT atypical cadherin 1	2.11015	8.86E-08	1.50403	3.19E-06
PTPN13	protein tyrosine phosphatase, non-receptor type 13 (APO-1/CD95 (Fas)-associated phosphatase)	2.22025	2.33E-08	1.49155	1.42E-06
BTG2	BTG family member 2	2.20739	5.20E-06	1.48562	0.000275
SSBP2	single-stranded DNA binding protein 2	2.06066	5.26E-05	1.47339	0.001586
TP53INP1	tumor protein p53 inducible nuclear protein 1	2.57599	3.52E-06	1.4649	0.000613
PRDM2	PR domain containing 2, with ZNF domain	2.0017	9.28E-05	1.44938	0.002652

Gene Symbol	Gene Name	Fold Increase (corin vs DMSO)	*p*-value (corin vs DMSO)	Fold Increase (corin vs MS-275)	*p*-value (corin vs MS-275)
EAF2	ELL associated factor 2	2.04704	7.25E-05	1.40744	0.003747
OSGIN1	oxidative stress induced growth inhibitor 1	2.1722	1.15E-05	1.406	0.001135
GLS2	glutaminase 2 (liver, mitochondrial)	2.24202	0.000464	1.39892	0.028742
SLC9A3R1	solute carrier family 9, subfamily A (NHE3, cation proton antiporter 3), member 3 regulator 1	2.25275	2.34E-07	1.39862	4.21E-05
TSLP	thymic stromal lymphopoietin	2.08222	8.36E-05	1.39211	0.005511
CLDN23	claudin 23	2.31661	4.52E-06	1.38357	0.000964
PPP2R2C	protein phosphatase 2 regulatory subunit B, gamma	2.21687	0.000104	1.34641	0.014998
BCORL1	BCL6 corepressor-like 1	2.35152	4.28E-06	1.29806	0.00306
TES	testin LIM domain protein	2.24849	6.05E-06	1.28524	0.003789
SEMA3F	sema domain, immunoglobulin domain (Ig), short basic domain, secreted, (semaphorin) 3F	2.35778	1.69E-05	1.25368	0.017169
VSNL1	visinin like 1	2.23615	0.001825	1.22936	0.222531
ANXA1	annexin A1	2.29326	1.97E-06	1.14789	0.024977
EPHB6	EPH receptor B6	2.27636	2.78E-06	1.14038	0.035909

Supplementary Table 6 Buffer, plasma, and microsomal stability of corin.

Compound	Time (min)	Buffer	Plasma (human)	MLM - NADPH	MLM + NADPH	HLM - NADPH	HLM + NADPH
	0	100%	100%	100%	100%	100%	100%
**Corin**	30	109%	134%	124%	100%	63%	77%
	60	106%	107%	85%	87%	59%	68%

Buffer = 0.5 M sodium-potassium phosphate (pH 7.4), MLM = mouse liver microsomes; HLM = human liver microsomes

Supplementary Table 7 shRNA constructs used for knockdown experiments.

Gene	Construct ID Number	Construct Sequence
RCOR1	TRCN0000129660 (construct 1)	CCGGCAAACGACAGATCCAGAATATCTCGAGATATTCTGGATCTGTCGTTTGTTTTTTG
RCOR1	TRCN0000128570 (construct 2)	CCGGGATGGTGGAATAGAACCATATCTCGAGATATGGTTCTATTCCACCATCTTTTTTG
SIN3A	TRCN0000021774	CCGGCGTGAACATCTAGCACAGAAACTCGAGTTTCTGTGCTAGATGTTCACGTTTTT

Supplementary Table 8 Primers used for quantitative real time-PCR.

Gene	Primers
p21	5′-GGCGGGCTGCATCCA-3′<br>5′-AGTGGTGTCTCGGTGACAAAGTC-3′
CHOP	5′-CAGAACCAGCAGAGGTCACA-3′<br>5′-AGCTGTGCCACTTTCCTTTC-3′
MXD1	5′-ACCTGAAGAGGCAGCTGGAGAA-3′<br>5′-AGATAGTCCGTGCTCTCCACGT-3′
SIK1	5’-GTCTGGGCGCACCTTTAGCA-3’<br>5’-CAGCCTTTCGCCCAGTCTTT-3’
RCOR1	5′-GAGGCAAATGGAAACAATCCC-3′<br>5′-CCTGAGGAACTGTCTCAGTAG-3′
β-actin	5′-CCAGCTCACCATGGATGATG-3′<br>5′-ATGCCGGAGCCGTTGTC-3′
p53	5′-CGCCATGGCCATCTACAAG-3′<br>5′-GGGCAGCGCCTCACAA-3′
NR4A3	5′-CCGCTCCTCCTACACTCTCA-3′<br>5′-TGGACGCAGGGCATATCT-3′
TNFRSF10B	5′-AAGCCGGGGACCAAGGAGACAGACAAC-3′<br>5′-TGCCGGGGCCCTTTTTCAGAGT-3′
NBPF1	5′-GCGAGGCTGCCCGAGCTTCT-3′<br>5′-GACTTCGCGTAACTTCCCATTCA-3′
RND1	5′-AGCGCACTCAAGAAGTGGAGGAC-3′<br>5′-GCCTGCTTCTGGTGGGACAGC-3′
NEFH	5′-AAAAAGGAAGAGGTGAAGTCCC-3′<br>5′-GAGTGCCCTCTCTTGCTAACAT-3′
MBD3L2	5′-CTGTTCTGGGGAAGCTCAAAAGGAAC-3′<br>5′-GGTCTCTCACTTTCTCTGCCTCA-3′
RASD1	5′-CCTCTCCATCCTCACAGGAG-3′<br>5′-GGCAAGACTTGGTGTCGAG-3′
DUX4L1	5'-CCCAGGTACCAGCAGACC-3'<br>5'-TCCAGGAGATGTAACTCTAATCCA-3'
SYN1	5’-CAACGGAGACTACCGCAGTTTGGTC-3’<br>5’-GAAATCACCCTTTAGATGTACCAGAAGTAGAGG-3’
VAMP7	5’-GAAAGCCAAACTCAACCTCAAGTC-3’<br>5’-ATGATGCACAGGGTTTTTCTGG-3’

Supplementary Table 9 Primers used for ChIP-PCR.

Gene	Region	Primers
CHOP	P1	5'-ACCTTTTACCCTCCCGTCTC-3'<br>5'-GTCCGAAGCAATAGGGGTTT-3'
	P2	5'-GCAAGGCCATGGTAAAAGAT-3'<br>5'-GGGAATCAGTACTCGCCTCT-3'
	P3	5'-GAAGCCTCGTGACCCAAAG-3'<br>5'-AAAACCTACCAATCAGAAAGTGG-3'
	P4	5'-CCCGCCCTCTCTCCTCTC-3'<br>5'-GGGATGATGCAATGTTTGG-3'
	P5	5'-GCCTATGTGCCCATTAGCTG-3'<br>5'-ACGGCTATCAGCCTTGGTC-3'
MXD1	P1	5'-TGACTGTCCTATCTTTCCATGTTC-3'<br>5'-AAACATCGTCCCTGGCAAC-3'
	P2	5'-AATCTGTTACCACCTCTCTTTTTATTT-3'<br>5'-GTGGAGTCAAAGGAGGCAAA-3'
	P3	5'-TCATGCCTGATATTAAAGGTAGTCC-3'<br>5'-CCTCACTAGCAGAGAACACACAA-3'
	P4	5'-CTCCCAAAGTGCTGGGATTA-3'<br>5'-TTCTGAAGACACTTCCAAAATAGAA-3'
	P5	5'-CTCAGACTGGAGTGCAATGG-3'<br>5'-AACATGGTGAGAACCCGTCT-3'
SYN1	P1	5'-AAGCACCCAACCCCCATT-3'<br>5'-GGTGCTGAAGCTGGCAGT-3'
	P2	5'-TCTGAGTGTGCTTCCAGTGC-3'<br>5'-GTCGGTCGTCAGGTAGGC-3'
	P3	5'-GTACACCACCCAAGTGTCCA-3'<br>5'-GAGAAATAACCCCCTCACAGA-3'
	P4	5'-ACCTGTGTGTGTGGGGGTAT-3'<br>5'-CCTGGAGGCACACAAGAAAT-3'
	P5	5'-GCCTGCCTCTGTGGAAAGT-3'<br>5'-ACATTCTCTGCAGCCCTAGC-3'
VAMP7	P1	5'-TATGCCGCCTACTCATTGCT-3'<br>5'-CCGTACTGCATTCTGGGAGT-3'
	P2	5'-TGCAAAAAGAATAGGAGAAAGGA-3'<br>5'-CCCCGTCTCTCCTGCTTAGT-3'
	P3	5'-TGAGAGAAATTTTGACAGCCTTT-3'<br>5'-ATCTAAGCCAGCCCTCTTCC-3'
	P4	5'-TCCTGCAGAGTTGAAATACGG-3'<br>5'-CTTAACCCCATACCCCAAGC-3'
	P5	5'-GGTCAGGAGTTCGAGACCAG-3'<br>5'-TCACTGCCAGCTCCGTCT-3'

SUPPLEMENTARY METHODS

Molecular modeling. We used the Rosetta Molecular Modeling Suite[1] to determine plausible conformations that allowed the FAD-corin ligand to be simultaneously bound by LSD1 (PDB ID: 2XAG) and HDAC2 (PDB ID: 4LY1). Ligand-bound crystal structures exist for the proteins. However, we manually generated the corin and flavin cofactor structures. We parameterized the molecules by performing density functional theory calculations using the Gaussian 09 package[2]. These calculations estimated bond lengths, bond angles, torsion angles, and partial atomic charges on these molecules in their ground state. We used the B3LYP hybrid functional[3] and the 6-31+g(d) basis set with diffuse function for both flavin cofactor and corin. Water was represented using the polarizable continuum model[4] for these calculations. Partial atomic charges were derived from population analysis using the CM5 model[5]. Using PyRosetta[6], we assembled the covalently linked FAD-corin, LSD1, and HDAC ternary complex, with both proteins binding the ligand as observed in their respective crystal structures. Next, possible conformations of the free corin ligand were generated by the Frog2 server[7]. In the assembled structure, we sampled the preferred conformations of corin, while constraining it to coordinate the Zn^{2+} cofactor in HDAC2. Thus, any conformational change in corin resulted in a change to the relative orientation of LSD1 and HDAC2. The energy of each hypothetical conformation was evaluated using the Rosetta centroid energy function[8]. The twenty structures with the fewest backbone clashes between LSD1 and HDAC2 were chosen for further sampling and refinement in the full-atom energy function[9]. These structures were subject to a Monte Carlo search of all mobile dihedral angles of corin through ±10° of their starting values. From this refined set, we selected five structures with low energies and without secondary structure clashes.

To alleviate residual steric clashes, we re-modeled the loops at the interface. These loops are solvent exposed in the respective crystal structures and are flexible according to normal mode analysis[10]. Loop re-modeling was done using a kinematic loop closure algorithm[11,12]. Side-chain re-packing[13] and gradient-based energy minimization[14] was carried out multiple times during loop modeling. Throughout the simulation, the alpha-carbon atoms of residues in elements of secondary

structure were constrained with harmonic potentials to their starting positions. In addition, Zn^{2+}-binding residues were constrained and the FAD-corin ligand was not permitted to move. The results suggested that energetically stable conformations allowing for corin dual occupancy without clashes and with excellent geomtery are feasible.

Histone extraction. For cSCC Western blots, histones were extracted prior to gel loading[15]. Cells were collected in cold PBS (pH 7.4) and pelleted at 300 x *g* for 5 min. Cells were resuspended in hypotonic lysis buffer (10 mM Tris-HCl pH 8.0, 1 mM KCl, 1.5 mM $MgCl_2$, 1 mM DTT, and 1X Roche EDTA-free Complete Protease Inhibitor cocktail) and rotated end over end at 4 °C for 30 min. Nuclei were pelleted by spinning at 10,000 x *g* for 10 min at 4 °C. The supernatant was discarded and the nuclei were resuspended in 0.4 N H_2SO_4 (400 µl) and rotated end over end at 4 °C for 30 min. Histones were pelleted at 16,000 x *g* for 10 min at 4 °C, the supernatant was discarded, and the histones were washed with cold acetone (2 x 150 µl). After decanting acetone, histones were air-dried at room temperature, resuspended in water (150 µl), and protein concentration was determined using the micro BCA assay (ThermoFisher Scientific).

Overview of synthetic routes. Hybrid compounds **1-6** were designed such that they contained standard HDAC zinc binding groups, a benzamide (as in MS-275) or a hydroxamic acid (as in SAHA, LBH589), tethered to an amine oxidase warhead, either phenelzine or tranylcypromine (Fig. 1). Synthesis of compound **1** was initiated via the Sonogoshira coupling of 3-butyn-1-ol to 4-bromobenzoic acid methyl ester to produce an alkyne intermediate which was subsequently reduced via palladium catalyzed hydrogenation (Supplementary Fig. 34)[16]. The resulting alcohol **10** was oxidized to a carboxylic acid in a two-step process using pyridinium chlorochromate to generate the aldehyde followed by treatment with sodium chlorite[16,17]. The intermediate acid **11** was coupled to 2-(4-aminophenyl)ethanol under standard conditions to yield alcohol **12**, which was further converted to the alkyl bromide via the Appel reaction[18]. The bromide was subsequently displaced with excess di-*tert*-butylhydrazodiformate to provide intermediate ester **13**[19]. Saponification with lithium hydroxide followed by coupling to the protected *o*-phenylenediamine **15** under standard conditions resulted in the penultimate product which was deprotected with trifluoroacetic acid (TFA) to yield the desired dual inhibitor **1**.

To generate intermediate **15**, 2-nitroaniline was protected using di-*tert*-butyl dicarbonate[20] resulting in the protected nitroaniline **14** which was subsequently reduced to the desired diamine via palladium catalyzed hydrogenation (Supplementary Fig. 35).

Compound **2** was prepared using a convergent approach (Supplementary Fig. 36-38). Cyclopropanation of 4-nitrostyrene with ethyl diazoacetate using dimeric rhodium acetate as a catalyst yielded the intermediate ester as a mixture of geometric isomers (Supplementary Fig. 36). This isomeric mixture was saponified with lithium hydroxide and subsequently converted to the acid chloride by heating to reflux in thionyl chloride. Treatment of the acid chloride with sodium azide resulted in acyl azide **16**, which allowed for the resolution of the geometric isomers by normal phase column chromatography. Curtius rearrangement was carried out on the less polar *trans* isomer, still present as a racemic mixture of enantiomers, to yield the intermediate isocyanate which was immediately treated

with *tert*-butanol to provide the boc protected cyclopropylamine **17**[21]. The nitro group was reduced to the corresponding amine with palladium on carbon and sodium hypophosphite to yield the first synthetic building block, compound **18**, for the preparation of dual inhibitor **2**.

The second building block was prepared in three steps from 4-bromobenzoic acid ethyl ester and 3-butyn-1-ol following the same procedure that was employed to prepare carboxylic acid intermediate **11** (Supplementary Fig. 37). Sonogoshira coupling provided alkyne intermediate **19**. Then, the triple bond was reduced under hydrogen atmosphere in the presence of a palladium catalyst to generate intermediate alcohol **20** which was subsequently oxidized in two steps to the desired acid **21**.

To complete the synthesis, intermediates **18** and **21** were coupled with HATU to yield ester **22** which was saponified with lithium hydroxide to provide the desired acid **23** (Supplementary Fig. 38). The intermediate acid was coupled to the previously synthesized, protected *o*-phenylenediamine **15** to yield penultimate compound **24**. Subsequent deprotection with TFA produced the desired final product **2** (corin).

Dual inhibitor **3** was prepared from commercially available 3-(4-bromobenzoyl)propionic acid in nine steps (Supplementary Fig. 39). First, the keto acid starting material was reduced under Wolff-Kishner conditions[22] to yield 4-(4-bromophenyl)butyric acid **25** which was subsequently coupled to 2-(4-aminophenyl)ethanol, as described for compound **1**, to yield the intermediate alcohol **26**. Protection of the alcohol as a silyl ether provided compound **27**[23] which was subjected to Heck coupling with methyl acrylate[24] to yield unsaturated ester **28**. Deprotection of the alcohol with TBAF[25] yielded intermediate **29** which was subjected to the Appel reaction[18] to generate alkyl bromide **30**. Nucleophilic substitution with di-*tert*-butylhydrazodiformate[19] produced the penultimate compound **31** which was converted to the hydroxamic acid with hydroxylamine[26] and subsequently deprotected with TFA to yield desired final product **3**.

Dual inhibitors **4** and **5** were prepared in seven steps starting with 4-(4-nitrophenyl)butyric acid (Supplementary Fig. 40). 2-(4-Aminophenyl)ethanol was coupled to 4-(4-nitrophenyl)butyric acid using HATU to generate intermediate **32** which was subsequently hydrogenated in the presence of palladium to yield common intermediate amine **33**. Standard peptide coupling conditions were again utilized with **33** and either suberic or adipic acid monomethyl ester to generate compounds **34** and **35**, respectively. Separately, intermediates **34** and **35** were subjected to the Appel reaction[18] resulting in alkyl bromides **36** and **37**. Substitution of the halide by di-*tert*-butylhydrazodiformate[19] yielded penultimate intermediates **38** and **39** which were converted to their respective hydroxamic acids using hydroxylamine[26] and ultimately deprotected to provide dual inhibitors **4** and **5**.

Lastly, dual inhibitor **6** was prepared in nine total steps, also using a convergent approach (Supplementary Fig. 41). Ethyl 4-aminobenzoate was acylated using suberic anhydride to generate free acid **40**. The acid was converted to a mixed carbonic anhydride using ethyl chloroformate and subsequently converted to the tetrahydropyranyl ether protected hydroxamate **41**. The ester was saponified with lithium hydroxide to yield the acid **42** and then coupled to compound **18** using standard amide bond forming conditions. The penultimate product **43** was then deprotected with hydrochloric acid (HCl) to yield the desired dual inhibitor **6** as a hydrochloride salt.

Compound **7** was generated by coupling intermediate amine **18** with commercially available 4-phenylbutyric acid and subsequently removing the protecting groups with TFA (Supplementary Fig. 42). Compound **8** was prepared as previously described[21]. Control compound **9** was synthesized following a route similar to that used for the preparation of corin (Supplementary Fig. 43).

General. ^1H and ^{13}C NMR experiments were run using either a Bruker 500 MHz (^1H, 500 MHz; ^{13}C, 125 MHz) spectrometer or a Bruker AC 400 spectrometer (^1H, 400 MHz; ^{13}C, 100 MHz). Chemical shifts (δ) are presented in parts per million (ppm) relative to the solvent residual peaks (^1H NMR: $CHCl_3$, δ = 7.27 ppm; DMSO, δ = 2.50 ppm; CH_3OH, δ = 3.31; ^{13}C NMR: $CHCl_3$, δ = 77.00 ppm; DMSO, δ = 39.51 ppm; CH_3OH, δ = 49.15 ppm), and J-coupling constants (*J*) are expressed in hertz (Hz). The following designations were used to indicate multiplicity: s (singlet), d (doublet), t (triplet), q (quartet), quin (quintet), m (multiplet), br (broad), dd (doublet of doublets), dt (doublet of triplets), td (triplet of doublets), ddd (doublet of doublet of doublets). Processing of NMR spectra was carried out using ACD/NMR Processor Academic Addition, version 12.01 (Advanced Chemistry Development, Inc., Toronto, On, Canada, www.acdlabs.com, 2013). ESI-HRMS data was obtained on either a Shimadzu IT-TOF spectrometer at the Research Resources Center's Mass Spectrometry Facility at the University of Illinois at Chicago, a Waters Q-TOF Ultima ESI at the School of Chemical Sciences Mass Spectrometry Laboratory at the University of Illinois at Urbana-Champaign, or a Thermo Scientific Q Exactive at the Johns Hopkins University School of Medicine. EI-HRMS data was obtained on a Waters 70-VSE spectrometer, also at the School of Chemical Sciences Mass Spectrometry Laboratory at the University of Illinois at Urbana-Champaign. EI-MS spectra were recorded with a Fisons Trio 1000 spectrometer with only molecular ions (M^+) and base peaks reported. Melting points were determined on a Buchi 530 melting point apparatus and are uncorrected. Chemicals and reagents were purchased from commercial vendors and used as received. Solvents were also purchased from commercial vendors and, when necessary, purified and dried using standard techniques. Reaction progress was monitored by thin layer chromatography (TLC) using either pre-coated, glass silica gel plates (Sigma-Aldrich F254, 60 Å pore size, 250 µM thickness) or aluminum-backed silica gel plates (Merck DC, Alufolien Kieselgel 60 F254) with spots visualized by UV light. Automated column chromatography was carried out on a Grace Reveleris® X1 Flash Chromatography System (Columbia, MD). Preparatory HPLC was carried out using a Varian ProStar 210 with the following specifications: Column: Varian Dynamax (250 x 21.4 mm, 5 µm particle size) Microsorb 100-5 C18 fitted with a guard column. Flow

rate: 10 ml min^{-1}, λ monitoring at 254 nm. Gradient: 5% MeCN/H_2O, 1 min; 5-60% MeCN/H_2O, 50 min; 60-100% MeCN/H_2O, 5 min; 100% MeCN, 5 min; 100-5% MeCN/H_2O, 5 min; 5% MeCN/H_2O, 5 min. Analytical HPLC was carried out using the same instrument but with the following specifications: Column: Agilent Eclipse XD8-C18 (4.6 x 250 nm, 5 µm particle size). Flow rate: 1 ml min^{-1}, λ monitoring at 254 nm. Gradient: 10% MeCN/H_2O, 1 min; 10-100% MeCN/H_2O, 19 min; 100% MeCN, 3 min; 100-10% MeCN/H_2O, 2 min; 10% MeCN/H_2O, 5 min. All HPLC solvents were spiked with 0.05% TFA. All compounds tested were determined to be ≥95% pure as determined by ^{1}H NMR, analytical HPLC, and/or elemental analysis. For elemental analysis, analytical results were within ± 0.40% of the theoretical values.

Methyl 4-(4-hydroxybutyl)benzoate (10):

4-Bromobenzoic acid methyl ester (2.150 g, 10.00 mmol), palladium(II) chloride (0.089 g, 0.50 mmol), triphenylphosphine (0.262 g, 1.00 mmol), and copper(I) iodide (0.190 g, 1.00 mmol) were placed in a round-bottomed flask under argon and suspended in diethylamine (30 ml) at room temperature. 3-Butyn-1-ol (0.757 ml, 10.00 mmol) was added and the reaction was stirred for 24 h during which time the reaction mixture turned from light yellow to black. After completion as evidenced by TLC, diethylamine was removed under reduced pressure. Water (50 ml) was added to the resulting residue and the organic products were extracted with methylene chloride (3 x 30 ml). The combined organic extracts were washed with brine (20 ml), dried with anhydrous sodium sulfate, and poured through a 1.5 inch pad of celite to remove residual catalyst. The celite plug was washed with methylene chloride (3 x 30 ml) and the combined filtrate and washes were concentrated in vacuo to yield a crude orange solid. Purification by column chromatography (SiO_2, 25% EtOAc/hexanes) afforded methyl 4-(4-hydroxybut-1-yn-1-yl)benzoate as a beige, crystalline solid (1.745 g, 85%). ^{1}H NMR (500 MHz, $CDCl_3$): δ 7.96 (d, *J* = 8.6 Hz, 2H), 7.46 (d, *J* = 8.5 Hz, 2H), 3.91 (s, 3H), 3.83 (t, *J* = 6.3 Hz, 2H), 2.71 (t, *J* = 6.3 Hz, 2H), 2.08 (br, 1H). ^{13}C NMR (125 MHz, $CDCl_3$): δ 166.56, 131.54, 129.38, 129.17, 128.11, 89.85,

81.70, 60.97, 52.16, 23.83. ESI-HRMS: calcd. for $C_{12}H_{12}O_3$: $[M+H]^+$ = *m/z* 205.0859, found: $[M+H]^+$ = *m/z* 205.0864.

Methyl 4-(4-hydroxybut-1-yn-1-yl)benzoate (3.320 g, 16.26 mmol) and 10% palladium on carbon (0.664 g, 20% wt. equivalent) were suspended in 95% ethanol (125 ml) and placed under a H_2 atmosphere (60 psi) at room temperature. The suspension was agitated for 12 h after which it was complete as evidenced by TLC. The reaction mixture was filtered through a 1.5 inch celite plug and the filter cake was washed with methanol (3 x 30 ml). The combined filtrate and washes were concentrated in vacuo to yield the desired product as a viscous, yellow oil (3.159 g, 93%). ^{1}H NMR (500 MHz, CD_3OD): δ 7.90 (d, *J* = 8.3 Hz, 2H), 7.28 (d, *J* = 8.5 Hz, 2H), 3.86 (s, 3H), 3.56 (t, *J* = 6.5 Hz, 2H), 2.68 (t, *J* = 7.7 Hz, 2H), 1.69 (m, 2H), 1.55 (m, 2H). ^{13}C NMR (125 MHz, CD_3OD): δ 168.70, 149.86, 130.73, 129.75, 128.97, 62.76, 52.59, 36.72, 33.28, 28.66. ESI-HRMS: calcd. for $C_{12}H_{16}O_3$: $[M+H]^+$ = *m/z* 209.1172, found: $[M+H]^+$ = *m/z* 209.1181.

4-[4-(Methoxycarbonyl)phenyl]butanoic acid (11):

A two-necked round-bottomed flask fitted with a condenser was charged with pyridinium chlorochromate (4.905 g, 22.75 mmol) and sodium acetate (1.244 g, 15.17 mmol) and placed under argon. Anhydrous methylene chloride (135 ml) was added and stirring was initiated. Methyl 4-(4-hydroxybutyl)benzoate **10** (3.159 g, 15.17 mmol) was dissolved in anhydrous methylene chloride (65 ml) and added to the reaction. The reaction turned from dark orange to black over approximately 15 min and stirring was continued for 12 h. After completion, it was poured through a 1.5 inch silica gel pad which was subsequently washed with methylene chloride (3 x 30 ml). The combined filtrate and washes were concentrated in vacuo and the resulting residue was purified by column chromatography (SiO_2, 10-25% EtOAc/hexanes) to yield methyl 4-(4-oxobutyl)benzoate as a clear, viscous oil (2.126 g, 68%). Of note, some over oxidation to the acid was observed. ^{1}H NMR (500 MHz, $CDCl_3$): δ 9.77 (t, *J* = 1.4 Hz, 1H), 7.97 (d, *J* = 8.3 Hz, 2H), 7.25 (d, *J* = 8.3 Hz, 2H), 3.91 (s, 3H), 2.72 (t, *J* = 7.6 Hz, 2H), 2.47 (td, *J* = 7.3 Hz, 1.4 Hz, 2H), 1.98 (quin, *J* = 7.4 Hz, 2H). ^{13}C NMR (125 MHz, $CDCl_3$): δ 201.83,

167.00, 146.68, 129.78, 128.44, 128.12, 51.98, 42.99, 34.95, 23.21. ESI-HRMS: calcd. for $C_{12}H_{14}O_3$: $[M+H]^+$ = *m/z* 207.1016, found: $[M+H]^+$ = *m/z* 207.1014.

Methyl 4-(4-oxobutyl)benzoate (2.126 g, 10.31 mmol) was dissolved in acetonitrile (10 ml) and cooled to 0 °C in and ice bath. Sodium dihydrogen phosphate monohydrate (0.356 g, 2.58 mmol) was dissolved in water (5 ml) and added to the reaction after which a 30% hydrogen peroxide solution (1.228 ml, 10.83 mmol) was slowly added at 0 °C. Then, sodium chlorite (1.305 g, 14.43 mmol) was dissolved in water (15 ml) and added dropwise via an addition funnel to the reaction over 1 h with the temperature being maintained at 0 °C. Oxygen evolved from the reaction upon addition of the oxidant and the reaction turned from pale yellow to bright yellow. The yellow color faded as the reaction proceeded and stirring was continued for 1 h after addition of the sodium chlorite. After completion, sodium sulfite (100 mg) was added to degrade hypochlorous acid and residual hydrogen peroxide. The pH was adjusted to 2 with 1 N HCl after which the organic products were extracted with ethyl acetate (3 x 20 ml), washed with brine (20 ml), dried with anhydrous sodium sulfate, filtered, and concentrated in vacuo. Purification by column chromatography (SiO_2, 25-50% EtOAc/hexanes) yielded the desired product as a white, crystalline solid (2.103 g, 92%). ^1H NMR (500 MHz, $CDCl_3$): δ 7.97 (d, *J* = 8.3 Hz, 2H), 7.26 (d, *J* = 8.3 Hz, 2H), 3.91 (s, 3H), 2.73 (t, *J* = 7.4 Hz, 2H), 2.39 (t, *J* = 7.4 Hz, 2H), 1.99 (quin, *J* = 7.5 Hz, 2H). ^{13}C NMR (125 MHz, $CDCl_3$): δ 179.45, 167.08, 146.66, 129.76, 128.46, 128.04, 51.99, 34.93, 33.17, 25.78. ESI-HRMS: calcd. for $C_{12}H_{14}O_4$: $[M-H]^-$ = *m/z* 221.0819, found: $[M-H]^-$ = *m/z* 221.0823.

Methyl 4-(4-{[4-(2-hydroxyethyl)phenyl]amino}-4-oxobutyl)benzoate (12):

4-[4-(Methoxycarbonyl)phenyl]butanoic acid **11** (2.030 g, 9.13 mmol), 2-(4-aminophenyl)ethanol (1.253 g, 9.13 mmol), and HATU (4.166 g, 10.96 mmol) were placed in a round-bottomed flask under argon and dissolved in anhydrous methylene chloride (30 ml). The reaction was cooled to 0 °C in an ice bath and then triethylamine (2.800 ml, 20.09 mmol) was added causing the reaction to become

homogenous. The reaction was allowed to warm to room temperature and stirring was continued for 16 h. After completion, the reaction was poured into 1 N HCl (15 ml) and the organic products were extracted with methylene chloride (3 x 30 ml). The combined organic extracts were washed with brine, dried with anhydrous sodium sulfate, filtered, and concentrated in vacuo. Purification by recrystallization from ethyl acetate facilitated by the dropwise addition of hexanes provided the desired product as a white solid (2.936 g, 94%). ^{1}H NMR (500 MHz, DMSO-d_6): δ 9.78 (s, 1H), 7.89 (d, *J* = 8.2 Hz, 2H), 7.46 (d, *J* = 8.5 Hz, 2H), 7.37 (d, *J* = 8.2 Hz, 2H), 7.11 (d, *J* = 8.3 Hz, 2H), 4.60 (t, *J* = 5.2 Hz, 1H), 3.83 (s, 3H), 3.55 (td, *J* = 7.1 Hz, 5.3 Hz, 2H), 2.69 (t, *J* = 7.6 Hz, 2H), 2.65 (t, *J* = 7.2 Hz, 2H), 2.30 (t, *J* = 7.5 Hz, 2H), 1.90 (quin, *J* = 7.5 Hz, 2H). ^{13}C NMR (125 MHz, DMSO-d_6): δ 170.55, 166.21, 147.63, 137.18, 134.06, 129.27, 128.94, 128.76, 127.32, 119.04, 62.29, 51.99, 38.47, 35.59, 34.53, 26.35. ESI-HRMS: calcd. for $C_{20}H_{23}NO_4$: $[M+H]^+$ = *m/z* 342.1700, found: $[M+H]^+$ = *m/z* 342.1708.

Di-*tert*-butyl 1-{2-[4-({4-[4-(methoxycarbonyl)phenyl]butanoyl}amino)phenyl]ethyl}hydrazine-1,2-dicarboxylate (13):

Methyl 4-(4-{[4-(2-hydroxyethyl)phenyl]amino}-4-oxobutyl)benzoate **12** (1.932 g, 5.66 mmol) and triphenylphosphine (2.227 g, 8.49 mmol) were placed in a round-bottomed flask under argon and dissolved in anhydrous methylene chloride (7 ml). Then, tetrabromomethane (2.816 g, 8.49 mmol) was dissolved in anhydrous methylene chloride (3 ml) and added dropwise to the reaction at room temperature after which the reaction turned from an opaque mixture to a homogenous, yellow solution. The reaction was stirred for 30 min and after completion, it was poured into water (30 ml) and the organic products were extracted with methylene chloride (3 x 15 ml). The combined organic extracts were washed with brine (15 ml), dried with anhydrous sodium sulfate, filtered, and concentrated in vacuo. Purification by column chromatography (SiO_2, 25-50% EtOAc/hexanes) afforded methyl 4-(4-{[4-(2-bromoethyl)phenyl]amino}-4-oxobutyl)benzoate as a white solid (1.473 g, 64%). ^{1}H NMR (500 MHz, $CDCl_3$): δ 7.95 (d, *J* = 8.3 Hz, 2H), 7.46 (d, *J* = 8.5 Hz, 2H), 7.37 (br, 1H), 7.25 (d, *J* = 8.2 Hz, 2H), 7.15 (d, *J* = 8.3 Hz, 2H), 3.91 (s, 3H), 3.53 (t, *J* = 7.5 Hz, 2H), 3.12 (t, *J* = 7.5 Hz, 2H), 2.75 (t, *J* =

7.5 Hz, 2H), 2.34 (t, *J* = 7.3 Hz, 2H), 2.07 (quin, *J* = 7.5 Hz, 2H). ^{13}C NMR (125 MHz, $CDCl_3$): δ 170.64, 167.07, 146.88, 136.62, 134.77, 129.74, 129.17, 128.49, 128.00, 119.99, 52.00, 38.69, 36.48, 35.01, 32.98, 26.43. ESI-HRMS: calcd. for $C_{20}H_{22}BrNO_3$: $[M-H]^-$ = *m/z* 402.0710, found: $[M-H]^-$ = *m/z* 402.0722.

Di-*tert*-butylhydrazodiformate (2.539 g, 10.93 mmol) was placed in a round-bottomed flask under argon, dissolved in anhydrous *N*,*N*-dimethylformamide (5 ml), and cooled to -40 °C in an acetonitrile/CO_2 bath. A 60% dispersion of sodium hydride in mineral oil (0.146 g, 3.64 mmol) was suspended in anhydrous *N*,*N*-dimethylformamide (10 ml) and added dropwise to the reaction. The reaction was stirred at -40 °C for 10 min and then methyl 4-(4-{[4-(2-bromoethyl)phenyl]amino}-4-oxobutyl)benzoate (1.473 g, 3.64 mmol) was dissolved in *N*,*N*-dimethylformamide (5 ml) and added dropwise to the reaction. Stirring was continued at -40 °C for 3 h after which the reaction was allowed to warm to room temperature and then poured into water (50 ml). The organic products were extracted with ethyl acetate (3 x 20 ml) and the combined organic extracts were washed with brine (20 ml), dried with anhydrous sodium sulfate, filtered, and concentrated in vacuo. Purification by column chromatography (30% EtOAc/hexanes) yielded the desired product as a clear, viscous oil (1.658 g, 82%). ^{1}H NMR (500 MHz, $CDCl_3$): δ 8.05 (br, 1H), 7.90 (m, 2H), 7.42 (br, 2H), 7.21 (br, 2H), 7.08 (br, 2H), 6.49 (br, 1H), 3.86 (br, 3H), 3.61 (br, 2H), 2.80 (t, *J* = 6.8 Hz, 2H), 2.69 (br, 2H), 2.32 (t, *J* = 7.3 Hz, 2H), 2.01 (br, 2H), 1.43 (br, 18H). ^{13}C NMR (125 MHz, $CDCl_3$): δ 170.88, 167.00, 155.71, 155.04, 147.05, 136.43, 134.51, 129.58, 128.97, 128.39, 127.75, 119.90, 81.21, 80.95, 51.86, 51.53, 36.27, 34.99, 33.35, 28.06, 28.03, 26.44. ESI-HRMS: calcd. for $C_{30}H_{41}N_3O_7$: $[M-H]^-$ = *m/z* 554.2872, found: $[M-H]^-$ = *m/z* 554.2894.

N-(2-Aminophenyl)-4-(4-{[4-(2-hydrazinylethyl)phenyl]amino}-4-oxobutyl)benzamide (1, TFA salt):

Di-*tert*-butyl 1-{2-[4-({4-[4-(methoxycarbonyl)phenyl]butanoyl}amino)phenyl]ethyl}hydrazine-1,2-dicarboxylate **13** (1.00 g, 1.80 mmol) was placed in a round-bottomed flask and dissolved in a 3:1:1

mixture of tetrahydrofuran/methanol/water (20 ml) at room temperature. Lithium hydroxide (0.086 g, 3.60 mmol) was added as one portion and stirring was continued for 16 h. After completion, the reaction was poured into 1 N HCl (15 ml) and the organic products were extracted with ethyl acetate (3 x 20 ml). The combined organic extracts were washed with brine (15 ml), dried with anhydrous sodium sulfate, filtered, and concentrated in vacuo. 4-{4-[(4-{2-[1,2-Bis(*tert*-butoxycarbonyl)hydrazinyl]ethyl}phenyl)amino]-4-oxobutyl}benzoic acid was a viscous, yellow oil that solidified under reduced pressure and was used in the next step without further purification (0.918 g, 94%). ^{1}H NMR (500 MHz, $CDCl_3$): δ 8.01 (d, *J* = 7.9 Hz, 2H), 7.41 (d, *J* = 8.2 Hz, 2H), 7.32 (br, 1H), 7.29 (d, *J* = 8.0 Hz, 2H), 7.14 (d, *J* = 6.9 Hz, 2H), 3.66 (br, 2H), 2.85 (t, *J* = 6.4 Hz, 2H), 2.78 (t, *J* = 7.3 Hz, 2H), 2.34 (t, *J* = 7.2 Hz, 2H), 2.09 (m, 2H), 1.47 (br, 9H), 1.44 (br, 9H). ESI-HRMS: calcd. for $C_{29}H_{39}N_3O_7$: $[M-H]^-$ = *m/z* 540.2715, found: $[M-H]^-$ = *m/z* 540.2733.

4-{4-[(4-{2-[1,2-Bis(*tert*-butoxycarbonyl)hydrazinyl]ethyl}phenyl)amino]-4-oxobutyl}benzoic acid (0.918 g, 1.70 mmol), *tert*-butyl (2-aminophenyl)carbamate **15** (0.354 g, 1.70 mmol), and HATU (0.776 g, 2.04 mmol) were placed in a round-bottomed flask and dissolved in anhydrous methylene chloride (20 ml) under argon. The reaction was cooled to 0 °C with an ice bath and then triethylamine (0.521 ml, 3.74 mmol) was added. The reaction was allowed to warm to room temperature and stirring was continued for an additional 6 h. After the reaction was complete as evidenced by TLC, the reaction was poured into 1 N HCl (15 ml) and the organic products were extracted with methylene chloride (3 x 30 ml). The combined organic extracts were washed with brine, dried with anhydrous sodium sulfate, filtered, and concentrated in vacuo. Purification by column chromatography (SiO_2, 15-75% EtOAc/hexanes) yielded di-*tert*-butyl 1-{2-[4-({4-[4-({2-[(*tert*-butoxycarbonyl)amino]phenyl}carbamoyl)phenyl]butanoyl}amino)phenyl]ethyl}hydrazine-1,2-dicarboxylate as a slightly yellow, crystalline solid (684 mg, 55%). ^{1}H NMR (500 MHz, $CDCl_3$): δ 9.18 (br, 1H), 7.86 (d, *J* = 8.0 Hz, 2H), 7.75 (d, *J* = 7.5 Hz, 1H), 7.57 (br, 1H), 7.42 (d, *J* = 6.4 Hz, 2H), 7.30 (dd, *J* = 7.8 Hz, 1.3 Hz, 1H), 7.24 (d, *J* = 8.2 Hz, 2H), 7.18 (m, 2H), 7.12 (d, *J* = 7.7 Hz, 2H), 7.03 (br,

1H), 6.33 (br, 1H), 3.65 (br, 2H), 2.84 (t, *J* = 7.2 Hz, 2H), 2.74 (t, *J* = 7.5 Hz, 2H), 2.30 (t, *J* = 7.4 Hz, 2H), 2.05 (quin, *J* = 7.4 Hz, 2H), 1.51 (s, 9H), 1.48 (s, 9H), 1.43 (br, 9H). ESI-HRMS: calcd. for $C_{40}H_{53}N_5O_8$: $[M-H]^-$ = *m/z* 730.3821, found: $[M-H]^-$ = *m/z* 730.3826.

Di-*tert*-butyl 1-{2-[4-({4-[4-({2-[(*tert*-butoxycarbonyl)amino]phenyl}carbamoyl)phenyl]butanoyl}amino)phenyl]ethyl}hydrazine-1,2-dicarboxylate (0.250 g, 0.34 mmol) was dissolved in methylene chloride (9 ml) and to it was added TFA (1 ml). The reaction was stirred at room temperature for 6 h after which it was complete as evidenced by TLC. Then, the reaction was concentrated in vacuo and the residue obtained was taken up in *N*,*N*-dimethylformamide and purified by preparatory HPLC. The desired product, a ditrifluoroacetic acid salt, was isolated as a white solid (0.141 g, 63%). ^{1}H NMR (500 MHz, CD_3OD): δ 7.97 (d, *J* = 8.2 Hz, 2H), 7.52 (d, *J* = 8.5 Hz, 2H), 7.35 (m, 6H), 7.22 (d, *J* = 8.6 Hz, 2H), 3.24 (t, *J* = 7.8 Hz, 2H), 2.91 (t, *J* = 7.8 Hz, 2H), 2.80 (t, *J* = 7.7 Hz, 2H), 2.43 (t, *J* = 7.4 Hz, 2H), 2.05 (quin, 7.6 Hz, 2H). ^{13}C NMR (125 MHz, CD_3OD): δ 174.24, 169.24, 148.32, 138.94, 134.15, 132.52, 131.08, 130.22, 130.00, 129.44, 128.83, 127.77, 127.67, 123.55, 121.89, 53.64, 37.34, 36.33, 32.21, 28.41. ESI-HRMS: calcd. for $C_{25}H_{29}N_5O_2$: $[M-H]^-$ = *m/z* 430.2248, found: $[M-H]^-$ = *m/z* 430.2268.

***tert*-Butyl (2-nitrophenyl)carbamate (14):**

2-Nitroaniline (1.519 g, 11.00 mmol) was placed in a round-bottomed flask under argon and dissolved in anhydrous tetrahydrofuran (10 ml). Then, a 1 M solution of sodium bis(trimethylsilyl)amide in tetrahydrofuran (22.000 ml, 22 mmol) was added rapidly to the reaction at room temperature and stirring was continued for 15 min. The reaction was deep red in color and a precipitate formed upon addition of base but dissolved with continued stirring. Di-*tert*-butyl dicarbonate (2.183 g, 10.00 mmol) was dissolved in anhydrous tetrahydrofuran (10 ml) and added rapidly to the reaction at room temperature. Stirring was continued for 2 h after which the solvent was removed in vacuo and the residue obtained was cautiously partitioned between ethyl acetate (50 ml) and 0.1 N HCl (50 ml). The organic layer was isolated and the aqueous layer was further extracted with ethyl acetate (2 x 25 ml).

The combined organic extracts were washed with brine (20 ml), dried with anhydrous sodium sulfate, filtered, and concentrated in vacuo. Purification by column chromatography (SiO_2, 15-25% EtOAc/hexanes) provided the desired product as a light yellow solid (2.004 g, 84%). ^{1}H NMR (500 MHz, $CDCl_3$): δ 9.66 (br, 1H), 8.55 (dd, *J* = 8.6 Hz, 1.2 Hz, 1H), 8.18 (dd, *J* = 8.5 Hz, 1.6 Hz, 1H), 7.60 (ddd, *J* = 8.5 Hz, 7.3 Hz, 1.3 Hz, 1H), 7.08 (ddd, *J* = 8.5 Hz, 7.2 Hz, 1.3 Hz, 1H), 1.55 (s, 9H). ^{13}C NMR (125 MHz, $CDCl_3$): δ 152.14, 135.92, 135.89, 135.67, 125.76, 121.77, 120.62, 81.76, 28.15.

***tert*-Butyl (2-aminophenyl)carbamate (15):**

tert-Butyl (2-nitrophenyl)carbamate **14** (2.000 g, 8.39 mmol) and 10% palladium on carbon (200 mg, 10% wt. equivalent) were placed in a two-necked round-bottomed flask under hydrogen at atmospheric pressure. Methanol (20 ml) was added and the reaction was stirred for 16 h at room temperature. After completion, the reaction was poured through a 1.5 inch pad of celite to remove the palladium catalyst. The celite plug was washed with methanol (3 x 50 ml) and then the combined filtrate and washes were concentrated in vacuo. The desired product was isolated as a reddish orange solid and used directly in the next step without further purification (1.730 g, 99%). ^{1}H NMR (500 MHz, $CDCl_3$): δ 7.28 (d, *J* = 7.9 Hz, 1H), 7.01 (td, *J* = 7.6 Hz, 1.4 Hz, 1H), 6.79 (m, 2H), 6.23 (br, 1H), 3.74 (br, 2H), 1.52 (s, 9H). ^{13}C NMR (125 MHz, $CDCl_3$): δ 153.79, 139.89, 126.12, 124.77, 124.65, 119.62, 117.60, 80.51, 28.32. ESI-HRMS: calcd. for $C_{11}H_{16}N_2O_2$: $[M+H]^+$ = *m/z* 209.1285, found: $[M+H]^+$ = *m/z* 209.1293.

Azido (2-(4-nitrophenyl)cyclopropyl)methanone (16):

4-Nitrostyrene (5.000 g, 33.44 mmol) and rhodium acetate dimer (0.150 g, 0.33 mmol) were placed in a round-bottomed flask under argon and dry dichloromethane (20 ml) was added to it. A solution of ethyl diazoacetate (3.900 ml, 36.79 mmol) in dry dichloromethane (140 ml) was added to the reaction mixture over a period of 6 h under argon atmosphere at room temperature. After complete addition, the reaction mixture was stirred overnight. After completion as evidenced by TLC, the volatiles were removed under reduced pressure and the crude was purified by automated column chromatography (SiO_2, 5% EtOAc/hexanes) to obtain ethyl 2-(4-nitrophenyl)cyclopropanecarboxylate as a yellow liquid

(3.820 g, 40%, a mixture of cis-trans isomers). The mixture of geometric isomers was used directly in the next reaction without further purification.

Ethyl 2-(4-nitrophenyl)cyclopropanecarboxylate (2.900 g, 12.33 mmol) was placed in in a round-bottomed flask, a mixture of solvents (THF:MeOH:H_2O, 10 ml each) was added, and the reaction mixture was cooled to 0 ºC. Reagent grade LiOH (1.470 g, 6.64 mmol) was added to the reaction mixture which caused the color to turn dark brown at the beginning, but the color faded to light brown over time. The reaction mixture was stirred at room temperature for 4 h. After completion as evidenced by TLC, the reaction was acidified to pH 2 using an ice cold aqueous solution of 5% HCl. Then, the reaction mixture was extracted with dichloromethane (3 x 40 ml). The combined organic extracts were washed with water (20 ml), brine (20 ml), dried with anhydrous sodium sulfate, filtered, and concentrated under reduced pressure to obtain crude 2-(4-nitrophenyl)cyclopropanecarboxylic acid (2.120 g, 83%). This crude acid was subjected to the next reaction without further purification.

2-(4-Nitrophenyl)cyclopropanecarboxylic acid (0.250 g, 1.21 mmol) was placed in a round-bottomed flask and cooled to 0 ºC. Thionyl chloride (0.620 ml, 8.45 mmol) was added dropwise and the reaction mixture was refluxed for 1 h. After reflux, the excess thionyl chloride was removed under reduced pressure and a crude yellow material was obtained. The crude material was dissolved in acetone (4 ml) and kept at 0 ºC. A solution of NaN_3 (0.160 g, 2.41 mmol) in water (0.6 ml) was added dropwise to the reaction mixture at 0 ºC and stirred for 30 min at the same temperature. After completion as shown by TLC, acetone was removed under reduced pressure and the reaction mixture was extracted with dichloromethane (3 x 10 ml), the combined organic layers were washed with brine (10 ml), dried over anhydrous sodium sulfate, filtered, and concentrated under reduced pressure to obtain the crude azide as light yellow solid. The geometric isomers (cis/trans) were separated by automated column chromatography (SiO_2, 50% EtOAc/hexanes). The less polar trans isomer **16** was obtained as an off-white solid (0.140 g, 50%). ^{1}H NMR (500 MHz, $CDCl_3$): δ 8.14 (d, J = 8.6 Hz, 2H), 7.22 (d, J = 8.6 Hz, 2H), 2.52 (m, 1H), 1.94 (m, 1H), 1.66 (m, 1H), 1.32 (m, 1H). ^{13}C NMR (125 MHz, $CDCl_3$): δ 178.72,

147.02, 126.85, 123.89, 123.83, 27.67, 27.49, 19.06. ESI-HRMS: calcd. for $C_{10}H_8N_4O_3$: $[M-N_2]^-$ = *m/z* 203.0457, found: $[M-N_2]^-$ = *m/z* 203.0458.

***tert*-Butyl 2-(4-nitrophenyl)cyclopropylcarbamate (17):**

The trans azide **16** (1.900 g, 8.12 mmol) was placed in a round-bottomed flask, dry toluene (40 ml) was added to it, and the reaction was heated at 100 ºC for 2 h under argon atmosphere until the bubbling of N_2 gas stopped. After completion of the reaction, toluene was removed under reduced pressure to obtain the crude material. Dry *tert*-butanol (50 ml) was added and the reaction mixture was heated to reflux for 2 h. After completion as evidenced by TLC, *tert*-butanol was removed under reduced pressure to obtain an off-white color crude material which was washed with diethyl ether (10 ml) to yield pure compound **17** as a white solid (1.800 g, 79%). ^{1}H NMR (500 MHz, DMSO-d_6): δ 8.10 (d, *J* = 8.8 Hz, 2H), 7.34 (d, *J* = 8.8 Hz, 2H), 2.76 (br, 1H), 2.06 (m, 1H), 1.37 (s, 9H), 1.31 (m, 2H). ^{13}C NMR (125 MHz, DMSO-d_6): δ 155.88, 150.63, 145.32, 126.61, 123.35, 78.05, 34.81, 28.17, 24.66, 16.60. ESI-HRMS: calcd. for $C_{14}H_{18}N_2O_4$: $[M+Na]^+$ = *m/z* 301.1164, found: $[M+Na]^+$ = *m/z* 301.1169.

***tert*-Butyl 2-(4-aminophenyl)cyclopropylcarbamate (18):**

The nitro compound **17** (0.300 g, 1.08 mmol), K_2CO_3 (0.212 g, 1.53 mmol) and 10% palladium on carbon (0.012 g) were placed in a round-bottomed flask after which tetrahydrofuran (3 ml) and water (3 ml) were added to the reaction mixture. The reaction was degassed with argon for 5 min. Then, a solution of sodium hypophosphite monohydrate (0.870 g, 1.19 mmol) in water (2 ml) was added dropwise with vigorous stirring over a period of 15 min. After complete addition, the reaction mixture was heated at 60 ºC for 1.5 h. After completion as evidenced by TLC, the reaction mixture was filtered through a celite pad to remove the catalyst and the pad was washed with ethyl acetate. Water (5 ml) was added to the filtrate and the organic products were extracted with ethyl acetate (3 x 10 ml). The combined organic layers were washed with brine, dried over anhydrous sodium sulfate, filtered, and concentrated under reduced pressure to obtain the crude amine as a brown gummy solid. The crude mixture was purified by automated column chromatography (SiO_2, 45% EtOAc/hexanes) to yield pure

amine **18** as a brown, sticky oil (0.198 g, 74%). ^{1}H NMR (500 MHz, CD_3OD): δ 6.89 (d, J = 8.3 Hz, 2H), 6.63 (d, J = 8.3 Hz, 2H), 2.51 (br, 1H), 1.86 (br, 1H), 1.44 (s, 9H), 0.99 (m, 2H). ^{13}C NMR (125 MHz, CD_3OD): δ 159.14, 146.47, 132.18, 128.29, 116.88, 80.19, 33.23, 28.92, 24.84, 15.91. ESI-HRMS: calcd. for $C_{14}H_{20}N_2O_2$: $[M+H]^+$ = m/z 249.1603, found: $[M+H]^+$ = m/z 249.1606.

Ethyl 4-(4-hydroxybut-1-ynyl)benzoate (19):

Dry dichloromethane (100 ml) was added to a round-bottomed flask and degassed with argon for 10 min. Ethyl 4-bromobenzoate (7.000 g, 30.56 mmol), 3-butyn-1-ol (5.800 ml, 76.40 mmol), $PdCl_2$ (0.271 g, 1.53 mmol), PPh_3 (0.800 g, 3.06 mmol), Et_2NH (12.7 ml, 122.24 mmol) were added to solvent and the reaction was degassed again for 10 min. Finally, CuI (0.582 g, 3.06 mmol) was added and the reaction color changed from light yellow to dark brown. The reaction mixture was stirred at room temperature for 1 h under argon atmosphere. After completion as evidenced by TLC, the reaction mixture was filtered through a celite pad to remove the catalyst and the pad was washed with dichloromethane. Water (100 ml) was added to the filtrate, the organic layer was isolated, and the organic products were further extracted with dichloromethane (2 x 80 ml). The combined organic layers were washed with brine (100 ml), dried over anhydrous sodium sulfate, filtered, and concentrated under reduced pressure to obtain a dark purple crude which was purified by automated column chromatography (SiO_2, 60% EtOAc/hexanes) to yield alcohol **19** as a thick purple liquid (6.000 g, 89%). ^{1}H NMR (500 MHz, $CDCl_3$): δ 7.96 (d, J = 8.0 Hz, 2H), 7.46 (d, J = 8.0 Hz, 2H), 4.37 (q, J = 7.2 Hz, 2H), 3.84, (t, J = 6.3 Hz, 2H), 2.72 (t, J = 6.3 Hz, 2H), 1.39 (t, J = 7.2 Hz, 3H). ^{13}C NMR (125 MHz, $CDCl_3$): δ 166.09, 131.51, 129.54, 129.35, 127.97, 89.72, 81.76, 61.08, 60.98, 23.84, 14.25. EI-HRMS: calcd. for $C_{13}H_{14}O_3$: $[M]^+$ = m/z 218.0943, found: $[M]^+$ = m/z 218.0943.

Ethyl 4-(4-hydroxybutyl)benzoate (20):

The alcohol **19** (1.950 g, 8.93 mmol) was dissolved in ethanol (120 ml), 10% palladium on carbon (0.390 g) was added to it, and the reaction mixture was subjected to hydrogenation using a Parr shaker apparatus under H_2 atmosphere (80 psi) for 24 h at room temperature. After completion as evidenced

by TLC, the reaction mixture was filtered through a celite pad to remove the catalyst and the pad was washed with ethanol. The filtrate was concentrated under reduced pressure to obtain pure alcohol **20** as a yellow liquid (1.620 g, 82%). ^{1}H NMR (500 MHz, $CDCl_3$): δ 7.95 (d, *J* = 8.0 Hz, 2H), 7.24 (d, *J* = 8.5 Hz, 2H), 4.36 (q, *J* = 7.4 Hz, 2H), 3.66 (t, *J* = 6.4 Hz, 2H), 2.70 (t, *J* = 7.4 Hz, 2H), 1.72 (m, 2H), 1.61 (m, 2H), 1.38 (t, *J* = 7.4 Hz, 3H). ^{13}C NMR (125 MHz, $CDCl_3$): δ 166.68, 147.74, 129.61, 128.35, 128.10, 62.61, 60.76, 35.64, 32.19, 27.22, 14.32. ESI-HRMS: calcd. for $C_{13}H_{18}O_3$: $[M+Na]^+$ = *m/z* 245.1154, found: $[M+Na]^+$ = *m/z* 245.1156.

4-(4-(Ethoxycarbonyl)phenyl)butanoic acid (**21**):

A solution of compound **20** (2.000 g, 8.99 mmol) in dry dichloromethane (30 ml) was added to a mixture of pyridinium chlorochromate (2.910 g, 13.50 mmol) and NaOAc (0.740 g, 8.99 mmol) in dry dichloromethane (60 ml) in a round-bottomed flask. After complete addition, the reaction mixture was stirred at room temperature for 4 h. After completion as evidenced by TLC, diethyl ether (100 ml) was added to the reaction mixture and stirred for another 1 h. The residue was filtered through a celite pad and the pad was washed with diethyl ether several times. The filtrate was concentrated under reduced pressure to yield ethyl 4-(4-oxybutyl)benzoate as a light yellow liquid (1.400 g, 70%). Since the aldehyde was very unstable, it was used in the next step without further purification.

The aldehyde (1.300 g, 5.90 mmol) was dissolved in acetonitrile (7 ml) and cooled to 0 ºC. A solution of sodium dihydrogen phosphate monohydrate (0.240 g, 1.77 mmol) in water (4 ml) was added to the reaction at 0 ºC followed by the slow addition of a 30% aqueous hydrogen peroxide solution (0.900 ml, 7.08 mmol). A solution of sodium chlorite (0.800 g, 8.85 mmol) in water (12 ml) was added over a period of 1.5 h at 0 ºC during which oxygen release was observed. The reaction mixture was stirred for an additional 1 h. After completion as evidenced by TLC, sodium sulfite (0.900 g) was added to quench hypochlorous acid and residual hydrogen peroxide. The reaction mixture was acidified to pH 2 with 1N HCl and extracted with ethyl acetate (3 x 40 ml). The combined organic layers were washed with brine (50 ml), dried over anhydrous sodium sulfate, filtered, and concentrated under reduced

pressure to obtain the acid **21** (1.130 g, 81%) as a pure, colorless, low melting solid. ^{1}H NMR (500 MHz, $CDCl_3$): δ 7.97 (d, *J* = 8.3 Hz, 2H), 7.26 (d, *J* = 8.3 Hz, 2H), 4.37 (q, *J* = 7.2 Hz, 2H), 2.74 (t, *J* = 7.6 Hz, 2H), 2.39 (t, *J* = 7.4 Hz, 2H), 1.99 (q, *J* = 7.5 Hz, 2H), 1.39 (t, *J* = 7.2 Hz, 3H). ^{13}C NMR (125 MHz, $CDCl_3$): δ 179.07, 166.64, 146.54, 129.73, 128.43, 128.40, 60.85, 34.94, 33.2, 25.82, 14.31. ESI-HRMS: calcd. for $C_{13}H_{16}O_4$: $[M-H]^-$ = *m/z* 235.0970, found: $[M-H]^-$ = *m/z* 235.0969.

Ethyl 4-(4-(4-(2-(*tert*-butoxycarbonyl)cyclopropyl)phenylamino)-4-oxobutyl)benzoate (22): The amino compound **18** (0.090 g, 0.36 mmol), the acid **21** (0.085 g, 0.36 mmol), and HATU (0.163 g, 0.43 mmol) were placed in a round-bottomed flask. Dry dichloromethane (1 ml) was added and the reaction mixture was cooled to 0 ºC. DIEA (0.130 ml, 0.72 mmol) was added to the reaction at 0 ºC and the reaction was stirred at room temperature for 4 h. The color of the reaction changed from light yellow to orange. After completion as evidenced by TLC, water (3 ml) was added and the organic products were extracted with dichloromethane (3 x 4 ml). The combined organic extracts were washed with brine (5 ml), dried over anhydrous sodium sulfate, filtered, and concentrated under reduced pressure to obtain the crude amide which was purified by automated column chromatography (SiO_2, 40% EtOAc/hexanes) to yield pure amide **22** as a brown, gummy liquid (0.120 g, 71%). ^{1}H NMR (500 MHz, $CDCl_3$): δ 8.04 (br, 1H), 7.92 (d, *J* = 8.2 Hz, 2H), 7.38 (d, *J* = 8.2 Hz, 2H), 7.20 (d, *J* = 8.2 Hz, 2H), 6.99 (d, *J* = 8.2 Hz, 2H), 4.34 (q, *J* = 7.1 Hz, 2H), 2.68 (t, *J* = 7.5 Hz, 2H), 2.30 (t, *J* = 7.5 Hz, 2H), 2.00 (quin, *J* = 7.2 Hz, 2H), 1.96 (br, 1H), 1.43 (s, 9H), 1.36 (t, *J* = 7.1 Hz, 3H), 1.06 (t, *J* = 6.8 Hz, 2H). ^{13}C NMR (125 MHz, $CDCl_3$): δ 170.86, 166.57, 156.35, 146.89, 136.34, 136.08, 129.56, 128.33, 128.10, 126.72, 119.84, 79.41, 60.73, 36.29, 34.99, 32.24, 28.28, 26.47, 24.29, 16.05, 14.21. ESI-HRMS: calcd. for $C_{27}H_{34}N_2O_5$: $[M+Na]^+$ = *m/z* 489.2365, found: $[M+Na]^+$ = *m/z* 489.2365.

4-(4-(4-(2-(*tert*-Butoxycarbonyl)cyclopropyl)phenylamino)-4-oxobutyl)benzoic acid (23): The amide **22** (0.075 g, 0.16 mmol) was placed in in a round-bottomed flask, a mixture of solvents (THF:MeOH:H_2O, 0.3 ml each) was added, and the reaction mixture was cooled to 0 ºC. Reagent grade LiOH (0.020 g, 0.80 mmol) was added to the reaction mixture which caused the color to turn

brown which subsequently lightened over time. The reaction mixture was stirred at room temperature for 4 h. After completion as evidenced by TLC, the reaction was acidified on ice with an ice cold 5% aqueous solution of HCl to pH 2. Then, the reaction mixture was extracted with dichloromethane (3 x 4 ml). The combined organic extracts were washed with water (4 ml), brine (4 ml), dried with anhydrous sodium sulfate, filtered, and concentrated under reduced pressure to obtain crude acid **23** as a red solid (0.056 g, 80%). This crude acid was subjected to the next reaction without any purification. ^{1}H NMR (500 MHz, CD_3OD): δ 7.94 (d, *J* = 8.2 Hz, 2H), 7.43 (d, *J* = 8.5 Hz, 2H), 7.33 (d, *J* = 8.2 Hz, 2H), 7.07 (d, *J* = 8.5 Hz, 2H), 2.76 (t, *J* = 7.5 Hz, 2H), 2.60 (br, 1H), 2.39 (t, *J* = 7.5 Hz, 2H), 2.03 (quin, *J* = 7.5 Hz, 2H), 1.95 (br, 1H), 1.44 (s, 9H), 1.09 (t, *J* = 7.5 Hz, 2H). ^{13}C NMR (125 MHz, CD_3OD): δ 174.02, 170.03, 159.22, 148.87, 138.53, 137.87, 131.11, 129.80, 127.71, 121.38, 120.80, 80.42, 37.31, 36.38, 33.66, 28.90, 28.34, 25.18, 16.38. ESI-HRMS: calcd. for $C_{25}H_{30}N_2O_5$: $[M-H]^-$ = *m/z* 437.2076, found: $[M-H]^-$ = *m/z* 437.2077.

***tert*-Butyl 2-(4-(4-(4-(2-(*tert*-butoxycarbonyl)cyclopropyl)phenylamino)-4-oxobutyl)benzamido)phenyl carbamate (24):**

The protected amine **15** (0.019 g, 0.09 mmol), the acid **23** (0.040 g, 0.36 mmol), and HATU (0.042 g, 0.11 mmol) were placed in a round-bottomed flask. Dry dichloromethane (0.5 ml) was added to the mixture after which it was cooled to 0 ºC. DIEA (0.03 ml, 0.18 mmols) was added to the reaction at 0 ºC and the reaction was stirred at room temperature for 4 h. The color of the reaction changed from light yellow to orange. After completion as evidenced by TLC, water (3 ml) was added and the reaction mixture was extracted with dichloromethane (3 x 4 ml). The combined organic extracts were washed with brine (5 ml), dried over anhydrous sodium sulfate, filtered, and concentrated under reduced pressure to obtain the crude amide which was purified by automated column chromatography (SiO_2, 60% EtOAc/hexanes) to yield pure amide **24** as a red solid (0.044 g, 78%). ^{1}H NMR (500 MHz, $CDCl_3$): δ 8.31 (br, 1H), 7.96 (br, 1H), 7.81 (d, *J* = 8.0 Hz, 2H), 7.66 (m, 1H), 7.48 (br, 1H), 7.34 (dd, *J* = 5.7 Hz, 3.5 Hz, 2H), 7.15 (d, *J* = 8.0 Hz, 2H), 7.11 (m, 2H), 6.95 (d, *J* = 8.2 Hz, 2H), 2.64 (t, *J* = 7.4 Hz,

2H), 2.59 (br, 1H), 2.23 (m, 2H), 1.96 (m, 3H), 1.47 (s, 9H), 1.43 (s, 9H), 1.05 (t, *J* = 6.0 Hz, 2H). ^{13}C NMR (125 MHz, $CDCl_3$): δ 171.12, 165.93. 162.47, 156.44, 154.49, 145.88, 136.21, 136.18, 131.78, 130.46, 130.31, 128.50, 127.50, 126.70, 125.53, 125.28, 124.28, 119.83, 80.90, 79.48, 34.86, 32.22, 31.34, 28.30, 28.18, 26.57, 24.32, 15.92. ESI-HRMS: calcd. for $C_{36}H_{44}N_4O_6$: $[M-H]^-$ = *m/z* 627.3183, found: $[M-H]^-$ = *m/z* 627.3185.

4-(4-(4-(2-Aminocyclopropyl)phenylamino)-4-oxobutyl)-*N*-(2-aminophenyl)benzamide (**2, corin, TFA salt**):

The compound **24** (0.500 g, 0.80 mmol) was dissolved in dry dichloromethane (15 ml) and kept under argon atmosphere. Thiophenol (0.250 ml, 2.40 mmol) was added to the solution and the reaction mixture was placed in an ice bath. TFA (1.700 ml, 22.40 mmol) was added slowly to the reaction at 0 ºC and the reaction color changed from yellow to red. The reaction mixture was stirred at room temperature for 2 h. After completion as evidenced by TLC, the volatiles were removed and the crude material was purified by preparatory HPLC. The pure product **2** was obtained as a light yellow solid (0.205 g, 60%). ^1H NMR (500 MHz, CD_3OD): δ 7.95 (d, *J* = 8.0 Hz, 2H), 7.50 (d, *J* = 8.6 Hz, 2H), 7.40 (d, *J* = 8.0 Hz, 2H), 7.34 (d, *J* = 7.7 Hz, 1H), 7.28 (t, *J* = 7.3 Hz, 1H), 7.17 (br, 1H), 7.12 (d, *J* = 8.5 Hz, 3H), 2.80 (m, 3H), 2.42 (t, *J* = 7.4 Hz, 2H), 2.32 (ddd, *J* = 10.0 Hz, 6.6 Hz, 3.6 Hz, 1H), 2.05 (quin, *J* = 7.5 Hz, 2H), 1.37 (ddd, *J* = 10.3 Hz, 6.3 Hz, 4.4 Hz, 1H), 1.30 (q, *J* = 6.8 Hz, 1H). ^{13}C NMR (125 MHz, CD_3OD): δ 174.34, 174.19, 148.23, 138.79, 135.56, 132.71, 131.47, 129.98, 129.43, 128.73, 127.94, 127.69, 126.75, 123.05, 121.82, 121.61, 37.33, 36.31, 32.02, 28.41, 22.26, 13.78. ESI-HRMS: calcd. for $C_{26}H_{28}N_4O_2$: $[M+Na]^+$ = *m/z* 451.2110, found: $[M+Na]^+$ = *m/z* 451.2102.

4-(4-Bromophenyl)butanoic acid (25):

3-(4-Bromobenzoyl)propionic acid (5.142 g, 20.00 mmol) and potassium hydroxide (2.693 g, 48.00 mmol) were placed in a round-bottomed flask fitted with a condenser and a Dean-Stark apparatus and suspended in diethylene glycol (50 ml) at room temperature. Hydrazine (1.508 ml, 48.00 mmol) was slowly added to the reaction which was subsequently heated to 120-130 °C for 2 h upon which the

reaction became homogenous. After 2 h, the temperature was increased to 180-200 °C and stirring was continued for 3 h in order to distill off the remaining hydrazine and water byproduct via the Dean-Stark trap. Then, the reaction was allowed to cool to room temperature, diluted with water (20 ml), and carefully poured into aqueous 2.5 M HCl (40 ml). The precipitate that formed was collected by filtration and residual diethylene glycol was removed by dissolving the precipitate in a saturated, aqueous solution of potassium carbonate (40 ml). This solution was diluted with water (40 ml) and carefully poured into aqueous 2.5 M HCl (40 ml). A white precipitate formed which was collected by filtration, washed with water (2 x 30 ml), and dried under vacuum to yield the desired product as a white solid (0.886 g, 89%). ^{1}H NMR (500 MHz, DMSO-d_6): δ 12.07 (br, 1H), 7.46 (d, J = 8.3 Hz, 2H), 7.15 (d, J = 8.5 Hz, 2H), 2.56 (t, J = 7.6 Hz, 2H), 2.20 (t, J = 7.4 Hz, 2H), 1.77 (quin, J = 7.5 Hz, 2H). ^{13}C NMR (125 MHz, DMSO-d_6): δ 174.15, 140.99, 131.11, 130.59, 118.82, 33.68, 32.94, 26.05. ESI-HRMS: calcd. for $C_{10}H_{11}BrO_2$: $[M-H]^-$ = *m/z* 240.9870, found: $[M-H]^-$ = *m/z* 240.9882.

4-(4-Bromophenyl)-*N*-[4-(2-hydroxyethyl)phenyl]butanamide (26):

4-(4-Bromophenyl)butanoic acid **25** (1.702 g, 7.00 mmol), 2-(4-aminophenyl)ethanol (0.960 g, 7.00 mmol), and HATU (3.194 g, 8.40 mmol) were placed in a round-bottomed flask under argon and dissolved in anhydrous methylene chloride (30 ml). The reaction was cooled to 0 °C in an ice bath and triethylamine (1.179 ml, 15.40 mmol) was added after which the reaction became homogenous. The reaction was allowed to warm to room temperature and stirring was continued for 4 h. After completion, the reaction was poured into 1 N HCl (15 ml) and the organic products were extracted with methylene chloride (3 x 30 ml). The combined organic extracts were washed with brine, dried with anhydrous sodium sulfate, filtered, and concentrated in vacuo. Purification by recrystallization from ethyl acetate facilitated by the dropwise addition of hexanes provided the desired product as an off-white solid (2.272 g, 90%). ^{1}H NMR (500 MHz, DMSO-d_6): δ 9.77 (s, 1H), 7.47 (d, J = 8.2 Hz, 4H), 7.18 (d, J = 8.5 Hz, 2H), 7.11 (d, J = 8.5 Hz, 2H), 3.55 (t, J = 7.2 Hz, 2H), 2.65 (t, J = 7.2 Hz, 2H), 2.59 (t, J = 7.5 Hz, 2H), 2.28 (t, J = 7.4 Hz, 2H), 1.86 (quin, J = 7.5 Hz, 2H). ^{13}C NMR (125 MHz, DMSO-d_6): δ 170.55, 141.10,

137.18, 134.02, 131.10, 130.64, 128.92, 119.01, 118.80, 62.27, 38.46, 35.51, 33.87, 26.50. ESI-HRMS: calcd. for $C_{18}H_{20}BrNO_2$: $[M-H]^-$ = *m/z* 360.0605, found: $[M-H]^-$ = *m/z* 360.0608.

4-(4-Bromophenyl)-*N*-[4-(2-{[*tert*-butyl(dimethyl)silyl]oxy}ethyl)phenyl]butanamide (27): 4-(4-Bromophenyl)-*N*-[4-(2-hydroxyethyl)phenyl]butanamide **26** (2.174 g, 6.00 mmol) was dissolved in anhydrous methylene chloride (20 ml) and to it was added 4-dimethylaminopyridine (0.073 g, 0.60 mmol) and triethylamine (2.091 ml, 15.00 mmol). The reaction was stirred until all solids were dissolved after which *tert*-butyldimethylsilyl chloride (1.085 g, 7.20 mmol) was dissolved in anhydrous methylene chloride (10 ml) and added to the reaction in one portion. The reaction was stirred at room temperature for 2 h and then poured into water (30 ml). The organic layer was isolated and the aqueous layer was further extracted with methylene chloride (2 x 20 ml). The combined organic extracts were washed with brine (20 ml), dried with anhydrous sodium sulfate, filtered, and concentrated in vacuo. Purification by column chromatography (SiO_2, 10% EtOAc/hexanes) yielded the desired product as a slightly yellow, viscous oil (2.288 g, 80%). ^1H NMR (500 MHz, $CDCl_3$): δ 7.40 (m, 4H), 7.26 (br, 1H), 7.15 (d, *J* = 8.3 Hz, 2H), 7.06 (d, *J* = 8.2 Hz, 2H), 3.78 (t, *J* = 7.1 Hz, 2H), 2.79 (t, *J* = 7.0 Hz, 2H), 2.66 (t, *J* = 7.5 Hz, 2H), 2.32 (t, *J* = 7.4 Hz, 2H), 2.03 (quin, *J* = 7.4 Hz, 2H), 0.88 (s, 9H), 0.00 (s, 6H). ^{13}C NMR (125 MHz, $CDCl_3$): δ 170.58, 140.30, 135.90, 135.24, 131.43, 130.21, 129.59, 119.73, 119.71, 64.42, 38.95, 36.46, 34.41, 26.62, 25.90, 18.29, -5.41. ESI-HRMS: calcd. for $C_{24}H_{34}BrNO_2Si$: $[M+H]^+$ = *m/z* 476.1615, found: $[M+H]^+$ = *m/z* 476.1633.

Methyl (2*E*)-3-[4-(4-{[4-(2-{[*tert*-butyl(dimethyl)silyl]oxy}ethyl)phenyl]amino}-4-oxobutyl)phenyl]prop-2-enoate (28): 4-(4-Bromophenyl)-*N*-[4-(2-{[*tert*-butyl(dimethyl)silyl]oxy}ethyl)phenyl]butanamide **27** (1.430 g, 3.00 mmol), methyl prop-2-enoate (0.544 ml, 6.00 mmol), palladium(II) acetate (0.067 g, 0.30 mmol), triphenylphosphine (0.157 g, 0.60 mmol), and *N*,*N*,*N′*,*N′*-tetramethylethane-1,2-diamine (0.449 ml, 3.00 mmol) were placed in a sealed tube and dissolved in toluene (5 ml) under argon. The reaction was heated to 130 °C and stirred for 48 h after which it was cooled to room temperature and diluted with

methylene chloride (50 ml). Then, the organic layer was washed with water (3 x 20 ml), brine (15 ml), dried with anhydrous sodium sulfate, filtered through a 1.5 inch pad of celite, and the celite pad was washed with methylene chloride (3 x 20 ml). The combined filtrate and washes were concentrated in vacuo and the brown residue obtained was purified by column chromatography (SiO_2, 10-25% EtOAc/hexanes) to yield the desired product as a light yellow solid (0.834 g, 58%). ^{1}H NMR (500 MHz, $CDCl_3$): δ 7.68 (d, *J* = 16.0 Hz, 1H), 7.46 (d, *J* = 8.2 Hz, 2H), 7.41 (d, *J* = 8.3 Hz, 2H), 7.23 (d, *J* = 8.0 Hz, 2H), 7.16 (d, *J* = 8.5 Hz, 2H), 7.10 (s, 1H), 6.41 (d, *J* = 15.9 Hz, 1H), 3.81 (s, 3H), 3.78 (t, *J* = 7.1 Hz, 2H), 2.78 (t, *J* = 7.1 Hz, 2H), 2.74 (t, *J* = 7.5 Hz, 2H), 2.35 (t, *J* = 7.4 Hz, 2H), 2.08 (quin, *J* = 7.5 Hz, 2H), 0.88 (s, 9H), 0.01 (s, 6H). ^{13}C NMR (125 MHz, $CDCl_3$): δ 170.49, 167.55, 144.67, 144.13, 135.89, 135.27, 132.30, 129.63, 129.08, 128.23, 119.66, 117.05, 64.44, 51.66, 38.97, 36.58, 34.92, 26.55, 25.91, 18.31, -5.40. ESI-HRMS: calcd. for $C_{28}H_{39}NO_4Si$: $[M+H]^+$ = *m/z* 482.2721, found: $[M+H]^+$ = *m/z* 482.2725.

Methyl (2*E*)-3-[4-(4-{[4-(2-hydroxyethyl)phenyl]amino}-4-oxobutyl)phenyl]prop-2-enoate (29): Methyl (2*E*)-3-[4-(4-{[4-(2-{[*tert*-butyl(dimethyl)silyl]oxy}ethyl)phenyl]amino}-4-oxobutyl)phenyl]prop-2-enoate **28** (0.834 g, 1.73 mmol) was dissolved in anhydrous tetrahydrofuran (5 ml) and to it was added a 1 M solution of tetra-*n*-butylammonium fluoride (5.19 ml, 5.19 mmol). The reaction was stirred for 16 h after which it was complete as evidenced by TLC. The reaction was then poured into a mixture of water (15 ml) and methylene chloride (15 ml). The organic layer was isolated and the aqueous layer was further extracted with methylene chloride (2 x 15 ml). The combined organic extracts were washed with brine (15 ml), dried with anhydrous sodium sulfate, filtered, and concentrated in vacuo. Purification by column chromatography (SiO_2, 25-100% EtOAc/hexanes) afforded the desired product as a white solid (0.555 g, 87%). ^{1}H NMR (500 MHz, $CDCl_3$): δ 7.68 (d, *J* = 16.0 Hz, 1H), 7.46 (d, *J* = 8.2 Hz, 2H), 7.43 (d, *J* = 8.5 Hz, 2H), 7.23 (d, *J* = 8.0 Hz, 2H), 7.19 (d, *J* = 8.3 Hz, 2H), 7.12 (br, 1H), 6.41 (d, *J* = 16.0 Hz, 1H), 3.84 (t, *J* = 6.4 Hz, 2H), 3.81 (s, 3H), 2.84 (t, *J* = 6.5 Hz, 2H), 2.74 (t, *J* = 7.5 Hz, 2H), 2.35 (t, *J* = 7.4 Hz, 2H), 2.08 (quin, *J* = 7.4 Hz, 2H). ^{13}C NMR (125 MHz, $CDCl_3$): δ 170.60, 167.56,

144.67, 144.08, 136.21, 134.51, 132.33, 129.55, 129.09, 128.24, 120.11, 117.07, 63.62, 51.68, 38.56, 36.56, 34.92, 26.55. ESI-HRMS: calcd. for $C_{22}H_{25}NO_4$: $[M+H]^+$ = *m/z* 368.1856, found: $[M+H]^+$ = *m/z* 368.1863.

Methyl (2*E*)-3-[4-(4-{[4-(2-bromoethyl)phenyl]amino}-4-oxobutyl)phenyl]prop-2-enoate (30): Methyl (2*E*)-3-[4-(4-{[4-(2-hydroxyethyl)phenyl]amino}-4-oxobutyl)phenyl]prop-2-enoate **29** (0.555 g, 1.51 mmol) and triphenylphosphine (0.595 g, 2.27 mmol) were placed in a round-bottomed flask under argon and dissolved in anhydrous methylene chloride (3 ml). Then, tetrabromomethane (0.753 g, 2.27 mmol) was dissolved in anhydrous methylene chloride (2 ml) and added dropwise to the reaction at room temperature after which the reaction turned from an opaque mixture to a homogenous, yellow solution. The reaction was stirred for 30 min and after completion, it was poured into water (15 ml) and the organic products were extracted with methylene chloride (3 x 10 ml). The combined organic extracts were washed with brine (10 ml), dried with anhydrous sodium sulfate, filtered, and concentrated in vacuo. Purification by column chromatography (SiO_2, 25% EtOAc/hexanes) afforded the desired product as a white solid (0.474 g, 73%).^{1}H NMR (500 MHz, $CDCl_3$): δ 7.66 (d, *J* = 16.0 Hz, 1H), 7.44 (m, 5H), 7.20 (d, *J* = 8.2 Hz, 2H), 7.15 (d, *J* = 8.5 Hz, 2H), 6.40 (d, *J* = 16.0 Hz, 1H), 3.81 (s, 3H), 3.53 (t, *J* = 7.5 Hz, 2H), 3.12 (t, *J* = 7.5 Hz, 2H), 2.72 (t, *J* = 7.5 Hz, 2H), 2.35 (t, *J* = 7.3 Hz, 2H), 2.06 (quin, *J* = 7.4 Hz, 2H). ^{13}C NMR (125 MHz, $CDCl_3$): δ 170.74, 167.55, 144.65, 144.07, 136.65, 134.73, 132.22, 129.15, 129.02, 128.18, 119.98, 116.98, 51.65, 38.68, 36.53, 34.89, 32.98, 26.52. ESI-HRMS: calcd. for $C_{22}H_{24}BrNO_3$: $[M+H]^+$ = *m/z* 430.1012, found: $[M+H]^+$ = *m/z* 430.1031.

Di-*tert*-butyl 1-(2-{4-[(4-{4-[(1*E*)-3-methoxy-3-oxoprop-1-en-1-yl]phenyl}butanoyl)amino]phenyl}ethyl)hydrazine-1,2-dicarboxylate (31):

The title compound was prepared from methyl (2*E*)-3-[4-(4-{[4-(2-bromoethyl)phenyl]amino}-4-oxobutyl)phenyl]prop-2-enoate **30** (1.871 g, 4.35 mmol) following a procedure similar to that used for **13**. Purification by column chromatography (25-50% EtOAc/hexanes) yielded the desired product as a viscous oil that solidified to a white solid on standing overnight (1.818 g, 72%). ^{1}H NMR (500 MHz,

$CDCl_3$): δ 7.68 (d, *J* = 16.0 Hz, 1H), 7.46 (d, *J* = 8.0 Hz, 2H), 7.42 (d, *J* = 8.2 Hz, 2H), 7.23 (d, *J* = 7.9 Hz, 2H), 7.15 (br, 2H), 7.10 (s, 1H), 6.41 (d, *J* = 16.0 Hz, 1H), 3.81 (s, 3H), 3.66 (br, 2H), 2.85 (br, 2H), 2.74 (t, *J* = 7.5 Hz, 2H), 2.35 (t, *J* = 7.3 Hz, 2H), 2.08 (quin, *J* = 7.4 Hz, 2H), 1.48 (s, 9H), 1.44 (s, 9H). ESI-HRMS: calcd. for $C_{32}H_{43}N_3O_7$: $[M-H]^-$ = *m/z* 580.3028, found: $[M-H]^-$ = *m/z* 580.3048.

***N*-[4-(2-Hydrazinylethyl)phenyl]-4-{4-[(1*E*)-3-(hydroxyamino)-3-oxoprop-1-en-1-yl]phenyl}butanamide (3, HCl salt):**

An aqueous solution of hydroxylamine (50 wt %, 10 ml) was placed on ice and to it was added sodium hydroxide (1.002 g, 25.04 mmol). The solution was stirred until the sodium hydroxide was completely dissolved after which di-*tert*-butyl 1-(2-{4-[(4-{4-[(1*E*)-3-methoxy-3-oxoprop-1-en-1-yl]phenyl}butanoyl)amino]phenyl}ethyl)hydrazine-1,2-dicarboxylate **31** (1.818 g, 3.13 mmol) was dissolved in a 1:1 solution of tetrahydrofuran/methanol (20 ml) and added dropwise to the reaction at 0 °C. The reaction was then allowed to warm to room temperature and stirring was continued for an additional 30 min. After completion, the reaction was quenched with glacial acetic acid (1.433 ml, 25.04 mmol) and further acidified with a 10% citric acid solution (30 ml). The organic products were extracted with ethyl acetate (3 x 30 ml) and the combined organic extracts were washed with brine (30 ml), dried over anhydrous sodium sulfate, filtered, and concentrated in vacuo. Di-*tert*-butyl 1-(2-{4-[(4-{4-[(*1E*)-3-(hydroxyamino)-3-oxoprop-1-en-1-yl]phenyl}butanoyl)amino]phenyl}ethyl)hydrazine-1,2-dicarboxylate was obtained as a viscous, yellow oil and used without further purification.

The hydroxamic acid was then taken up in methylene chloride (19 ml) and to it was added trifluoroacetic acid (1 ml). The reaction was stirred at room temperature for 16 h after which it was complete as evidenced by TLC. Then, the reaction was concentrated in vacuo and the residue obtained was taken up in *N*,*N*-dimethylformamide and purified by preparatory HPLC. The ditrifluoroacetic acid salt was isolated as a white solid (0.932 g, 49%). To prepare the dihydrochloride salt, anhydrous methanol (20 ml) was cooled to 0 °C in an ice bath under argon and to it was added acetyl chloride (1.570 ml, 21.96 mmol) dropwise to generate hydrochloric acid in situ. The reaction was

stirred for 15 min after which the ditrifluoroacetic acid salt was taken up in anhydrous methanol (10 ml) and added dropwise at 0 °C. The reaction was stirred for an additional 30 min and then concentrated to approximately one third of the original volume. The reaction was then placed on ice and the desired dihydrochloride salt was precipitated by the dropwise addition of diethyl ether and isolated by filtration as a white solid (0.556 g, 80%). ^{1}H NMR (500 MHz, CD_3OD/DMSO-d_6): δ 7.53 (m, 5H), 7.29 (d, J = 8.0 Hz, 2H), 7.23 (d, J = 8.5 Hz, 2H), 6.45 (d, J = 15.9 Hz, 1H), 3.25 (t, J = 7.9 Hz, 2H), 2.92 (t, J = 7.8 Hz, 2H), 2.73 (t, J = 7.5 Hz, 2H), 2.40 (t, J = 7.4 Hz, 2H), 2.02 (quin, J = 7.5 Hz, 2H). ^{13}C NMR (125 MHz, CD_3OD/DMSO-d_6): δ 173.77, 166.12, 145.46, 141.55, 139.09, 134.11, 134.03, 130.42, 130.29, 129.18, 121.59, 117.94, 53.54, 37.33, 36.21, 32.18, 28.37. ESI-HRMS: calcd. for $C_{21}H_{26}N_4O_3$: $[M+H]^+$ = m/z 383.2078, found: $[M+H]^+$ = m/z 383.2096.

***N*-[4-(2-Hydroxyethyl)phenyl]-4-(4-nitrophenyl)butanamide (32):**

4-(4-Nitrophenyl)butanoic acid (2.092 g, 10.00 mmol), 2-(4-aminophenyl)ethanol (1.372 g, 10.00 mmol), and HATU (4.563 g, 12.00 mmol) were placed in a round-bottomed flask under argon and dissolved in anhydrous methylene chloride (30 ml). The reaction was cooled to 0 °C in an ice bath after which triethylamine (2.800 ml, 20.00 mmol) was added. The reaction was allowed to warm to room temperature and stirring was continued for 4 h during which time it became homogenous. After completion, it was poured into 1 N HCl (15 ml) and the organic products were extracted with methylene chloride (3 x 15 ml). The combined organic extracts were washed with brine (20 ml), dried with anhydrous sodium sulfate, filtered, and concentrated in vacuo. Recrystallization from ethyl acetate provided the desired product as a white solid (2.978 g, 91%). ^{1}H NMR (500 MHz, DMSO-d_6): δ 9.79 (s, 1H), 8.16 (d, J = 8.8 Hz, 2H), 7.51 (d, J = 8.8 Hz, 2H), 7.46 (d, J = 8.5 Hz, 2H), 7.11 (d, J = 8.6 Hz, 2H), 4.60 (br, 1H), 3.55 (m, 2H), 2.76 (t, J = 7.6 Hz, 2H), 2.65 (t, J = 7.2 Hz, 2H), 2.31 (t, J = 7.4 Hz, 2H), 1.93 (quin, J = 7.5 Hz, 2H). ^{13}C NMR (125 MHz, DMSO-d_6): δ 170.44, 150.23, 145.87, 137.16, 134.09, 129.67, 128.95, 123.45, 119.04, 62.29, 38.47, 35.48, 34.35, 26.20. ESI-HRMS: calcd. for $C_{18}H_{20}N_2O_4$: $[M+H]^+$ = m/z 329.1496, found: $[M+H]^+$ = m/z 329.1501.

4-(4-Aminophenyl)-*N*-[4-(2-hydroxyethyl)phenyl]butanamide (33):

N-[4-(2-Hydroxyethyl)phenyl]-4-(4-nitrophenyl)butanamide **32** (2.978 g, 9.07 mmol) and 10% palladium on carbon (0.300 g, 20% wt. equivalent) were placed in a two-necked round-bottomed flask under a H_2 atmosphere. Ethanol (50 ml) was added followed by acetic acid (0.300 ml). The reaction was stirred overnight at room temperature and then poured through a 1.5 inch pad of celite which was subsequently washed with methanol (3 x 30 ml). The combined filtrate and washes were concentrated in vacuo and the solid obtained was purified via recrystallization from ethyl acetate to yield the title compound as a beige solid (2.160 g, 80%). ^{1}H NMR (500 MHz, DMSO-d_6): δ 9.75 (s, 1H), 7.47 (d, *J* = 8.5 Hz, 2H), 7.11 (d, *J* = 8.5 Hz, 2H), 6.84 (d, *J* = 8.3 Hz, 2H), 6.49 (d, *J* = 8.3 Hz, 2H), 4.81 (s, 2H), 4.59 (t, *J* = 5.0 Hz, 1H), 3.55 (m, 2H), 2.65 (t, *J* = 7.2 Hz, 2H), 2.43 (t, *J* = 7.5 Hz, 2H), 2.25 (t, *J* = 7.5 Hz, 2H), 1.79 (quin, *J* = 7.5 Hz, 2H). ^{13}C NMR (125 MHz, DMSO-d_6): δ 170.88, 146.47, 137.27, 133.95, 128.92, 128.66, 128.56, 118.99, 114.00, 62.29, 38.47, 35.79, 33.88, 27.29. ESI-HRMS: calcd. for $C_{18}H_{22}N_2O_2$: $[M+H]^+$ = *m/z* 299.1754, found: $[M+H]^+$ = *m/z* 299.1765.

Methyl 8-{[4-(4-{[4-(2-hydroxyethyl)phenyl]amino}-4-oxobutyl)phenyl]amino}-8-oxooctanoate (34):

4-(4-Aminophenyl)-*N*-[4-(2-hydroxyethyl)phenyl]butanamide **33** (1.492 g, 5.00 mmol), suberic acid monomethyl ester (0.941 g, 5.00 mmol), and HATU (2.282 g, 6.00 mmol) were dissolved in a 4:1 mixture of anhydrous methylene chloride (40 ml) and anhydrous *N*,*N*-dimethylformamide (10 ml) and cooled to 0 °C in an ice bath. Triethylamine (1.530 ml, 11.00 mmol) was added and then the reaction was allowed to warm to room temperature with stirring for 4 h. Then, the reaction was poured into an aqueous 1 N HCl solution (20 ml) and the organic products were extracted with methylene chloride (4 x 30 ml). The combined organic extracts were washed with 1 N HCl (3 x 15 ml), brine (15 ml), dried with anhydrous sodium sulfate, filtered, and concentrated in vacuo. Purification by trituration in methanol yielded the desired product as a white solid (1.616 g, 69%). ^{1}H NMR (500 MHz, DMSO-d_6): δ 9.77 (s, 2H), 7.49 (d, *J* = 8.5 Hz, 2H), 7.47 (d, *J* = 8.5 Hz, 2H), 7.11 (m, 4H), 4.59 (t, *J* = 5.2 Hz, 1H), 3.57 (s,

3H), 3.56 (m, 2H), 2.65 (t, J = 7.2 Hz, 2H), 2.55 (t, J = 7.5 Hz, 2H), 2.27 (m, 6H), 1.85 (quin, J = 7.5 Hz, 2H), 1.55 (m, 4H), 1.29 (m, 4H). ^{13}C NMR (125 MHz, DMSO-d_6): δ 173.33, 170.96, 170.70, 137.23, 137.19, 136.15, 133.99, 128.92, 128.43, 119.12, 119.00, 62.28, 51.15, 38.47, 36.28, 35.67, 34.02, 33.22, 28.30, 28.22, 26.86, 24.98, 24.31. ESI-HRMS: calcd. for $C_{27}H_{36}N_2O_5$: $[M+H]^+$ = m/z 469.2697, found: $[M+H]^+$ = m/z 469.2712.

Methyl 6-{[4-(4-{[4-(2-hydroxyethyl)phenyl]amino}-4-oxobutyl)phenyl]amino}-6-oxohexanoate (35):

The title compound was synthesized from 4-(4-aminophenyl)-*N*-[4-(2-hydroxyethyl)phenyl]butanamide **33** (0.500 g, 1.68 mmol) and adipic acid monomethyl ester (0.248 ml, 1.68 mmol) using a procedure similar to that used to prepare **34**. Purification by column chromatography (2-5% MeOH/DCM) yielded the desired product as a white solid (0.517 g, 70%). ^{1}H NMR (500 MHz, CD_3OD): δ 7.45 (m, 4H), 7.16 (m, 4H), 3.72 (t, J = 7.1 Hz, 2H), 3.65 (s, 3H), 2.77 (t, J = 7.1 Hz, 2H), 2.65 (t, J = 7.5 Hz, 2H), 2.37 (m, 6H), 1.98 (quin, J = 7.5 Hz, 2H), 1.69 (m, 4H). ^{13}C NMR (125 MHz, CD_3OD): δ 175.78, 174.33, 174.21, 138.99, 138.12, 137.93, 136.34, 130.40, 129.96, 121.62, 121.54, 64.36, 52.17, 39.80, 37.62, 37.33, 35.83, 34.60, 28.74, 26.47, 25.71. ESI-HRMS: calcd. for $C_{25}H_{32}N_2O_5$: $[M+H]^+$ = m/z 441.2384, found: $[M+H]^+$ = m/z 441.2405.

Methyl 8-{[4-(4-{[4-(2-bromoethyl)phenyl]amino}-4-oxobutyl)phenyl]amino}-8-oxooctanoate (36):

Methyl 8-{[4-(4-{[4-(2-hydroxyethyl)phenyl]amino}-4-oxobutyl)phenyl]amino}-8-oxooctanoate **34** (0.469 g, 1.00 mmol) and triphenylphosphine (0.394 g, 1.50 mmol) were placed in a round-bottomed flask under argon at room temperature. Anhydrous methylene chloride (1.5 ml) was added followed by tetrabromomethane (0.498 g, 1.50 mmol) dropwise as a solution in anhydrous methylene chloride (0.5 ml). Stirring was continued for 30 min at room temperature after which the reaction was poured into water (15 ml) and the organic products extracted with methylene chloride (3 x 15 ml). The combined organic extracts were washed with brine, dried with anhydrous sodium sulfate, filtered, and concentrated in vacuo. Purification by trituration in methanol yielded the title compound as a white solid

(383 mg, 72%). ^{1}H NMR (500 MHz, DMSO-d_6): δ 9.82 (s, 1H), 9.77 (s, 1H), 7.50 (t, *J* = 8.9 Hz, 4H), 7.17 (d, *J* = 8.3 Hz, 2H), 7.11 (d, *J* = 8.5 Hz, 2H), 3.68 (t, *J* = 7.3 Hz, 2H), 3.57 (s, 3H), 3.05 (t, *J* = 7.2 Hz, 2H), 2.55 (t, *J* = 7.5 Hz, 2H), 2.28 (m, 6H), 1.85 (quin, *J* = 7.5 Hz, 2H), 1.54 (m, 4H), 1.29 (m, 4H). ^{13}C NMR (125 MHz, DMSO-d_6): δ 173.33, 170.96, 170.83, 137.90, 137.20, 136.13, 133.39, 128.89, 128.43, 119.12, 119.06, 51.15, 37.86, 36.28, 35.68, 34.65, 34.01, 33.22, 28.30, 28.22, 26.83, 24.98, 24.31. ESI-HRMS: calcd. for $C_{27}H_{35}BrN_2O_4$: $[M+H]^+$ = *m/z* 531.1853, found: $[M+H]^+$ = *m/z* 531.1876.

Methyl 6-{[4-(4-{[4-(2-bromoethyl)phenyl]amino}-4-oxobutyl)phenyl]amino}-6-oxohexanoate (37): The title compound was synthesized from methyl 6-{[4-(4-{[4-(2-hydroxyethyl)phenyl]amino}-4-oxobutyl)phenyl]amino}-6-oxohexanoate **35** (0.515 g, 1.17 mmol) using a procedure similar to that used to prepare **36**. Purification by trituration in methanol yielded the desired product as a white solid (0.401 g, 68%). ^{1}H NMR (500 MHz, DMSO-d_6): δ 9.82 (s, 1H), 9.79 (s, 1H), 7.50 (t, *J* = 8.9 Hz, 4H), 7.17 (d, *J* = 8.5 Hz, 2H), 7.12 (d, *J* = 8.3 Hz, 2H), 3.67 (t, *J* = 7.3 Hz, 2H), 3.58 (s, 3H), 3.05 (t, *J* = 7.2 Hz, 2H), 2.56 (t, *J* = 7.5 Hz, 2H), 2.33 (t, *J* = 7.1 Hz, 2H), 2.28 (m, 4H), 1.85 (quin, *J* = 7.5 Hz, 2H), 1.57 (m, 4H). ^{13}C NMR (125 MHz, DMSO-d_6): δ 173.23, 170.84, 170.73, 137.90, 137.15, 136.20, 133.40, 128.90, 128.45, 119.14, 119.07, 51.21, 37.87, 35.96, 35.68, 34.65, 34.02, 33.04, 26.83, 24.60, 24.06. ESI-HRMS: calcd. for $C_{25}H_{31}BrN_2O_4$: $[M+H]^+$ = *m/z* 503.1540, found: $[M+H]^+$ = *m/z* 503.1552.

Di-*tert*-butyl 1-(2-{4-[(4-{4-[(8-methoxy-8-oxooctanoyl)amino]phenyl}butanoyl)amino]phenyl}ethyl)hydrazine-1,2-dicarboxylate (38): The title compound was prepared from methyl 8-{[4-(4-{[4-(2-bromoethyl)phenyl]amino}-4-oxobutyl)phenyl]amino}-8-oxooctanoate **36** (0.266 g, 0.50 mmol) following a procedure similar to that used for **13**. Purification by column chromatography (SiO_2, 10-50% EtOAc/hexanes) provided the desired product as a white solid (0.083 g, 24%). ^{1}H NMR (500 MHz, $CDCl_3$): δ 7.54 (br, 1H), 7.45 (m, 3H), 7.38 (d, *J* = 8.3 Hz, 2H), 7.12 (d, *J* = 7.9 Hz, 2H), 7.09 (d, *J* = 8.2 Hz, 2H), 3.67 (s, 3H), 3.66 (br, 2H), 2.84 (t, *J* = 7.3 Hz, 2H), 2.65 (t, *J* = 7.2 Hz, 2H), 2.33 (m, 4H), 2.25 (t, *J* = 7.5 Hz, 2H), 2.00 (quin,

J = 7.3 Hz, 2H), 1.73 (quin, *J* = 7.3 Hz, 2H), 1.64 (m, 2H), 1.48 (s, 9H), 1.44 (br, 9H), 1.38 (m, 4H). ESI-HRMS: calcd. for $C_{37}H_{54}N_4O_8$: $[M+H]^+$ = *m/z* 683.4014, found: $[M+H]^+$ = *m/z* 683.3999.

Di-*tert*-butyl 1-(2-{4-[(4-{4-[(6-methoxy-6-oxohexanoyl)amino]phenyl}butanoyl)amino]phenyl}ethyl)hydrazine-1,2-dicarboxylate (39):

The title compound was prepared from methyl 6-{[4-(4-{[4-(2-bromoethyl)phenyl]amino}-4-oxobutyl)phenyl]amino}-6-oxohexanoate **37** (0.400 g, 0.79 mmol) following a procedure similar to that used for **13**. Purification by column chromatography (SiO_2, 25-75% EtOAc/hexanes) provided the desired product as a white solid (0.292 g, 56%). ^1H NMR (500 MHz, $CDCl_3$): δ 8.21 (br, 1H), 8.14 (s, 1H), 7.45 (br, 2H), 7.33 (d, *J* = 8.3 Hz, 2H), 7.08 (d, *J* = 8.3 Hz, 2H), 7.00 (d, *J* = 8.3 Hz, 2H), 3.65 (s, 3H), 3.63 (br, 2H), 2.81 (t, *J* = 7.5 Hz, 2H), 2.57 (t, *J* = 7.2 Hz, 2H), 2.34 (t, *J* = 6.9 Hz, 4H), 2.19 (t, *J* = 7.5 Hz, 2H), 1.93 (quin, *J* = 7.3 Hz, 2H), 1.69 (m, 4H), 1.46 (s, 9H), 1.42 (br, 9H). ESI-HRMS: calcd. for $C_{35}H_{50}N_4O_8$: $[M+H]^+$ = *m/z* 655.3701, found: $[M+H]^+$ = *m/z* 655.3715.

***N*-[4-(4-{[4-(2-hydrazinylethyl)phenyl]amino}-4-oxobutyl)phenyl]-*N*'-hydroxyoctanediamide (4, TFA salt):**

An aqueous solution of hydroxylamine (50 wt %, 1 ml) was placed on ice and to it was added sodium hydroxide (0.038 g, 0.96 mmol). The solution was stirred until the sodium hydroxide was completely dissolved after which di-*tert*-butyl 1-(2-{4-[(4-{4-[(8-methoxy-8-oxooctanoyl)amino]phenyl}butanoyl)amino]phenyl}ethyl)hydrazine-1,2-dicarboxylate **38** (0.083 g, 0.12 mmol) was dissolved in a 1:1 solution of tetrahydrofuran/methanol (4 ml) and added dropwise to the reaction at 0 °C. The reaction was then allowed to warm to room temperature and stirring was continued for an additional 30 min. After completion, the reaction was quenched with glacial acetic acid (0.055 ml, 0.96 mmol) and further acidified with a 10% aqueous citric acid solution (10 ml). The organic products were extracted with ethyl acetate (3 x 10 ml) and the combined organic extracts were washed with brine (10 ml), dried over anhydrous sodium sulfate, filtered, and concentrated in vacuo. Di-*tert*-butyl 1-[2-(4-{[4-(4-{[8-(hydroxyamino)-8-

oxooctanoyl]amino}phenyl)butanoyl]amino}phenyl)ethyl]hydrazine-1,2-dicarboxylate was obtained as a viscous, yellow oil and used without further purification.

The hydroxamic acid intermediate was taken up in methylene chloride (9.5 ml) and to it was added trifluoroacetic acid (0.5 ml). The reaction was stirred at room temperature for 16 h after which it was complete as evidenced by TLC. Then, the reaction was concentrated in vacuo and the residue obtained was taken up in *N*,*N*-dimethylformamide and purified by preparatory HPLC. The ditrifluoroacetic acid salt was isolated as a white solid (0.030 g, 35%). ^{1}H NMR (500 MHz, DMSO-d_6): δ 10.33 (s, 1H), 9.84 (s, 1H), 9.78 (s, 1H), 8.65 (br, 1H), 7.52 (d, *J* = 8.5 Hz, 2H), 7.49 (d, *J* = 8.5 Hz, 2H), 7.14 (d, *J* = 8.5 Hz, 2H), 7.11 (d, *J* = 8.5 Hz, 2H), 3.07 (br, 2H), 2.76 (br, 2H), 2.55 (t, *J* = 7.5 Hz, 2H), 2.27 (m, 4H), 1.93 (t, *J* = 7.4 Hz, 2H), 1.84 (quin, *J* = 7.5 Hz, 2H), 1.56 (m, 2H), 1.48 (m, 2H), 1.26 (m, 4H). ^{13}C NMR (125 MHz, CD_3OD /DMSO-d_6): δ 174.07, 173.85, 172.58, 139.13, 138.68, 138.23, 130.29, 130.00, 121.61, 121.40, 53.51, 37.96, 37.32, 35.78, 33.84, 32.25, 30.06, 30.00, 28.66, 26.85, 26.71. ESI-HRMS: calcd. for $C_{26}H_{37}N_5O_4$: $[M+H]^+$ = *m/z* 484.2918, found: $[M+H]^+$ = *m/z* 484.2941.

_N_-[4-(4-{[4-(2-Hydrazinylethyl)phenyl]amino}-4-oxobutyl)phenyl]-_N'_-hydroxyhexanediamide (5, TFA salt):

The title compound was synthesized from di-*tert*-butyl 1-(2-{4-[(4-{4-[(6-methoxy-6-oxohexanoyl)amino]phenyl}butanoyl)amino]phenyl}ethyl)hydrazine-1,2-dicarboxylate **39** (0.275 g, 0.42 mmol) using a procedure similar to that used to prepare **4**. Purification by preparatory HPLC provided the ditrifluoroacetic acid salt as a white solid (0.086 g, 30%). ^{1}H NMR (500 MHz, CD_3OD): δ 7.49 (d, *J* = 8.5 Hz, 2H), 7.45 (d, *J* = 8.5 Hz, 2H), 7.21 (d, *J* = 8.5 Hz, 2H), 7.16 (d, *J* = 8.3 Hz, 2H), 3.24 (t, *J* = 7.8 Hz, 2H), 2.90 (t, *J* = 7.8 Hz, 2H), 2.66 (t, *J* = 7.5 Hz, 2H), 2.37 (m, 4H), 2.14 (t, *J* = 6.8 Hz, 2H), 1.99 (quin, *J* = 7.4 Hz, 2H), 1.70 (m, 4H). ^{13}C NMR (125 MHz, CD_3OD /DMSO-d_6): δ 173.94, 173.78, 172.37, 139.08, 138.74, 138.19, 134.15 130.29, 130.03, 121.67, 121.41, 53.66, 37.69, 37.33, 35.80, 33.71, 32.28, 28.66, 26.52, 26.50. ESI-HRMS: calcd. for $C_{24}H_{33}N_5O_4$: $[M+H]^+$ = *m/z* 456.2605, found: $[M+H]^+$ = *m/z* 456.2621.

8-(4-(Ethoxycarbonyl)phenylamino)-8-oxooctanoic acid (40):

A solution of ethyl 4-aminobenzoate (1.000 g, 6.05 mmol) in anhydrous tetrahydrofuran (10 ml) was slowly added to a cooled (0 °C) solution of suberic anhydride (0.940 g, 6.05 mmol) and the resulting mixture was stirred at room temperature for 1.5 h. The solvent was removed under vacuum, a 2 N potassium carbonate solution (10 ml) was added to the residue, and the resulting basic solution was extracted with ethyl acetate (3 x 30 ml). The combined extracts were then acidified to pH 5 with 2 N HCl and the colorless precipitate was isolated by filtration, washed with water (3 x 10 ml), dried with anhydrous sodium sulfate, filtered, concentrated, and recrystallized from acetonitrile to obtain the title compound (1.320 g, 68%). mp: 153-155 °C. ^{1}H NMR (400 MHz, DMSO-d_6) δ 12.01 (s, 1H), 10.20 (br, 1H), 7.89 (d, J = 8.4 Hz, 2H), 7.73 (d, J = 8.4 Hz, 2H), 4.28 (q, J = 6.8 Hz, 2H), 2.34 (t, J = 7.2 Hz, 2H), 2.20 (t, J = 7.2 Hz, 2H), 1.55 (m, 4H), 1.31 (m, 7H). ^{13}C NMR (100 MHz, DMSO-d_6) δ 179.8, 178.4, 165.9, 142.8, 130.1, 125.7, 118.1, 60.9, 38.3, 34.0, 28.3, 24.7, 25.6, 14.1. EI-MS: calcd. for $C_{17}H_{23}NO_5$: $[M]^+$ = m/z 321.1576, found: $[M]^+$ = m/z 321.1580.

Ethyl 4-(8-oxo-8-(tetrahydro-2*H*-pyran-2-yloxyamino)octanamido)benzoate (41):

Ethyl chloroformate (0.110 ml, 1.12 mmol) and triethylamine (0.170 ml, 1.21 mmol) were added to a cooled (0 °C) solution of **40** (0.300 g, 0.93 mmol) in anhydrous tetrahydrofuran (10 ml), and the mixture was stirred for 30 min. The solid was filtered off, and *O*-(tetrahydro-2*H*-pyran-2-yl)hydroxylamine (0.330 g, 2.79 mmol) was added to the cooled (0 °C) filtrate. The solution was stirred for 1.5 h, then evaporated under vacuum and the residue was triturated with a 1:1 mixture of diethylether/petroleum ether, filtered, and dried to afford a colorless solid that was recrystallized from toluene to obtain pure compound **41** (0.285 g, 73%). mp: 138-140 °C. ^{1}H NMR (400 MHz, $CDCl_3$) δ 8.01 (d, J = 8.8 Hz, 2H), 7.67 (d, J = 8.4 Hz, 2H), 4.97 (m, 1H), 4.38 (q, J = 7.2 Hz, 2H), 3.95 (m, 1H), 3.64 (m, J = 11.2 Hz, 2H), 2.39 (t, J = 7.2 Hz, 2H), 2.15 (m, 2H), 1.50 (m, 17H). ^{13}C NMR (100 MHz, $CDCl_3$) δ 179.8, 169.9, 165.9, 142.8, 130.1, 125.7, 118.1, 109.7, 63.0, 60.9, 38.3, 32.8, 27.9, 27.6, 25.6, 25.4, 20.1, 14.1. EI-MS: calcd. for $C_{22}H_{32}N_2O_6$: $[M]^+$ = m/z 420.2260, found: $[M]^+$ = m/z 420.2264.

4-(8-Oxo-8-(tetrahydro-2*H*-pyran-2-yloxyamino)octanamido)benzoic acid (42):

A solution of **41** (0.170 g, 0.40 mmol) and lithium hydroxide monohydrate (0.067 g, 1.60 mmol) in a 1:1 mixture of tetrahydrofuran/water (6 ml) was stirred overnight at room temperature. After this time, the reaction was quenched with 2 N HCl, the pH was adjusted to 5, and the colorless solid was filtered, washed with water (3 x 10 ml), dried with anhydrous sodium sulfate, and recrystallized from acetonitrile to obtain the pure compound **42** (0.132 g, 84%). mp: 176-178 °C. ^{1}H NMR (400 MHz, DMSO-d_6) δ 12.68 (s, 1H), 10.88 (br, 1H), 10.16 (br, 1H), 7.87 (d, J = 8.4 Hz, 2H), 7.70 (d, J = 8.4 Hz, 2H), 4.80 (m, 1H), 3.92 (m, 1H), 3.49 (m, 1H), 2.33 (t, J = 7.2 Hz, 2H), 1.98 (t, J = 6.8 Hz, 2H), 1.59 (m, 10H), 1.25 (m, 4H). ^{13}C NMR (100 MHz, DMSO-d_6) δ 179.8, 169.9, 169.3, 143.7, 130.5, 125.8, 118.1, 109.7, 63.0, 38.3, 32.8, 27.9, 27.6, 25.6, 25.4, 20.1. EI-MS: calcd. for $C_{20}H_{28}N_2O_6$: $[M]^+$ = m/z 392.1947, found: $[M]^+$ = m/z 392.1943.

***tert*-Butyl 2-(4-(4-(8-oxo-8-(tetrahydro-2*H*-pyran-2-yloxyamino)octanamido)benzamido)phenyl)cyclopropylcarbamate (43):**

Triethylamine (0.100 ml, 0.68 mmol) and (benzotriazole-1-yloxy)tripyrrolidinophosphonium hexafluorophosphate (PyBOP) (0.109 g, 0.20 mmol) were added under nitrogen atmosphere to a solution of **42** (0.067 g, 0.17 mmol) in anhydrous *N,N*-dimethylformamide (2 ml), and the resulting mixture was stirred for 45 min. After this time, **18** (0.042 g, 0.17 mmol) was added and the stirring was continued overnight. The reaction was quenched with water (30 ml) and the precipitate was filtered, washed with water (3 × 10 ml), and dried. The solid residue was chromatographed on silica gel eluting with ethyl acetate to provide a colorless solid which was recrystallized from acetonitrile to yield pure **43** (0.071 g, 67%). mp: 185-187 °C. ^{1}H NMR (400 MHz, DMSO-d_6) δ 10.89 (s, 1H), 10.13 (s, 1H), 10.02 (br, 1H), 7.92 (d, J = 8.4 Hz, 2H), 7.72 (d, J = 8.0 Hz, 2H), 7.65 (d, J = 8.4 Hz, 2H), 7.07 (d, J = 9.2 Hz, 2H), 4.80 (br, 1H), 3.92 (m, 1H), 3.60 (m, 2H), 3.49 (m, 1H), 2.33 (t, J = 7.2 Hz, 2H), 1.98 (t, J = 6.8 Hz, 2H), 1.89 (m, 1H), 1.63 (m, 10H), 1.39 (s, 9H), 1.21 (m, 6H). ^{13}C NMR (100 MHz, DMSO-d_6) δ 179.8, 169.9, 164.7, 155.6, 141.9, 137.3, 134.3, 129.8, 129.6, 125.2, 121.7, 121.0, 109.7, 79.5, 63.0, 38.3,

32.8, 32.6, 28.4, 27.9, 27.6, 25.6, 25.4, 22.8, 20.1, 14.4. EI-MS: calcd. for $C_{34}H_{46}N_4O_7$: $[M]^+$ = *m/z* 622.3366, found: $[M]^+$ = *m/z* 622.3369.

N^1-(4-(4-(2-Aminocyclopropyl)phenylcarbamoyl)phenyl)-N^8-hydroxyoctanediamide hydrochloride (6):

A solution of **43** (0.030 g, 0.05 mmol) and 4 N HCl/dioxane (0.380 ml, 1.50 mmol) in tetrahydrofuran (2 ml) was stirred at room temperature overnight. The precipitated colorless solid was filtered, washed with diethyl ether (3 × 5 ml) and dried to afford title compound **6** as a colorless hydrochloride salt that was recrystallized from ethanol (0.018 g, 75%). mp: >250 °C. ^{1}H NMR (400 MHz, DMSO-d_6) δ 10.35 (s, 1H), 10.23 (br, 1H), 10.10 (br, 1H), 8.48 (br, 3H), 7.99 (d, 2H), 7.72 (m, 4H), 7.27 (m, 2H), 2.79 (m, 1H), 2.39 (m, 4H), 1.95 (m, 1H), 1.41 (m, 10H). ^{13}C NMR (100 MHz, DMSO-d_6) δ 179.8, 169.9, 164.7, 141.9, 138.9, 134.3, 129.8, 129.6, 125.2, 121.7, 121.0, 38.3, 32.5, 28.0, 27.9, 25.6, 22.0, 14.0. EI-MS: calcd. for $C_{24}H_{30}N_4O_4$: $[M]^+$ = *m/z* 438.2267, found: $[M]^+$ = *m/z* 438.2271.

***N*-(4-(2-Aminocyclopropyl)phenyl)-4-phenylbutanamide (7, TFA salt):**

The amino compound **18** (0.400 g, 1.61 mmol), 4-phenylbutyric acid (0.241 g, 1.46 mmol), and HATU (0.670 g, 1.75 mmol) were placed in a round-bottomed flask. Dry dichloromethane (8 ml) was added to the mixture after which it was cooled to 0 ºC. DIEA (0.500 ml, 2.92 mmol) was added to the reaction at 0 ºC and the reaction was stirred at room temperature for 4 h. The color of the reaction changed from light yellow to orange. After completion as evidenced by TLC, water (15 ml) was added to the reaction mixture and the organics were extracted with dichloromethane (3 x 4 ml). The combined organic extracts were washed with brine (15 ml), dried over anhydrous sodium sulfate, filtered, and concentrated under reduced pressure to obtain the crude amide which was used in the next step without further purification.

tert-Butyl 2-(4-(4-phenylbutanamido)phenyl)cyclopropylcarbamate (0.080 g, 0.21 mmol) was dissolved in dry dichloromethane (4 ml) and kept under argon atmosphere. Thiophenol (0.040 ml, 0.42 mmol)

was added to the solution and the reaction mixture was placed in an ice bath. TFA (0.200 ml, 2.54 mmol) was added slowly to the reaction at 0 ºC and the reaction color changed from yellow to red. The reaction mixture was stirred at 0 ºC for 2 h. After completion as evidenced by TLC, the volatiles were removed and the crude material was purified by preparatory HPLC. The pure product **7** was obtained as a hygroscopic light yellow solid (0.036 g, 60%). ^{1}H NMR (500 MHz, CD_3OD): δ 7.49 (d, *J* = 8.3 Hz, 2H), 7.26 (m, 2H), 7.20 (m, 2H), 7.16 (m, 1H), 7.11 (d, *J* = 8.3 Hz, 2H), 2.80 (dt, *J* = 7.8 Hz, 4.0 Hz, 1H), 2.68 (t, *J* = 7.6 Hz, 2H), 2.37 (t, *J* = 7.5 Hz, 2H), 2.32 (ddd, *J* = 10.1 Hz, 6.4 Hz, 3.6 Hz, 1H), 1.99 (quin, *J* = 7.5 Hz, 2H), 1.37 (m, 1H), 1.30 (q, *J* = 7.0 Hz, 1H). ^{13}C NMR (125 MHz, CD_3OD): δ 174.4, 143.0, 138.7, 135.4, 129.6, 129.5, 127.8, 127.0, 121.5, 37.3, 36.3, 31.9, 28.7, 22.2, 13.7. ESI-HRMS: calcd. for $C_{19}H_{22}N_2O$: $[M+H]^+$ = *m/z* 295.1810, found: $[M+H]^+$ = *m/z* 295.1819.

Ethyl 4-(4-(4-cyclopropylphenylamino)-4-oxobutyl)benzoate (44):

4-Cyclopropylaniline (0.15 g, 1.13 mmol), the acid **21** (0.266 g, 1.13 mmol) and HATU (0.514 g, 1.35 mmol) were placed in a round-bottomed flask. Dry CH_2Cl_2 (5 ml) was added to the mixture. DIEA (0.4 ml, 2.25 mmols) was added to the reaction at 0 ºC and the reaction was stirred at room temperature for 2 h. After completion as evidenced by TLC, water (10 ml) was added and the reaction mixture was extracted with CH_2Cl_2 (3 x 5 ml). The combined organic layers were washed with brine (8 ml), dried over anhydrous sodium sulfate, and concentrated under reduced pressure to obtain the crude amide which was purified by automated column chromatography (SiO_2, 30% EtOAc/hexanes) to yield pure amide **44** as a white solid (0.30 g, 75%). ^{1}H NMR (500 MHz, $CDCl_3$): 7.97 (d, *J* = 10 Hz, 2H), 7.39 (d, *J* = 5 Hz, 2H), 7.25 (d, *J* = 8.2 Hz, 2H), 7.02 (d, *J* = 10 Hz, 2H), 4.38 (q, *J* = 7.5 Hz, 2H), 2.33 (t, *J* = 5 Hz, 2H), 2.07 (quin, *J* = 7.5 Hz, 2H), 1.90-1.84 (m, 1H), 1.41 (t, *J* = 7.5 Hz, 3H), 0.94 (q, *J* = 5 Hz, 2H), 0.65 (q, *J* = 5 Hz, 2H). ^{13}C NMR (125 MHz, $CDCl_3$): δ 170.7, 166.7, 146.9, 140.0, 135.3, 129.9, 129.8, 128.5, 128.6, 126.2, 120.2, 120.1, 60.9, 36.5, 35.1, 26.6, 15.0, 14.4, 9.0. ESI-HRMS: calcd. for $C_{22}H_{25}NO_3$: $[M+H]^+$ = *m/z* 352.1913, found: $[M+H]^+$ = *m/z* 352.1909.

4-(4-(4-Cyclopropylphenylamino)-4-oxobutyl)benzoic acid (45):

The ester **44** (0.214 g, 0.61 mmol) was placed in a round-bottomed flask, a mixture of solvents (THF:MeOH:H_2O, 1.0 ml each) was added, and the reaction was cooled to 0 ºC. Reagent grade LiOH (0.072 g, 3.04 mmol) was then added to the reaction. The reaction mixture was stirred at room temperature for 4 h with the color changing from light yellow to yellow over time. After completion as evidenced by TLC, the reaction was acidified on ice with an aqueous solution of 5% HCl to pH 2. Then, the reaction mixture was extracted with CH_2Cl_2 (3 x 5 ml). The combined organic extracts were washed with water (8 ml), brine (8 ml), dried with anhydrous sodium sulfate, and concentrated under reduced pressure to obtain crude acid **45** (0.175 g, 90%) as a white solid. This crude acid was subjected to the next reaction without any purification. ^{1}H NMR (500 MHz, DMSO-d_6): δ 12.83 (br, 1H), 9.95 (s, 1H), 7.87 (d, *J* = 10 Hz, 2H), 7.48 (d, *J* = 10 Hz, 2H), 7.33 (d, *J* = 5 Hz, 2H), 6.97 (d, *J* = 5 Hz, 2H), 2.68 (t, *J* = 7.5 Hz, 2H), 2.32 (t, *J* = 7.5 Hz, 2H), 1.90 ((quin, *J* = 7.5 Hz, 2H), 1.86-1.81 (m, 1H), 0.88 (d, *J* = 5 Hz, 2H), 0.59 (d, J = 5 Hz, 2H). ^{13}C NMR (125 MHz, DMSO-d_6): δ 171.0, 167.8, 147.6, 138.5, 137.3, 129.9, 129.0, 125.9, 119.6, 36.1, 35.0, 27.0, 15.1, 9.5. ESI-HRMS: calcd. for $C_{20}H_{21}NO_3$: $[M+H]^+$ = *m/z* 324.1600, found: $[M+H]^+$ = *m/z* 324.1598.

***N*-(2-aminophenyl)-4-(4-(4-cyclopropylphenylamino)-4-oxobutyl)benzamide (9):**

The protected amine **15** (0.109 g, 0.53 mmol), the acid **45** (0.17 g, 0.53 mmol) and HATU (0.24 g, 0.63 mmol) were placed in a round-bottomed flask. Dry CH_2Cl_2 (5 ml) was added to the mixture. DIEA (0.2 ml, 1.05 mmol) was added to the reaction at 0 ºC and the reaction was stirred at room temperature for 2 h. The color of the reaction changed from colorless to light yellow. After completion as evidenced by TLC, water (8 ml) was added and the organic products were extracted with CH_2Cl_2 (3 x 5 ml). The combined organic layers were washed with brine solution (8 ml), dried over anhydrous sodium sulfate, and concentrated under reduced pressure to obtain the crude amide which was subjected to filter column purification and obtained as a white solid (0.16 g, 60%) which was used in the next step as a crude material.

tert-Butyl 2-(4-(4-(4-cyclopropylphenylamino)-4-oxobutyl)benzamido)phenylcarbamate (0.05 g, 0.10 mmol) was dissolved in dry CH_2Cl_2 (2 ml) and kept under argon atmosphere. Thiophenol (0.1 ml, 1.3 mmol) was added to the solution and the reaction mixture was placed in an ice bath. TFA (0.10 ml, 1.3 mmol) was added slowly to the reaction at 0 ºC and stirring was continued for 2 h at room temperature during which the reaction color changed to light yellow. After completion as evidenced by TLC, the volatiles were removed and the crude material was purified by preparatory HPLC. The pure product **9** (0.025 g, 62%) was obtained as white solid. ^1H NMR (500 MHz, CD_3OD): δ 7.94 (d, J = 5 Hz, 2H), 7.41-7.35 (m, 8H), 6.96 (d, J = 5 Hz, 2H), 2.75 (t, J = 7.5 Hz, 2H), 2.37(t, J = 7.5, Hz, 2H), 1.99 (quin, J = 7.5 Hz, 2H), 1.85-1.78 (m, 1H), 0.87 (d, J = 10 Hz, 2H), 0.57 (d, J = 5 Hz, 2H). ^{13}C NMR (125 MHz, CD_3OD): δ 172.5, 147.0, 139.9, 135.8, 131.0, 130.8, 128.5, 128.2, 128.0, 127.3, 126.2, 125.5, 123.2, 120.0, 35.8, 34.8, 27.0, 14.3, 8.0. ESI-HRMS: calcd. for $C_{26}H_{27}N_3O_2$: $[M+H]^+$ = *m/z* 414.2182, found: $[M+H]^+$ = *m/z* 414.2181.

SUPPLEMENTARY REFERENCES

1. Leaver-Fay, A. *et al*. An object-oriented software suite for the simulation and design of macromolecules. *Methods Enzymol.* **487**, 545-574 (2011).
2. Frisch, M. J. *et al*. Gaussian 09. *Gaussian 09, Revision B.01, Gaussian, Inc., Wallingford CT* (2009)
3. Becke, A. D. Density-functional thermochemistry. III. The role of exact exchange. *J. Chem. Phys.* **98,** 5648–5652 (1993).
4. Chipman, D. M. Reaction field treatment of charge penetration. *J. Chem. Phys.* **112,** 5558–5565 (2000).
5. Marenich, A. V., Jerome, S. V., Cramer, C. J. & Truhlar, D. G. Charge Model 5: An Extension of Hirshfeld Population Analysis for the Accurate Description of Molecular Interactions in Gaseous and Condensed Phases. *J. Chem. Theory Comput.* **8,** 527–541 (2012).
6. Chaudhury, S., Lyskov, S. & Gray, J. J. PyRosetta: a script-based interface for implementing molecular modeling algorithms using Rosetta. *Bioinformatics* **26,** 689–691 (2010).
7. Miteva, M. A., Guyon, F. & Tuffery, P. Frog2: Efficient 3D conformation ensemble generator for small compounds. *Nucleic Acids Res.* **38,** W622–W627 (2010).
8. Rohl, C. A., Strauss, C. E. M., Misura, K. M. S. & Baker, D. in *Methods in Enzymology* (eds. Ludwig, B. & Michael, L. J.) **383,** 66–93 (Academic Press, 2004).
9. Park, H. *et al*. Simultaneous optimization of biomolecular energy functions on features from small molecules and macromolecules. *J. Chem. Theory Comput.* **12**, 6201-6212 (2016).
10. Go, N., Noguti, T. & Nishikawa, T. Dynamics of a small globular protein in terms of low-frequency vibrational modes. *Proc. Natl. Acad. Sci.* **80,** 3696–3700 (1983).
11. Mandell, D. J., Coutsias, E. A. & Kortemme, T. Sub-angstrom accuracy in protein loop reconstruction by robotics-inspired conformational sampling. *Nat. Methods* **6,** 551–552 (2009).
12. Bhardwaj, G. *et al*. Accurate de novo design of hyperstable constrained peptides. *Nature* **538,** 329–335 (2016).
13. Leaver-Fay, A., Kuhlman, B. & Snoeyink, J. Rotamer-Pair Energy Calculations Using a Trie Data Structure. in *Algorithms in Bioinformatics. WABI 2005. Lecture Notes in Computer Science* (eds. Casadio, R. & Myers, G.) 389–400 (Springer, Berlin, Heidelberg, 2005). doi:10.1007/11557067_32.
14. Nocedal, J. & Wright, S. J. Numerical Optimization. (Springer, New York, 2006). doi:10.1007/978-0-387-40065-5.
15. Shechter, D., Dormann, H. L., Allis, C. D., & Hake, S. B. Extraction, purification and analysis of histones. *Nat. Protoc.* **2**, 1445-1457 (2007).
16. Taylor, E. C. & Harrington, P. M. A convergent synthesis of 5,10-dideaza-5,6,7,8-tetrahydrofolic acid and 5,10-dideaza-5,6,7,8-tetrahydrohomofolic acid - an effective principle for carbonyl group activation. *J. Org. Chem.* **55**, 3222-3227 (1990).
17. Dalcanale, E. & Montanari, F. Selective oxidation of aldehydes to carboxylic-acids with sodium-chlorite hydrogen-peroxide. *J. Org. Chem.* **51**, 567-569 (1986).
18. Tongkate, P., Pluempanupat, W. & Chavasiri, W. Hexabromoacetone and ethyl tribromoacetate: a highly efficient reagent for bromination of alcohol. *Tetrahedron Lett.* **49**, 1146-1148 (2008).
19. Wang, E. Y. *et al.* Design, synthesis, and biological evaluation of semicarbazide-sensitive amine oxidase (SSAO) inhibitors with anti-inflammatory activity. *J. Med. Chem.* **49**, 2166-2173 (2006).
20. Kelly, T. A. & Mcneil, D. W. A simple method for the protection of aryl amines as their t-butylcarbamoyl (Boc) derivatives. *Tetrahedron Lett.* **35**, 9003-9006 (1994).

21. Binda, C. *et al.* Biochemical, structural, and biological evaluation of tranylcypromine derivatives as inhibitors of histone demethylases LSD1 and LSD2. *J. Am. Chem. Soc.* **132**, 6827-6833 (2010).
22. Carroll, F. I. *et al.* Synthesis and biological evaluation of bupropion analogues as potential pharmacotherapies for cocaine addiction. *J. Med. Chem.* **52**, 6768-6781 (2009).
23. Walsh, T. F., Goulet, M. T., Ujjainwalla, F. & Bugianesi, R. L. Antagonists of gonadotropin releasing hormone. (1999).
24. Dieck, H. A. & Heck, R. F. Organophosphinepalladium complexes as catalysts for vinylic hydrogen substitution-reactions. *J. Am. Chem. Soc.* **96**, 1133-1136 (1974).
25. Davies, S. J., Moffat, D. F. C. & Testar, R. J. 2-(Hetero-)aryl,4-carbonyl substituted pyrazole derivatives as inhibitors of P38 mitogen-activated protein kinase. (2008).
26. Bergman, J. A. *et al.* Selective histone deacetylase 6 inhibitors bearing substituted urea linkers inhibit melanoma cell growth. *J. Med. Chem.* **55**, 9891-9899 (2012).
