# Supplementary material for: Targeting the CoREST complex with dual histone deacetylase and demethylase inhibitors
Source: Nat Commun. 2018 Jan 4;9:53. doi: 10.1038/s41467-017-02242-4 (PMC5754352; doi:10.1038/s41467-017-02242-4)
Supplement: Supplementary file 2 — Description of Additional Supplementary Files [file 41467_2017_2242_MOESM2_ESM.pdf]

Descriptions of Additional Supplementary Files:

File Name: Supplementary Dataset 1
Description: All quantified data in a repository
